# Yeast Rim11 kinase responds to glutathione-induced stress by regulating the transcription of phospholipid biosynthetic genes

**DOI:** 10.1091/mbc.E23-03-0116

**Published:** 2023-12-14

**Authors:** Taishi Yasukawa, Ryo Iwama, Yuriko Yamasaki, Naohisa Masuo, Yoichi Noda

**Affiliations:** a Mitsubishi Corporation Life Sciences Limited, Tokyo Takarazuka Building 14F, 1-1-3 Yurakucho, Chiyoda-ku, Tokyo 100-0006, Japan; b Collaborative Research Institute for Innovative Microbiology, Department of Biotechnology, The University of Tokyo, 1-1-1 Yayoi, Bunkyo-ku, Tokyo 113-8657, Japan; MRC Laboratory of Molecular Biology

## Abstract

Glutathione (GSH), a tripeptide composed of glycine, cysteine, and glutamic acid, is an abundant thiol found in a wide variety of cells, ranging from bacterial to mammalian cells. Adequate levels of GSH are essential for maintaining iron homeostasis. The ratio of oxidized/reduced GSH is strictly regulated in each organelle to maintain the cellular redox potential. Cellular redox imbalances cause defects in physiological activities, which can lead to various diseases. Although there are many reports regarding the cellular response to GSH depletion, studies on stress response to high levels of GSH are limited. Here, we performed genome-scale screening in the yeast *Saccharomyces cerevisiae* and identified *RIM11*, *BMH1*, and *WHI2* as multicopy suppressors of the growth defect caused by GSH stress. The deletion strains of each gene were sensitive to GSH. We found that Rim11, a kinase important in the regulation of meiosis, was activated via autophosphorylation upon GSH stress in a glucose-rich medium. Furthermore, RNA-seq revealed that transcription of phospholipid biosynthetic genes was downregulated under GSH stress, and introduction of multiple copies of *RIM11* counteracted this effect. These results demonstrate that *S*. *cerevisiae* copes with GSH stress via multiple stress-responsive pathways, including a part of the adaptive pathway to glucose limitation.

## Abstract (executive summary)

GSH is distributed throughout the cells. Cells cope with high level of GSH (GSH stress) by ER stress response, but it remains unclear whether cells have mechanism to alleviate GSH stress.

*RIM11* was identified as a multicopy suppressor gene in yeast that restores growth under GSH stress. Rim11 plays important roles in the regulation of phospholipid biosynthesis and meiosis. Glucose starvation response may also be involved in the GSH stress alleviation.

Our study identified a novel GSH stress tolerance mechanism that may contribute to efficient breeding of yeast strains with enhanced production of GSH for industrial applications.

## INTRODUCTION

The physiological activities of cells are supported by an electron transport system that is driven by redox networks. Known reduced/oxidized endogenous factors that constitute the redox networks include coenzymes, such as the reduced and oxidized forms of nicotinamide adenine dinucleotide (NADH/NAD^+^), nicotinamide adenine dinucleotide-phosphate (NADPH/NADP^+^), flavin mononucleotide (FMNH/FMN), Flavin adenine dinucleotide (FADH_2_/FAD), and coenzyme Q (CoQH_2_/CoQ); redox proteins, such as peroxiredoxins, glutaredoxin, and cytochrome; and low-molecular-weight (LMW) thiols, such as glutathione (GSH/GSSG) (Murphy, 2009; Xiao and Loscalzo, 2020). Most of the electron transport reactions in the cell are carried out by enzymatic or nonenzymatic redox reactions between the redox pairs GSH/GSSG, NADH/NAD^+^, and NADPH/NADP^+^ (Xiao *et al*., 2018; Xiao and Loscalzo, 2020).

GSH is a thiol tripeptide that is synthesized from glutamate, cysteine, and glycine via a two-step reaction catalyzed by glutamate-cysteine ligase and GSH synthetase. It is widely found in organisms ranging from Gram-negative and Gram-positive bacteria to mammalian cells, including humans (Pophaly *et al*., 2012). Knockout of the mouse gene encoding the glutamate-cysteine ligase causes embryonic lethality, and yeast and human cells cannot grow without exogenously added GSH, which is an essential molecule for normal proliferation of eukaryotic cells. Small quantities of GSH support the survival of yeast cells, and is attributed to its requirement for the cytosolic assembly of iron-sulfur clusters (Berndt and Lillig, 2017; Toledano and Huang, 2017).

GSH is maintained at a relatively high concentration (1–30 mM) intracellularly, and plays a central role in maintaining the redox balance in cells (Go and Jones, 2017). Simultaneously, the GSH/GSSG balance in each organelle is controlled by the activities of GSH synthetase, NADPH-dependent GSH reductase, and GSH transporters. For example, a high GSH/GSSG ratio is maintained in the mitochondrial matrix and cytosol, resulting in a reducing environment. In contrast, a relatively low GSH/GSSG ratio is maintained in the mitochondrial inner membrane, endoplasmic reticulum (ER), and peroxisomes resulting in an oxidative environment (Zechmann *et al*., 2011; Baudouin-Cornu *et al*., 2012; Toledano *et al*., 2013; Umezawa *et al*., 2017; Oestreicher and Morgan, 2019). Thus, organelle-specific biological reactions, such as the elimination of reactive oxygen species (ROS), formation of conjugates with heavy metals or toxic nucleophilic agents, and proper folding of nascent proteins in the ER, are regulated and supported by the mechanism that maintains the GSH/GSSG balance spatiotemporally.

This means that a GSH/GSSG imbalance can impair cellular activities, which in turn can lead to various human diseases (Go and Jones, 2017). Specifically, aging, heart disease (Handy and Loscalzo, 2017), cancer (Li *et al*., 2015), Type 2 diabetes (Hasnain *et al*., 2016), nonalcoholic fatty-liver disease (Yang *et al*., 2019), and neurodegenerative diseases (Dong *et al*., 2019) are associated with GSH deficiency. In addition, yeast strains in which the GSH biosynthetic pathway is disrupted show lower tolerance to a wide range of stresses (Izawa *et al*., 1995; Turton *et al*., 1997; Grant *et al*., 1998; Maris *et al*., 2000) and undergo apoptosis at a higher rate relative to the parental cells (Madeo *et al*., 1999).

Higher than normal levels of NADPH or GSH also impose reductive stress in cells and individuals. For example, overproduction of heat shock protein 27 in mice induces cardiac hypertrophy and dysfunction via increased GSH/GSSG ratio, elevation of glutathione peroxidase I, and decrease in Fe levels (Zhang *et al*., 2010). Reductive stress has also been reported to induce oxidative stress. For example, N-acetyl-l-cysteine treatment and overproduction of/or site-specific mutation in the γ-glutamylcysteine ligase in mammalian cells increases GSH levels by three- to fourfold, which in turn elevates the redox potential to 7–12 mV higher than the normal value. This shift to a reduced state causes excessive ROS production in the mitochondria, which induces oxidative stress and eventually leads to cell death (Zhang *et al*., 2012; Korge *et al*., 2015). Recently, several studies have investigated the reductive stress response from a pathological perspective like those mentioned above.

Studies focusing on the reductive stress induced by the direct addition of GSH from outside the cell are limited and only a few using a budding yeast have been reported (Kumar *et al*., 2011; Ponsero *et al*., 2017). Kumar *et al*. (2011) reported that excessive accumulation of GSH in yeast cells, mediated via plasma membrane-localized GSH transporter (Hgt1) overexpression, triggered stress similar to that caused by iron depletion and ER stress, and led to growth delay or cell death. In addition, the group reported that GSH entered the ER by facilitated diffusion through the ER-localized translocon Sec61. The transport of GSH by Sec61 was found to be regulated by ER-localized thiol oxidase Ero1 and Bip (Kar2; Ponsero *et al*., 2017). Ponsero *et al.* (2017) validated the existence of a specific mechanism in yeast in response to GSH stress. However, as GSH is widely distributed inside the cell and its levels are specifically maintained in each organelle, high level of GSH is suspected to affect cellular functions other than that in the ER.

In addition, GSH serves as a reservoir of sulfur metabolites, and plays pivotal roles in the sulfur metabolism. This is definitely reflected on the fact that intracellular contents of cysteine, cystathionine, and methionine increase in GSH-deficient yeast mutant (Elskens *et al*., 1991). In eukaryotic cell, methionine is utilized for biosynthesis of the phosphatidylcholine (PC) via S-adenosyl-methionine (SAM)-mediated methylation of phosphatidylethanolamine (Chin and Bloch, 1988; Hickman *et al*., 2011). Moreover, phosphatidylethanolamine is required for efficient biosynthesis of GSH and cysteine (Ye *et al*., 2017). We, thus, presumed that there would be some correlation between the levels of GSH and phospholipids in yeast cells.

Therefore, we sought to investigate whether cells have mechanisms, other than the ER stress response, to cope with GSH stress. Furthermore, we sought to identify yeast multicopy suppressor genes that rescue the growth defect in yeast cells treated with GSH.

## RESULTS

### Screen for multicopy suppressor genes that restore growth under GSH stress

To create a yeast strain sensitive to GSH stress, the *HGT1* gene that encodes the GSH transporter was placed under the control of a strong constitutive promoter (*TDH3pr*), which was then integrated into the genomes of *Saccharomyces cerevisiae* BY4741 and a deletion strain (Δ*ire1*), in which a gene encoding an ER stress sensor, *IRE1*, was deleted by double-crossover homologous recombination as described by Kumar *et al* (2011). These strains are hereafter referred to as HGT1 and Δ*ire1* HGT1 strains, respectively. Fluorescence microscopy of a strain overexpressing Hgt1 N-terminally fused with enhanced GFP confirmed the plasma membrane localization of Hgt1 in the HGT1 strain (Bourbouloux *et al*., 2000; Supplemental Figure S1A). Spotting cells on SC medium containing GSH confirmed that HGT and Δ*ire1* HGT1 strains showed higher and hypersensitivity to GSH relative to the parental strains, respectively (Supplemental Figure S1B).

**FIGURE 1:**
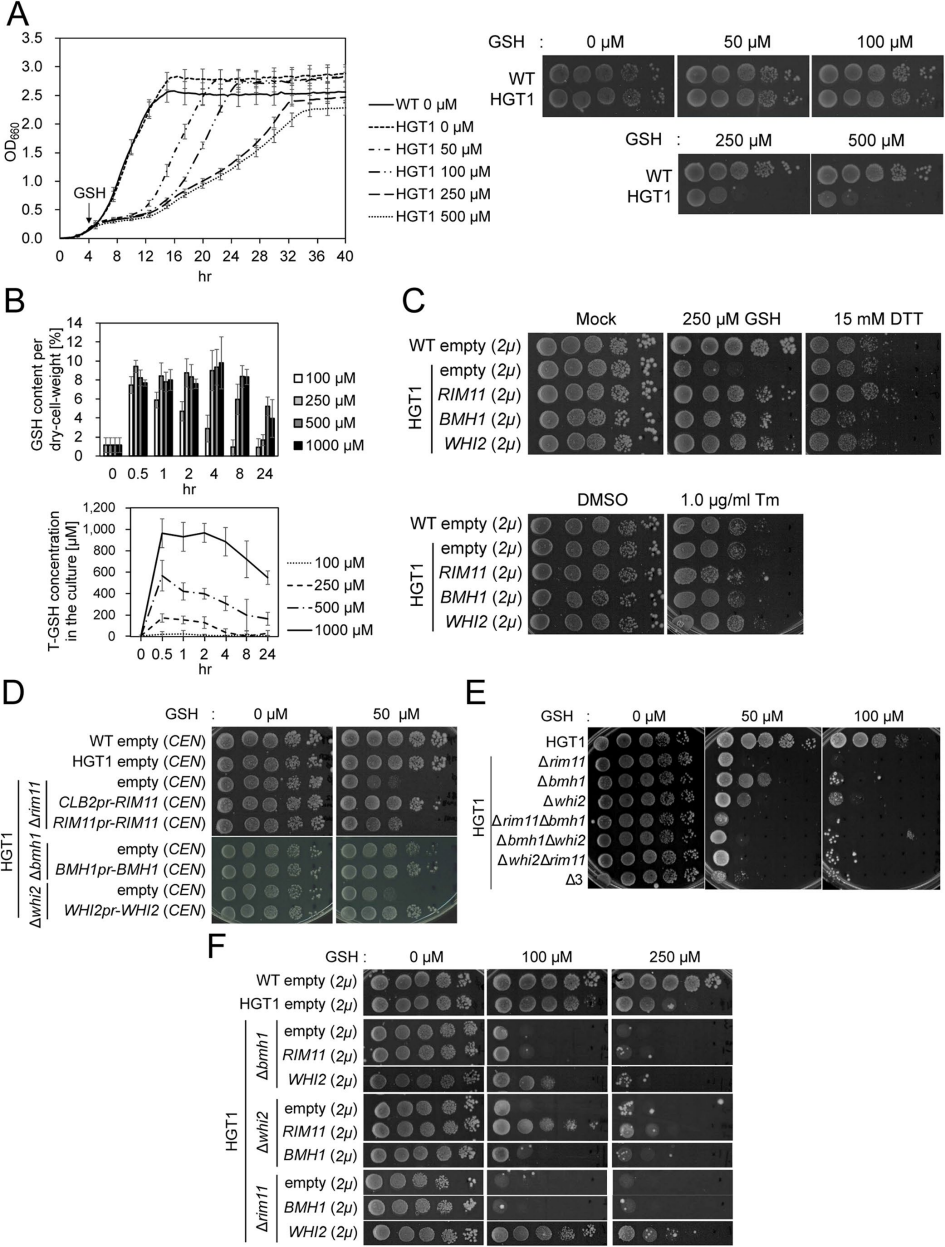
Growth of BY4741 wild-type (WT), *HGT1*-overexpressing strain (HGT1 strain, Hgt1 is a plasma membrane-localized GSH transporter), and its derivative mutants (in HGT1 genetic background) under GSH and/or ER stress. (A) Comparison of the growth phenotype of WT and HGT1 strain cultured in SC (SD supplemented with adenine, uracil, histidine, and tryptophan) at 30°C under various intensities of GSH stress (left, growth experiment; right, spot test). In the left panel, overnight yeast cell culture was diluted with fresh SC to OD_600_ = 0.1 and aerobically shaken for 4 h at 30°C. Then 50–500 µM GSH was added and OD_600_ was monitored over time. Graphs and error bars represent the mean and the SD from two independent experiments (*n* = 4), respectively. In the spot test (right panel), overnight yeast cell culture was adjusted to an OD_600_ of 2.5 with distilled water, and 10-fold serially diluted cell suspensions were prepared. A 10 µl volume of each suspension was spotted on SC agar plate containing 50–500 µM GSH. More than two independent experiments were performed for each condition, and a representative image is shown. Spot assays in Figure 1 were repeated more than twice and the results were reproducible. (B) Intracellular GSH (upper panel) and total GSH (T-GSH = GSH + oxidized glutathione; GSSG; lower panel) content per dry cell weight (%) at various time points in the culture supernatant obtained from HGT1 strain cultures treated with various concentrations of GSH. The concentration of T-GSH and GSSG were measured as described in *Materials and Methods*, and that of GSH was calculated by subtracting the GSSG concentration from that of T-GSH. The bar/line graph and error bars represent mean and SD from three independent experiments (*n* = 6), respectively. (C) Isolation of the multicopy suppressors of the growth defect induced by GSH stress. Tenfold serial dilutions of the cell cultures (OD_600_ of 2.5) in SC-ura were spotted onto SC-ura agar plates with or without GSH, DTT, or Tm, and incubated at 30°C. (D) A spot test was used to observe the growth of the single-deletion strains carrying the suppressor genes on the *CEN* plasmid on SC-ura with or without GSH. Two independent experiments were performed and a representative image is shown. (E) A spot test was used to observe the growth of the double- or triple-gene mutants of HGT1 strains on SC plates with or without GSH. (F) Strains in which each of the three suppressor genes is deleted were transformed with a multicopy plasmid carrying the other suppressor, and their growth on SC-ura plates with or without GSH were tested using a spot assay. Two independent tests were performed and a representative image is shown.

First, the conditions for induction of GSH stress were examined in these strains. The results showed that cell growth was inhibited in a GSH concentration-dependent manner (Figure 1A), and the growth patterns at 250 and 500 μM were similar in both liquid culture and spotting assays. Intracellular GSH content plateaued (8–9% per dry cell weight) at the 4-h time point in the growth medium containing more than 250 µM GSH, while the remaining GSH was detected in parallel in the medium (Figure 1B). These results indicate that the GSH stress levels inside the cells can be controlled by increasing the GSH concentration in the medium to 250 μM. We concluded that the GSH induction system thus established replicated the experimental setup of Kumar *et al*. (2011).

To identify the factors involved in the GSH stress response, we sought to identify high-copy suppressor genes that rescue the GSH-mediated growth inhibition. The HGT strain was transformed with a high-copy number yeast genomic library (Kosodo *et al*., 2001) and 256 colonies that grew faster than the vector control on medium containing 250 μM GSH were isolated. Subsequent analyses identified three different inserts with rescue activity (Supplemental Table 1). By subcloning the individual genes found in the fragments, *RIM11*, *BMH1*, and *WHI2* were found to rescue the growth defects on a high-copy plasmid (Table 1; Figure 1C). High-copy introduction of *BMH2*, a *BMH1* homologue, also rescued the growth defects caused by GSH addition (Supplemental Figure S1C). The protein encoded by *RIM11* (regulator of inducer of meiosis) plays essential roles in the transition from mitosis to meiosis (Rubin-Bejerano *et al*., 2004). Proteins encoded by *BMH1* and *BMH2* belong to the 14-3-3 family and play redundant roles in ribosome biogenesis or catabolite repression (Trembley *et al.*, 2014). The protein encoded by *WHI2* stops cell division when cells enter the stationary phase upon nutrient deprivation (Sudbery *et al*., 1980; Teng *et al*., 2018). Deleting *RIM11*, *BMH1*, or *WHI2* rendered the HGT1 strain sensitive to GSH stress. Introduction of *BMH1* and *WHI2* on a low-copy plasmid rescued the growth retardation phenotypes of the corresponding deletion mutants (Figure 1D). The ∆*rim11* HGT1 strain showed slightly higher sensitivity to GSH than the Δ*whi2* HGT1 strain, whereas the Δ*bmh1* HGT1 strain was less sensitive than the other two strains. Although the introduction of *RIM11* under the control of its own promoter failed to rescue the growth phenotype of the Δ*rim11* HGT strain, when *RIM11* was placed under the *CLB2* promoter, which is expressed only when cells are grown in rich medium and is shut off during meiosis, the growth inhibition was partially alleviated (Figure 1D). Although we also tested the long *RIM11* 5′-region as a promoter, no increase in growth phenotype rescue was observed (unpublished data). It is possible that other elements present on the original plasmid obtained by the high-copy suppressor screening were needed for sufficient expression of the *RIM11* gene. Alternatively, as expression of *RIM11* increases in the stationary phase (Gasch *et al*., 2000), *RIM11* may be insufficiently expressed by its own promoter under the conditions shown in Figure 1D. These results indicated that *RIM11*, *BMH1*, and *WHI2* have essential functions in the GSH stress response. We further examined the GSH stress sensitivity of double or triple mutants of these three genes (Figure 1E). The Δ*bmh1*Δ*whi2* and Δ*rim11*Δ*bmh1* HGT1 strains showed augmented GSH stress sensitivity compared with their single-deletion mutants, as shown in Figure 1E. Furthermore, *RIM11* overexpression did not rescue the GSH sensitivity of the Δ*bmh1* HGT1 strain, and *BMH1* overexpression did not rescue the GSH sensitivity of Δ*rim11* HGT1 (Figure 1F). These results suggest that Bmh1 may function in a different pathway than Rim11. Overexpression of *RIM11* partially rescued the GSH sensitivity of the Δ*whi2* HGT1 strain. Also, overexpression of *WHI2* rescued the GSH sensitivity of the Δ*rim11* HGT1 strain (Figure 1F). These results suggest that Rim11 and Whi2 may function in the similar pathways. The Δ*whi2*Δ*rim11* HGT1 strain showed similar levels of sensitivity to Δ*rim11* HGT1 strain. In contrast, the Δ*whi2*Δ*rim11* HGT1 strain was more sensitive to GSH than the Δ*whi2* HGT1 strain (Figure 1E). These results may suggest Rim11 has more important role than Whi2 in response to GSH stress.

**TABLE 1:** Multicopy suppressor genes that derepressing the growth defect phenotype induced by GSH stress in *S. cerevisiae*, and their functions as described in the *Saccharomyces* Genome Database (www.yeastgenome.org/).

Chromosome	Gene	Function
Chr V	*BMH1*	14-3-3 protein, major isoform
Chr XIII	*RIM11*	Protein kinase
Chr XV	*WHI2*	Negative regulator of TORC1 in response to limiting leucine

We next investigated whether the introduction of the suppressors on a high-copy plasmid would affect the growth of yeast on medium containing dithiothreitol (DTT) or tunicamycin (Tm), both of which induce ER stress similarly to GSH. As shown in Figure 1C, no apparent increase in the tolerance of these strains to DTT or Tm was observed. An increase in the copy number of *ERO1*, which encodes an ER-localized thiol oxidase, has been reported to confer tolerance to DTT-induced ER stress in yeast (Frand and Kaiser, 1998). However, overexpression of *ERO1* failed to confer GSH stress tolerance to the yeast cells (Supplemental Figure S1D). These results suggest that the three suppressors may participate in the GSH stress response by a mechanism distinct from the known mechanisms used to alleviate ER stress. This finding contrasts with the fact that Ire1 functions in coping with both GSH and ER stress (Kumar *et al*., 2011).

As phosphatases and kinases perform important functions in relaying the signals from the inside or outside of the cells to the transcription factors in the nucleus (Mace *et al*., 2020), we sought to understand the role of the Rim11 protein, a kinase involved in coping with nutrient depletion, in the GSH stress-response pathway.

### Rim11 and Mrk1 function to cope with GSH stress

Rim11, which was identified as a high-copy suppressor of growth defects caused by GSH addition, belongs to the glycogen synthase kinase-3β family. Mammalian glycogen synthase kinase-3 (mGSK-3) regulates metabolism, cell division, and cell-fate determination by phosphorylating a wide variety of sequences in its substrates. GSK-3α is ubiquitously expressed in cells and organs. In contrast, the expression of GSK-3β is limited to certain types of cells or at specific timing, such as early meiosis (Guo *et al*., 2003). *S*. *cerevisiae* contains four mGSK-3 orthologues, *MCK1*, *MRK1*, *YGK3*, and *RIM11*, which constitute the GSK-3β family in yeast (yGSK-3β). The percentage identity match of the Rim11 amino acid sequence with those of Mrk1, Mck1, Ygk3, and mouse GSK-3β is 62.3, 41.1, 36.9, and 53.3%, respectively (Supplemental Figure S2), and the proteins have overlapping functions (Neigeborn and Mitchell, 1991; Kassir *et al*., 2006; Zhou *et al*., 2017; Huang *et al*., 2019). This prompted us to investigate whether orthologues other than *RIM11* are involved in the GSH stress response.

**FIGURE 2:**
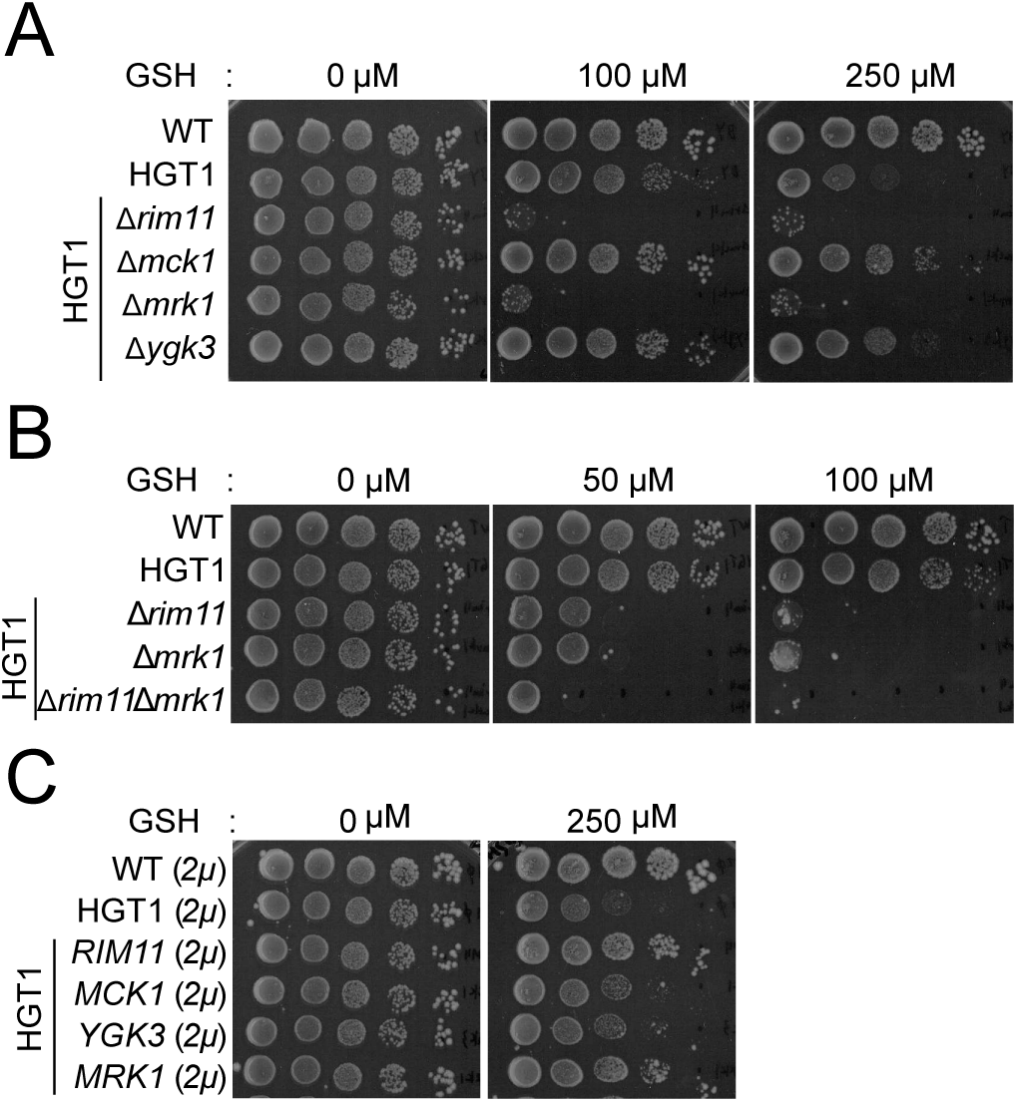
Spot assay of the yeast strains with deletion or overexpression of yeast glycogen synthase kinase-3β (yGSK-3β) genes. (A) HGT1 strains with yGSK-3β single gene deletion. (B) HGT1 strains with single-deletion of *RIM11* or *MRK1,* and double deletion of both *RIM11* and *MRK1*. (C) HGT1 strains carrying the yGSK-3β genes on a multicopy plasmid (*2μ URA3*). Spot assays were performed as described in Figure 1. Two independent experiments were performed and a representative image is shown.

A high-copy *HGT1* expression cassette was introduced into the Δ*mck1*, Δ*ygk3*, and Δ*mrk1* strains, and their GSH sensitivities were tested. Disruption of the *MRK1* gene increased the GSH sensitivity of the cells to a level as high as that of Δ*rim11* HGT1 strain. In contrast, deletion of *MCK1* or *YGK3* did not alter GSH sensitivity (Figure 2A). In addition, Δ*rim11*Δ*mrk1* HGT1 strain showed higher GSH sensitivity than the single-deletion mutant (Figure 2B). When *MCK1*, *YGK3*, or *MRK1* was overexpressed in the HGT strain, only *MRK1* overexpression conferred GSH stress tolerance (Figure 2C), consistent with the highest amino acid identity between Rim11 and Mrk1 among the yGSK-3β family proteins. These results suggest that Rim11 and Mrk1 have overlapping functions in certain steps of the signal-response pathway.

### Unlike Ire1, Rim11 is not involved in the general ER stress response

**FIGURE 3:**
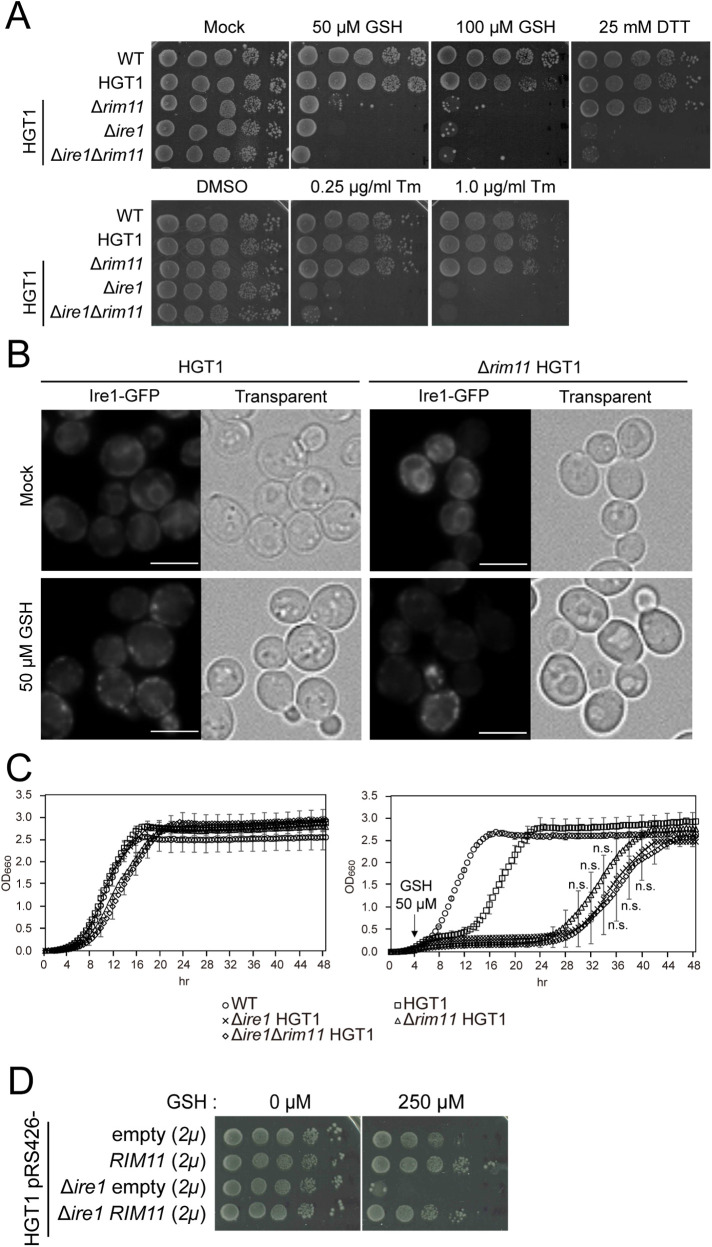
Analyses of the roles of Rim11 and Ire1 in GSH or ER stress response. (A) Spot assays of the Δ*rim11*, Δ*ire1* or Δ*rim11*Δ*ire1* strains (in the HGT1 background) under GSH or ER stress. Spot assays were performed as described in Figure 1. Two independent tests were performed and a representative image is shown. (B) Microscopic images of the intracellular localization of Ire1-GFP in HGT1 or Δ*rim11* HGT1 strains with or without GSH stress. Cells grown in SC at 30°C to an OD_600_ of 1 were treated with or without 50 µM GSH, cultured for another 2 h, and observed without fixation. A total of three to five fields were examined for each biological sample. Scale bar, 5 µm. (C) Time course of the growth of Δ*rim11*, Δ*ire1* or Δ*rim11*Δ*ire1* mutants (in the HGT1 background) in the absence (left panel) or presence (right panel) of GSH stress. Cells were diluted to an OD_600_ of 0.1 in SC and shaken for 4 h at 30°C. Then 50 µM GSH was added and the cells were incubated further for 4 h under the same condition. Dots and error bars represent mean and SD from two independent experiments (*n* = 4), respectively. Significant differences are analyzed using two-tailed Welch’s *t* test. n.s., not significant. (D) *RIM11* was overexpressed on a high-copy plasmid in the Δ*ire1* HGT1 strain and sensitivity to GSH stress was tested. Two independent experiments were performed and a representative image is shown.

GSH stress induces ER stress, which is alleviated by Ire1-dependent unfolded protein response (UPR). The Δ*ire1* HGT1 strain displayed high sensitivity to GSH stress (Supplemental Figure S1B; Kumar *et al*., 2011). In contrast, although Δ*rim11* HGT1 strain was also highly sensitive to GSH stress, analyses of cells overexpressing *RIM11* suggested that Rim11-mediated mechanism involved in coping with GSH stress was distinct from that used to alleviate the effects of general ER stress (Figure 1C). Therefore, we investigated the functional relationships between *RIM11* and *IRE1* in greater detail. First, we assessed the sensitivity of Δ*rim11* HGT1 strain to ER stress. As shown in Figure 3A, Δ*rim11* HGT1 strain did not display sensitivity to DTT or Tm, which is consistent with the results with cells carrying *RIM11* on a high-copy plasmid (Figure 1C). This result strongly suggests that Rim11 functions independently of the Ire1-dependent UPR. Next, we examined the intracellular localization of Ire1-GFP using fluorescence microscopy in the presence or absence of GSH stress (Figure 3B, Supplemental Figure S3, A and B). In the absence of ER stress, Ire1-GFP exhibited a typical double-ring ER distribution pattern. In contrast, accumulation of unfolded proteins during ER stress led to Ire1-GFP dimerization, which was observed as intracellular Ire1 foci (Aragón *et al*., 2009). In addition, induction of denatured carboxypeptidase Y following GSH stress has been reported to induce UPR through the transcriptional regulator Hac1 that is activated upon intron removal (Kumar *et al*., 2011). In our experiments, in HGT1 strain (used as a parental strain), Ire1-GFP exhibited an ER double-ring distribution pattern in the absence of GSH stress and formed clusters in the presence of relatively weak GSH stress (50 μM; Figure 3B; Supplemental Figure S3A). A similar change in localization was observed for Ire1-GFP in the Δ*rim11* HGT1 strain in the presence of 50 μM GSH, which corresponds to strong GSH stress in the Δ*rim11* HGT1 strain (Figure 3B, Supplemental Figure S3B). These results suggest that loss or impaired stress response mechanisms other than UPR may be the cause for the lethality of the Δ*rim11* HGT1 strain when subjected to weak GSH stress (50 μM). Moreover, quantitative assessment of growth revealed no significant differences in the level of GSH sensitivity among Δ*ire1*Δ*rim11* HGT1, Δ*ire1* HGT1, and Δ*rim11* HGT1 strains (Figure 3, A and C). *RIM11* was overexpressed on a high-copy plasmid in the Δ*ire1* HGT1 strain and its sensitivity to GSH stress was tested. We found that *IRE1* is not necessary for *RIM11* to function as a multicopy suppressor of GSH stress (Figure 3D). These results suggest that both Rim11 and Ire1 have important functions in GSH stress response, but that Rim11 is unlikely to be involved in general ER stress response.

### Kinase activity of Rim11 is required for mediating GSH stress tolerance

Next, we investigated the mechanism by which Rim11 mediates GSH stress tolerance. Given that Rim11 was identified as a high-copy suppressor, we used strains overexpressing *RIM11* (in addition to strains with wild-type backgrounds that express *RIM11*) from the chromosomal copy in the following experiments.

**FIGURE 4:**
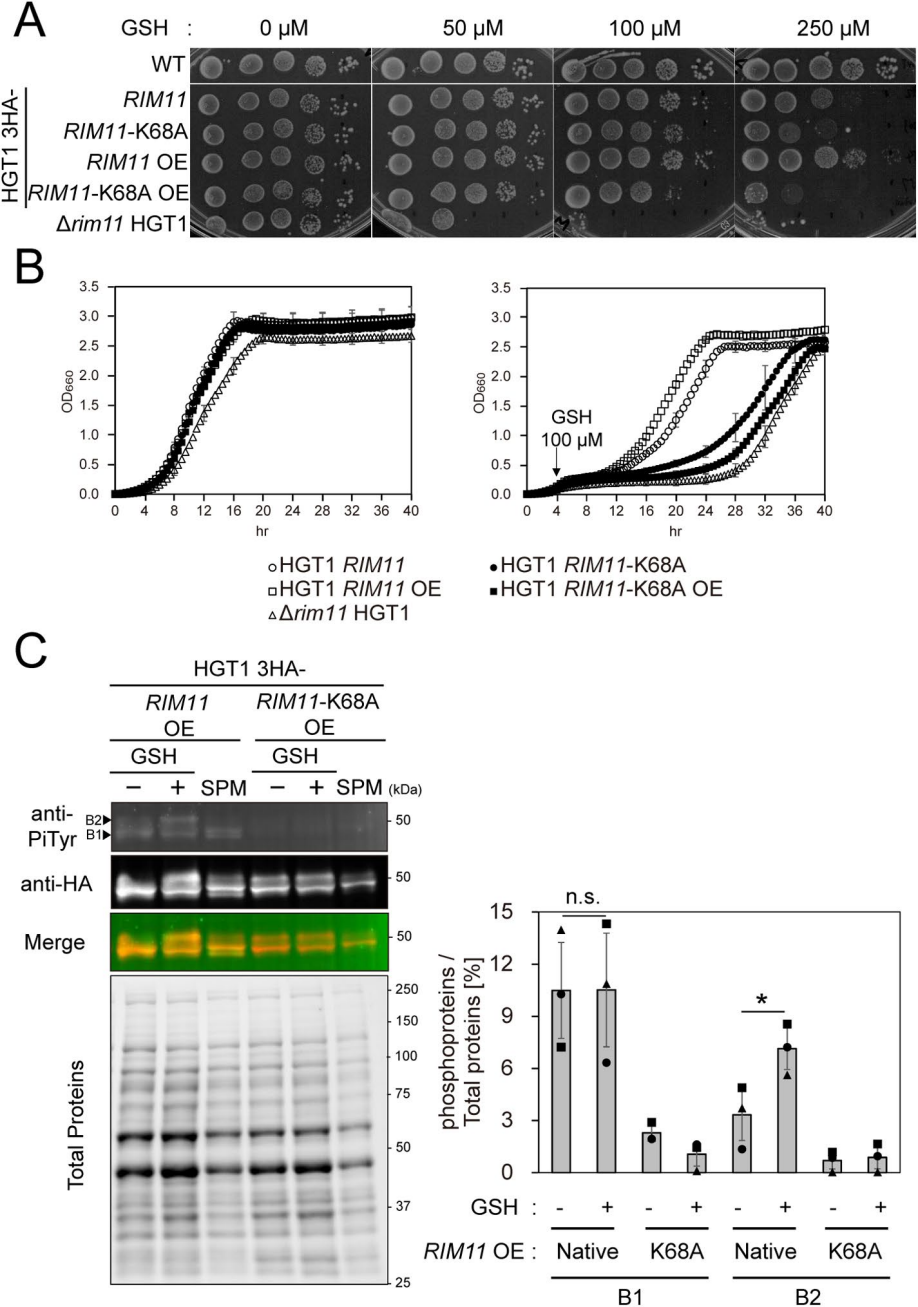
Function of the kinase-dead mutant of Rim11. (A) Spot assay of the cells overexpressing *RIM11* or kinase-dead *RIM11*^K68A^. Spot assays were performed as described in Figure 1. Two independent tests were performed and a representative image is shown. (B) Time course of the growth of the indicated mutants under non-stress (mock, left panel) or GSH stress (100 µM GSH, right panel). Cells were diluted to OD_600_ = 0.1 in SC, shaken for 4 h at 30°C, and then 100 µM GSH was added. Dots and error bars represent the mean and SD from two independent replicates (*n* = 4), respectively. (C) Immunoblotting of 3HA-Rim11 and 3HA-Rim11 K68A in the cell lysates of HGT1 *RIM11* OE and HGT1 *RIM11*-K68A OE strains (-, without GSH stress; +, with 250 µM GSH stress; SPM, potassium acetate medium). Each mutant growing in exponential phase (OD_600_ of 1) in SC at 30°C was exposed to GSH stress (250 µM GSH) or nutrient starvation (replacing SC with SPM), and subsequently shaken for 2 h at 30°C. Cells were harvested by centrifugation at RT, fixed by mixing with TCA/ethanol, disrupted in urea-containing buffer by bead beating, and then cell lysates were prepared for western blotting. Cell lysates containing 20 µg of total protein was applied to each lane of TGX Stain-Free gels (Bio-Rad). Anti-PiTyr (anti-phosphotyrosine mAb) and anti-HA (anti-hemagglutinin rAb) were used as primary antibodies, and Alexa Fluor488 conjugated anti-mAb goat antibody and Alexa Fluor plus800 conjugated anti-rAb goat antibody were used as secondary antibodies. Band B1 and B2 correspond to phosphorylated Rim11 and Rim11 with enhanced phosphorylation, respectively. One of the three independent experimental results is shown as the representative blot. Signal intensities were detected using ChemiDoc MP Imaging System (Bio-Rad). The intensities of the bands detected using anti-PiTyr mAb were normalized to the amount of total protein. Quantification results of three independent experiments (*n* = 3) are plotted as graphs (right panel). Asterisk indicates significant differences analyzed using two-tailed Welch’s *t* test, * *p* < 0.05, and n.s., not significant.

The *RIM11* promoter was replaced with the *TDH3* promoter, and the coding sequence of 3 × hemagglutinin (HA) was N-terminally added to the *RIM11* open reading frame (ORF), to generate the HGT1 3HA-*RIM11* OE strain. We confirmed that the addition of the triple HA-tag to the N-terminus of Rim11 did not have any detectable effect on its function (Supplemental Figure S4A; Zhan *et al*., 2000). HGT1 3HA-*RIM11* strain (control) was also generated, in which 3HA-*RIM11* was expressed from its own promoter. Assessment of the growth of these strains in the presence of GSH showed that the HGT1 3HA-*RIM11* OE strain grew faster than the HGT1 3HA-*RIM11* strain even in the presence of high concentrations of GSH (250 μM), which suggested that the addition of the HA-tag at the N-terminus of Rim11 did not significantly affect its function (Figure 4A).

Next, we investigated whether the kinase activity of Rim11 is required for mediating GSH stress tolerance or whether an increase in Rim11 level was sufficient. Glucose starvation enhances the phosphorylation of Rim11 at Tyr199 and activates it via the Ras/cAMP/PKA signal transduction pathway (Zhan *et al*., 2000; Rubin-Bejerano *et al*., 2004). A K68A amino acid substitution in Rim11 was reported to inactivate its kinase activity, and a diploid strain harboring this substitution in both *RIM11* alleles failed to enter meiosis or form spores (Zhan *et al*., 2000). To investigate whether the kinase activity of Rim11 is involved in GSH stress response, strains with the amino acid substitution HGT1 *THD3pr*-3HA*-RIM11-*K68A (HGT1 3HA-*RIM11*-K68A OE) and HGT1 *RIM11pr-*3HA*-RIM11-*K68A (HGT1 3HA-*RIM11*-K68A) were created. In the strains that overexpress *RIM11*, the amounts and intracellular localization of the 3HA-Rim11 protein were examined before the analyses. Western blotting of the cell lysates showed that the amount of Rim11 protein overexpressed in 3HA-Rim11 and 3HA-Rim11 K68A were very similar, and greater than that produced with the *RIM11* promoter (Supplemental Figure S4B)*.* Moreover, indirect immunofluorescence microscopy revealed that 3HA-Rim11 and 3HA-Rim11 K68A were mainly localized in the nucleus (Supplemental Figure S4C). Thus, we confirmed that overexpression of the Rim11 protein, whose kinase activity was lost following the introduction of an amino acid substitution, led to no detectable change in its protein amount or intracellular localization. The HGT1 3HA-*RIM11*-K68A OE and HGT1 3HA-*RIM11*-K68A strains displayed high sensitivity to GSH stress, indicating that the kinase activity of Rim11 plays an important role in the GSH stress response (Figure 4, A and B). However, growth in the spot assay in the presence of GSH at 50 or 100 μM (Figure 4A), and time course of the growth pattern (Figure 4B) revealed that expression of the kinase-dead Rim11 mutant endowed the HGT1 strain with partial resistance to GSH stress. This result suggests that the amount of Rim11 protein may contribute to GSH stress tolerance.

Rim11 was reported to be constitutively Tyr phosphorylated on the Tyr-199 residue by its autophosphorylation activity, and a shift in the carbon source from glucose to acetate enhanced phosphorylation at Tyr199 (Zhan *et al*., 2000). We tested whether a similar response would be observed in the case of GSH stress. Whole cell lysates of HGT1 3HA-*RIM11* OE and HGT1 3HA-*RIM11*-K68A OE strains grown under GSH stress conditions (250 µM) for 2 h or whole cell lysates of the same strains shifted from SC medium to sporulation medium (SPM) for 2 h as a positive control were subjected to fluorescent Western blot analysis (Figure 4C). In the HGT1 3HA-*RIM11* OE strain, protein bands detected with anti-HA and anti-PiTyr antibodies overlapped with each other, confirming that the phosphorylated bands (Figure 4C, indicated by arrows B1 and B2) were derived from the 3HA-Rim11 proteins. The bands B1 and B2 are hereafter used to refer to phosphorylated Rim11 and Rim11 with enhanced phosphorylation, respectively. Furthermore, in the HGT1 3HA-*RIM11* OE strain, the signal intensity of band B2 normalized to that of the total protein amount (loaded in each gel lane) increased markedly from 3.3 (in the absence of GSH stress) to 7.1 (in the presence of GSH stress), indicating that the ratio of Rim11 with enhanced phosphorylation increased in response to GSH stress. As another distinct band was observed between B1 and B2 in the sample shifted to SPM, it is likely that the degree of phosphorylation and/or amino acids on which phosphorylation occurs are different under GSH stress and glucose starvation conditions. In contrast, in the 3HA-*RIM11*-K68A OE strain, no signals were detected at the B1 or B2 band positions when immunoblotted with the anti-PiTyr antibody, which supports the loss of autophosphorylation activity. These results suggest that autophosphorylation of Rim11 is enhanced under GSH stress conditions, similar to glucose depletion. Therefore, this raises the possibility that yeast cells cope with GSH stress via a pathway that overlaps with the glucose starvation response pathway, at least in some of the steps that involve Rim11 phosphorylation.

### Transcriptomic analysis of Rim11-dependent differentially expressed genes (DEGs) in cells under GSH stress

To further understand the GSH stress response in yeast, we performed transcriptome analysis of the various strains that were generated in this study. The transcriptomic changes induced by GSH stress were analyzed in the strains. GSH (250 μM) was added to the growth medium when the cells were in early log-phase and incubated with shaking for 2 h. Total RNA was extracted, and mRNA expression was analyzed by RNA sequencing (RNA-seq). Three biologically independent samples in each dataset were grouped into the same clusters following cluster analysis based on read count data from all samples (Supplemental Figure S5A) and the average values of the three samples were used for subsequent analyses. DEGs were determined as having false discovery rate (FDR) < 0.05 and log_10_CPM (counts per million) ≥ 1.

**FIGURE 5:**
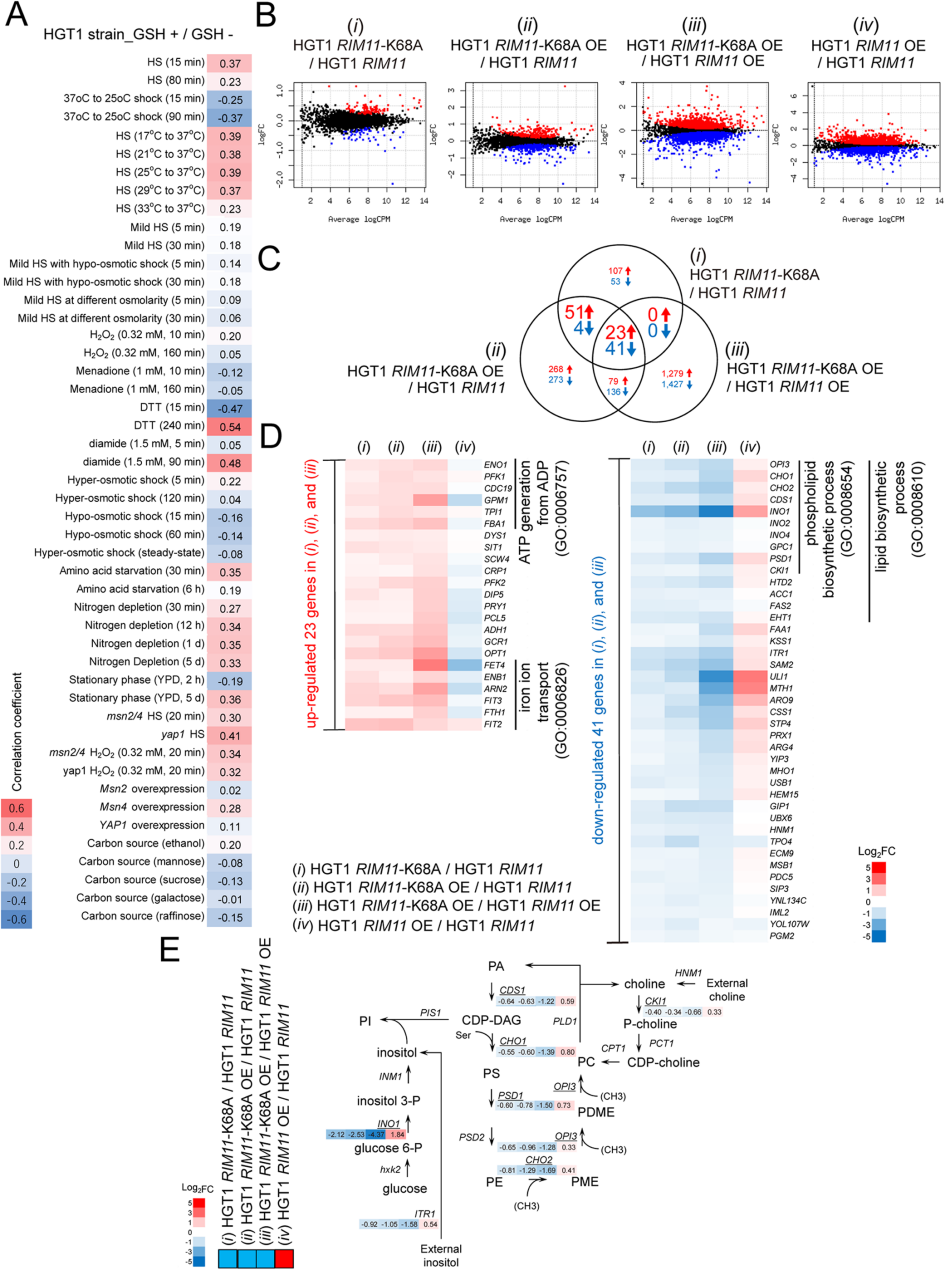
RNA-seq analyses of HGT1 strain and HGT1 derivatives with kinase-dead *RIM11*. (A) Correlation of differentially expressed genes (DEGs) found in HGT1 strain under 250 µM GSH stress condition (cutoff; FDR < 0.05, and log_10_CPM ≥ 1, note that absolute value of log_2_fc ≥ 1 is not necessarily met) with those in *S. cerevisiae* under various stress conditions (compared with nonstress conditions; Gasch *et al*., 2000; Tsai *et al*., 2019). HS, heat shock. Pearson correlation scores are indicated in colors (red, positive correlation; blue, negative correlation). (B) MD-plots showing the log-fold change and average abundance of each gene in the following four sample sets (here “strain” is omitted). *i*) HGT1 *RIM11*-K68A/HGT1 *RIM11*, *ii*) HGT1 *RIM11*-K68A OE/HGT1 *RIM11*, *iii*) HGT1 *RIM11*-K68A OE/HGT1 *RIM11* OE, and *iv*) HGT1 *RIM11* OE/HGT1 *RIM11*. All datasets were obtained under GSH stress (250 µM). log_10_CPM ≥ 1 and FDR < 0.05 served as cutoff values for selection of the genes. Up- or downregulated genes that satisfy the cutoff values are represented as red or blue dots, respectively, and the black dots represent DEGs with no significant difference. (C) A Venn diagram of the three sample sets, *i*), *ii*), and *iii*). The numbers in red indicate upregulated genes and those in blue indicate genes, respectively. (D) The 64 genes listed were identified as genes with FDR < 0.05 and log_10_CPM ≥ 1 in all three datasets *i*), *ii*), and *iii*). Changes in the expression of these 64 genes in dataset *iv*) were also displayed. As the log2-fold change in the transcriptional levels of most of the DEGs identified in *i*) and *ii*) were < 1 or > –1, DEGs in these data sets were detected without using a log_2_fc threshold. The color scale, log_2_fc, shows the magnitude of expression of the selected genes. GO enrichment analysis (www.yeastgenome.org/goTermFinder) was performed on the selected 23 up- and 41 downregulated DEGs and the enriched biological processes (GO terms) are shown. (E) Schematic diagram of the phospholipid biosynthesis pathway in *S. cerevisiae*. Expression levels of DEGs that belong to phospholipid biosynthetic process in (D) are shown. The color scale shows the magnitude of expression, log_2_fc, of the selected genes.

We examined the DEGs in the HGT1 strain and found that the transcript levels of 475 genes were upregulated (log_2_fc ≥ 1), whereas those of 496 genes were downregulated (log_2_fc ≤ –1) in the presence of GSH (Supplemental Figure S5B). These DEGs were analyzed by Gene Ontology (GO) enrichment analysis using GO::TermFinder from the *Saccharomyces* Genome Database, and the resulting top 20 terms are listed in Supplemental Figure S5, C and D. In the upregulated group of genes, the terms associated with iron ion homeostasis, cell wall biogenesis, and spore wall biogenesis were markedly enriched. In contrast, in the downregulated group of genes, terms associated with purine nucleobase biosynthesis, biosynthesis of IMP (the final product of the purine nucleobase synthesis pathway), and ribosome biogenesis were enriched. In addition, we compared DEGs that satisfied FDR < 0.05, but did not necessarily satisfy log_2_fc ≥ 1 and log_2_fc ≤ –1, with those identified and reported in yeast under various other stress conditions (Gasch *et al*., 2000; Tsai *et al*., 2019; Figure 5A). We found that the DEGs in GSH stress condition showed positive correlation (R^2^ > 0.4) with those observed under other stress conditions, such as heat, reducing conditions, oxidizing conditions, or nitrogen starvation, or those detected in cells in the stationary phase. The results of this analysis suggest that GSH stress is not simply a form of ER stress, but may contain multiple stress factors that are shared by other stresses. In addition, the highest correlation with DEGs found in DTT (240 min), which is a reductive stress, and the marked increase in genes associated with iron ion homeostasis (Supplemental Figure S5C) are similar to the results reported previously (Kumar *et al*., 2011). The RNA-seq data obtained in this study were thus confirmed to adequately reflect the GSH stress response at the transcriptomic level.

We next sought to identify DEGs dependent on Rim11 protein abundance and kinase activity under GSH stress conditions to investigate which signal transduction or metabolic pathways are preferentially activated when cells respond to GSH stress. In the four sample sets: *i*) HGT1 *RIM11*-K68A/HGT1 *RIM11*, *ii*) HGT1 *RIM11*-K68A OE/HGT1 *RIM11*, *iii*) HGT1 *RIM11*-K68A OE/HGT1 *RIM11* OE, and *iv*) HGT1 *RIM11* OE / HGT1 *RIM11* (each “strain” is omitted), DEGs that satisfied FDR < 0.05 and log_10_CPM ≥ 1 were extracted (Figure 5B, red: upregulated; blue: downregulated). As log2-fold changes in the transcriptional levels of most DEGs identified in *i*) HGT1 *RIM11*-K68A / HGT1 *RIM11* and *ii*) HGT1 *RIM11*-K68A OE / HGT1 *RIM11* were < 1 or > –1, DEGs in these datasets were detected without using a log_2_fc threshold. We identified 23 commonly upregulated and 41 commonly downregulated DEGs in kinase-dead datasets *i*), *ii*), and *iii*); Figure 5C). Changes in the expression of these 64 genes in all datasets, including dataset *iv*), in which the expression changes following overexpression of the kinase-active form (wild-type) of Rim11 were measured, are displayed in the form of a heatmap (Figure 5D). GO analysis of these genes using parameters of *p* value < 0.01 and FDR < 0.05 revealed that many of the upregulated genes were categorized into ATP generation from ADP (GO:0006757) and iron ion transport (GO:0006826) processes, with the former being enriched 65.3-fold (26.1%/0.40%) and the latter 43.5-fold (26.1%/0.60%) relative to the genome-wide frequencies, respectively (Figure 5D; Table 2; Supplemental Figure S5E, upper panel).

**TABLE 2:** Selected differentially expressed genes (DEGs) identified by GO enrichment analysis of the following RNA-seq sample sets: (*i* ) HGT1 *RIM11*-K68A/HGT1 *RIM11*, (*ii* ) HGT1 *RIM11*-K68A OE/HGT1 *RIM11*, (*iii* ) HGT1 *RIM11*-K68A OE/HGT1 *RIM11* OE and (*iv*) HGT1 *RIM11* OE/HGT1 *RIM11* (here “strain” is omitted). Function of DEGs as described in the *Saccharomyces* Genome Database. Upper table; ATP generation from ADP (GO:0006757), middle table; iron ion homeostasis (GO:0055072), and lower table; phospholipid biosynthetic process (GO:0008654) and lipid biosynthetic process (GO:0008610).

Symbol	Name	Function	Fold change			
			(*i*)	(*ii*)	(*iii*)	(*iv*)
*ENO1*	YGR254W	Enolase I, a phosphopyruvate hydratase; catalyzes conversion of 2-phosphoglycerate to phosphoenolpyruvate during glycolysis and the reverse reaction during gluconeogenesis; expression repressed in response to glucose; protein abundance increases in response to DNA replication stress; N-terminally propionylated in vivo	0.50	0.62	0.80	–0.18
*PFK1*	*YGR240C*	Alpha subunit of heterooctameric phosphofructokinase; involved in glycolysis, indispensable for anaerobic growth, activated by fructose-2,6-bisphosphate and AMP, mutation inhibits glucose induction of cell cycle-related genes	0.35	0.72	0.56	0.16
*CDC19*	*YAL038W*	Pyruvate kinase; functions as a homotetramer in glycolysis to convert phosphoenolpyruvate to pyruvate, the input for aerobic (TCA cycle) or anaerobic (glucose fermentation) respiration; regulated via allosteric activation by fructose bisphosphate	0.48	0.65	0.70	–0.04
*GPM1*	*YKL152C*	Tetrameric phosphoglycerate mutase; mediates the conversion of 3-phosphoglycerate to 2-phosphoglycerate during glycolysis and the reverse reaction during gluconeogenesis	0.61	0.65	1.89	–1.23
*TPI1*	*YDR050C*	Triose phosphate isomerase, abundant glycolytic enzyme; mRNA half-life is regulated by iron availability; transcription is controlled by activators Reb1p, Gcr1p, and Rap1p through binding sites in the 5′ noncoding region; inhibition of Tpi1p activity by PEP (phosphoenolpyruvate) stimulates redox metabolism in respiring cells	0.36	0.72	0.57	0.15
*FBA1*	*YKL060C*	Fructose 1,6-bisphosphate aldolase; required for glycolysis and gluconeogenesis; catalyzes conversion of fructose 1,6 bisphosphate to glyceraldehyde-3-P and dihydroxyacetone-P; localizes to mitochondrial outer surface upon oxidative stress; N-terminally propionylated in vivo	0.69	0.91	1.21	–0.30
*ARN2*	*YHL047C*	Transporter; member of the ARN family of transporters that specifically recognize siderophore-iron chelates; responsible for uptake of iron bound to the siderophore triacetylfusarinine C	0.83	0.68	1.82	–1.14
*ENB1*	*YOL158C*	Ferric enterobactin transmembrane transporter; expressed under conditions of iron deprivation	0.64	0.31	0.83	–0.53
*FET4*	*YMR319C*	Low-affinity Fe (II) transporter of the plasma membrane	0.42	0.40	2.60	–2.20
*FIT2*	*YOR382W*	Mannoprotein that is incorporated into the cell wall; incorporated via a glycosylphosphatidylinositol (GPI) anchor; involved in the retention of siderophore-iron in the cell wall	1.16	1.25	0.78	0.48
*FIT3*	*YOR383C*	Mannoprotein that is incorporated into the cell wall; incorporated via a GPI anchor; involved in the retention of siderophore-iron in the cell wall	0.84	1.11	1.33	–0.22
*FTH1*	*YBR207W*	Putative high-affinity iron-transporter; involved in transport of intravacuolar stores of iron; forms complex with Fet5p; expression is regulated by iron	0.31	0.31	0.92	–0.61
*INO1*	*YJL153C*	Inositol-3-phosphate synthase; involved in synthesis of inositol phosphates and inositol-containing phospholipids	–2.12	–2.53	–4.37	1.84
*OPI3*	*YJR073C*	Methylene-fatty-acyl-phospholipid synthase; catalyzes the last two steps in PC biosynthesis	–0.65	–0.96	–1.28	0.33
*CHO1*	*YER026C*	Phosphatidylserine synthase; functions in phospholipid biosynthesis; transcriptionally repressed by myo-inositol and choline	–0.55	–0.60	–1.39	0.80
*CHO2*	*YGR157W*	Phosphatidylethanolamine methyltransferase (PEMT)	–0.81	–1.29	–1.69	0.41
*PSD1*	*YNL169C*	Phosphatidylserine decarboxylase of the mitochondrial inner membrane; converts phosphatidylserine to phosphatidylethanolamine	–0.60	–0.78	–1.50	0.73
*CKI1*	*YLR133W*	Choline kinase; catalyzes the first step in PC synthesis via the CDP-choline (Kennedy pathway)	–0.40	–0.34	–0.66	0.33
*CDS1*	*YBR029C*	Phosphatidate cytidylyltransferase (CDP-diglyceride synthetase); an enzyme that catalyzes that conversion of CTP + phosphate into diphosphate + CDP-diaclglyerol, a critical step in the synthesis of all major yeast phospholipids	–0.64	–0.63	–1.22	0.59
*GPC1*	*YGR149W*	Glycerophosphocholine acyltransferase (GPCAT); involved in PC synthesis; uses acyl-CoA to acylate glycero-3-phosphocholine to yield lyso-PC; also catalyzes acylation of glycerophosphoethanolamine with acyl-CoA	–0.30	–0.43	–0.35	–0.08
*INO2*	*YDR123C*	Transcription factor; component of the heteromeric Ino2p/Ino4p basic helix-loop-helix transcription activator that binds inositol/choline-responsive elements (ICREs), required for derepression of phospholipid biosynthetic genes in response to inositol depletion; involved in diauxic shift	–0.36	–0.55	–0.45	–0.10
*INO4*	*YOL108C*	Transcription factor involved in phospholipid synthesis; required for derepression of inositol-choline-regulated genes involved in phospholipid synthesis; forms a complex, with Ino2p, that binds the inositol-choline-responsive element through a basic helix-loop-helix domain	–0.38	–0.45	–0.41	–0.04
*HTD2*	*YHR067W*	Mitochondrial 3-hydroxyacyl-thioester dehydratase; involved in fatty acid biosynthesis, required for respiratory growth and for normal mitochondrial morphology	–0.62	–0.77	–1.05	0.29
*ACC1*	*YNR016C*	Acetyl-CoA carboxylase, biotin containing enzyme; catalyzes carboxylation of cytosolic acetyl-CoA to form malonyl-CoA and regulates histone acetylation by regulating the availability of acetyl-CoA; rate-limiting step for de novo biosynthesis of long-chain fatty acids; translational regulation in response to nutrients and cell cycle stage depends on its upstream ORF	–0.49	–0.42	–0.46	0.04
*FAS2*	*YPL231W*	Alpha subunit of fatty acid synthetase; complex catalyzes the synthesis of long-chain saturated fatty acids; contains the acyl-carrier protein domain and beta-ketoacyl reductase, beta-ketoacyl synthase and self-pantetheinylation activities	–0.55	–0.55	–0.40	–0.15
*EHT1*	*YBR177C*	Octanoyl-CoA:ethanol acyltransferase; also functions as thioesterase; plays a minor role in medium-chain fatty acid ethyl ester biosynthesis; localizes to lipid particles and the mitochondrial outer membrane	–0.69	–0.73	–0.91	0.18

In contrast, we performed GO-based analysis for 41 commonly downregulated DEGs in kinase-dead datasets *i*), *ii*), and *iii*) (Figure 5C) under the same conditions mentioned above. Among these, the 14 downregulated genes were categorized as being involved in the lipid biosynthetic process (GO:0008610) (Figure 5D). Among them, ten genes including *INO1, CHO2*, *PSD1*, *CKI1*, *CHO1*, *CDS1*, *GPC1*, *INO2*, *INO4*, and *OPI3* were enriched 14-fold (24.4%/1.7%) in the phospholipid biosynthetic process (GO:0008654) (Figure 5D; Table 2; Supplemental Figure S5E, lower panel). Interestingly, these genes are phospholipid biosynthesis genes, whose transcription is regulated by Opi1 (a transcriptional repressor) and Ino2-Ino4 (a basic helix-loop-helix transcriptional activator complex) (Hickman *et al*., 2011; Ye *et al*., 2013). In addition, as shown in Figure 5D, the upregulated or downregulated genes in sample sets *i*)–*iii*) were mostly inversely regulated in sample set *iv*). In addition, the magnitude of increase or decrease in expression of the genes correlated more with the kinase activity (compare sample sets *iii*) with *iv*)) than with the abundance of Rim11 protein (compare sample sets *ii*) with *iv*)), which was most apparent in *INO1* (the gene encoding inositol 3-phosphate synthase), *ULI1* (the gene encoding a protein of unknown function), and *MTH1* (the gene encoding the negative regulator of the glucose-sensing signal transduction pathway). The expression levels of phospholipid biosynthesis genes superimposed with those in the yeast lipid biosynthetic pathway indicate that the transcription of genes encoding a series of enzymes that function in the pathway for the synthesis of phosphatidylcholine from CDP-diacylglycerol (CDP-DAG) are coordinately regulated (Figure 5E) (Carman and Han, 2009; Hickman *et al*., 2011; Klug and Daum, 2014). These results suggest that GSH stress enhances the kinase activity of Rim11, which in turn activates lipid biosynthesis by upregulating the transcription of genes in the biosynthetic pathway.

### GSH stress response partly overlaps with nutrient starvation response pathway

Rim11 is a kinase that responds to starvation. The results described in the previous section show that the transcriptome response pattern of cells under GSH stress correlates with those associated with nitrogen starvation or stationary phase. Therefore, we hypothesized that GSH stress involves several factors that induce response to multiple stresses, including nutrient starvation.

**FIGURE 6:**
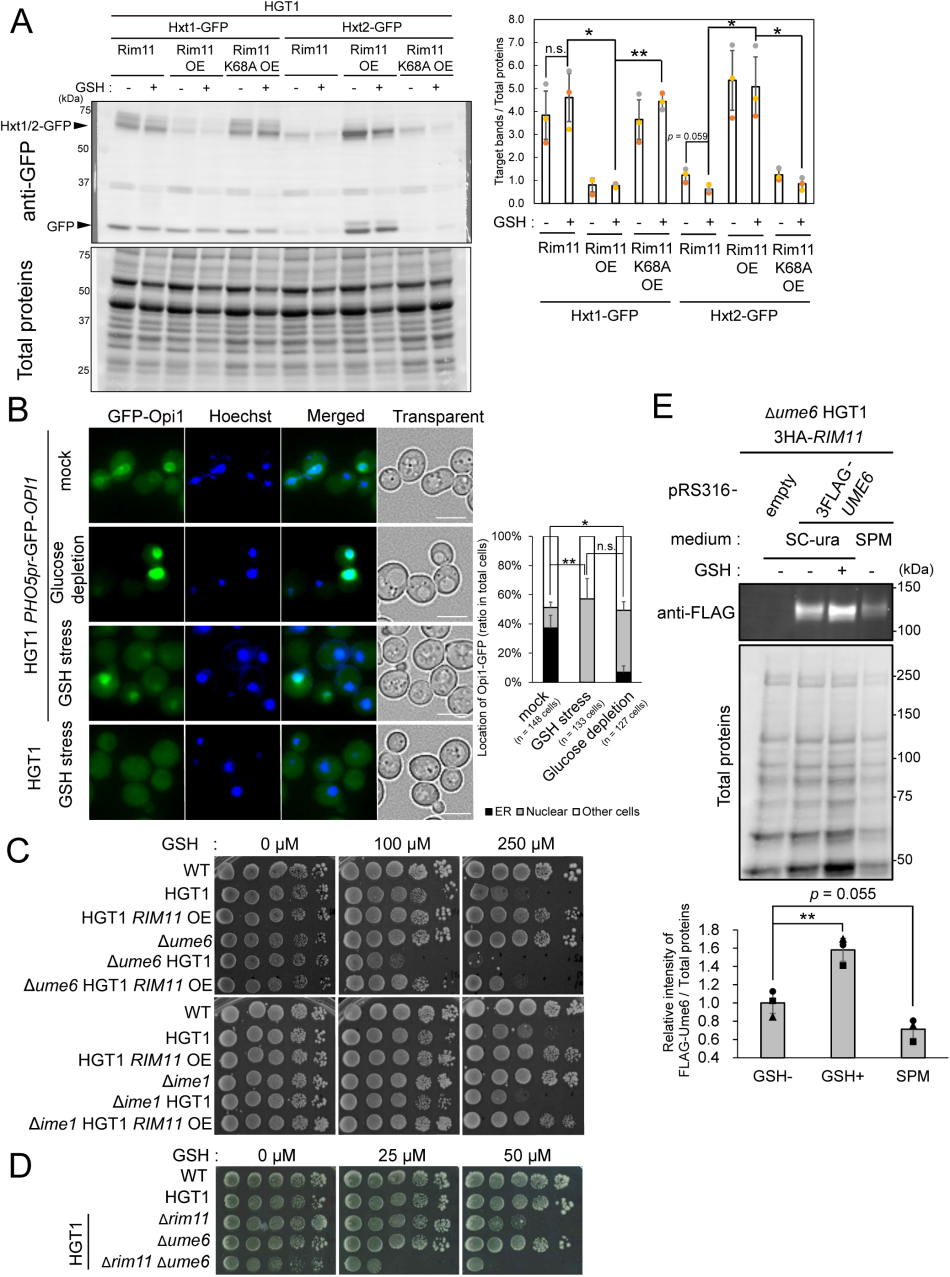
Biochemical analyses of Hxt1, Hxt2, Opi1, Ime1, and Ume6 during GSH stress conditions. (A) Quantitation of the intracellular levels of Hxt1-GFP or Hxt2-GFP in the absence or presence of GSH stress by immunoblotting of HGT1 *RIM11*, HGT1 *RIM11* OE, and HGT1 *RIM11*-K68A OE. Each strain was grown in SC at 30°C until OD_600_ = 1 and GSH (250 µM) was added. The strains were then incubated further for 2 h, harvested cells were fixed in TCA/ethanol and lysates were prepared as described in the *Materials and Methods* section. Anti-GFP rAb and Alexa Fluor plus800-conjugated anti-rAb goat antibody were used as primary and secondary antibodies, respectively. A representative image (left) is shown. Signal intensities were measured using the ChemiDoc MP Imaging System (Bio-Rad). Relative intensities shown in the bar graph (right) were calculated from two independent experiments (*n* = 3). Asterisk indicates significant differences analyzed using two-tailed Welch’s *t* test, * *p* < 0.05, ** *p* < 0.01. (B) Microscopic images of intracellular localization of GFP-Opi1 in HGT1 GFP-*OPI1* strain under non-stress condition (mock), in the presence of 250 µM GSH (GSH stress), or in SC without glucose (glucose depletion). Cells grown in SC at 30°C until log-phase (OD_600_ = 1) were treated with 250 µM GSH or switched to SC minus glucose medium and shaken at 30°C for another 2 h. Cells were collected by centrifugation and stained with Hoechst 33342 solution at 30°C for 15 min. To determine the level of background fluorescence or autofluorescence originating from the live cells, images of HGT1 strain under GSH stress condition were also acquired. A total of three to five fields were examined for each biological sample. Scale bar, 5 µm. The ratio of cells with GFP-Opi1 in the ER or nucleus were counted and graphed (right). (C) Spot tests of Δ*ume6* and Δ*ime1* strains. Tenfold serial dilutions of the indicated strains cultured in SC were spotted onto SC agar plates containing 0, 100, and 250 µM GSH. Two independent tests were performed, and a representative image is shown. (D) Spot test of Δ*rim11*Δ*ume6* HGT1 strain. Tenfold dilutions of overnight culture grown in SC were spotted onto SC agar plates in the absence or presence of GSH and incubated at 30°C. Two independent tests were performed and a representative image is shown (E) Quantitation of the intracellular levels of 3 × Flag-Ume6 in the absence or presence of GSH stress by immunoblotting of Δ*ume6* HGT1 3HA-*RIM11*/pRS316 (an empty vector, *CEN URA3*) and Δ*ume6* HGT1 3HA-*RIM11*/pRS316-3FLAG-*UME6*. Each strain was grown in SC-ura at 30°C until OD_600_ = 1. Then GSH (250 µM) was added or SC-ura was replaced with SPM, and the cells were incubated further for 2 h. To prepare cell lysates, cells were fixed in TCA, washed once with 70% (vol/vol) ethanol, and disrupted in a buffer containing 6 M urea using bead-beater (see *Materials and Methods* in detail). Anti-FLAG mAb and Alexa Fluor 488 conjugated anti-mAb goat antibody were used as primary and secondary antibodies, respectively. A representative image (top) is shown. Signal intensities were measured using ChemiDoc MP Imaging System (Bio-Rad). Relative intensities shown in the bar graph were calculated from three independent experiments (*n* = 3). Asterisk indicates significant differences analyzed using two-tailed Welch’s *t* test, ** *p* < 0.01.

To test this hypothesis, we first examined the regulatory factor involved in glucose sensing. During *RIM11* OE-mediated GSH stress tolerance, a signal induced by the overexpressed *RIM11* greatly increased the expression of *MTH1* (log_2_fc of 2.42) via unknown mechanisms. In contrast, overexpression of *RIM11-*K68A substantially decreased (log_2_fc of –3.09) *MTH1* expression (Figure 5D; Supplemental Table 2, see sample set *iii*) and *iv*)). Furthermore, the expression of the low-affinity glucose transporter *HXT1* was repressed (log_2_fc of –3.12), whereas that of the high-affinity glucose transporter *HXT2* was markedly upregulated (log_2_fc of 3.80; Supplemental Table 2, see sample set *iv*)). Considering that Mth1 also represses the expression of *HXT1*, a gene encoding a low-affinity glucose transporter, in the presence of high glucose (Roy *et al*., 2013), we hypothesized that a relationship may exist between the kinase activity of Rim11 and the dynamics of hexose transporters. To analyze the dynamics of glucose transporters in the GSH stress response, we created yeast strains producing glucose transporters to which GFP was fused c-terminally. *RIM11* overexpression decreased the protein abundance of Hxt1, a low-affinity glucose transporter, and increased that of Hxt2, a high-affinity glucose transporter (Figure 6A). We hypothesized that this may be one of the mechanisms by which *RIM11* alleviates GSH stress as a high-copy suppressor. We observed a tendency for Hxt1-GFP to increase and Hxt2-GFP to decrease (*p* = 0.059, *n* = 3) upon GSH treatment (Figure 6A). Moreover, microscopic measurements revealed a significant increase of Hxt1-GFP signal at the plasma membrane upon GSH treatment (Supplemental Figure S6, A and B). Therefore, these transcriptional and proteomic changes were presumed to alleviate the stress caused by low glucose.

We next examined the intracellular localization of the transcriptional repressor Opi1. In vegetative cells, Opi1 is tethered in the ER/nuclear membranes via interaction with phosphatidic acid and the membrane-spanning protein Scs2. In the presence of inositol or in glucose-starved cells, Opi1 is released from the ER/nuclear membranes and translocates into the nucleus, where it represses the expression of phospholipid biosynthetic genes by directly binding to Ino2 (Hickman *et al*., 2011; Ye *et al*., 2013). We introduced a GFP-*OPI1* expression construct into the HGT1 strain and examined its localization in the presence or absence of GSH stress, or in cells shifted to glucose-depleted medium for 2 h (Figure 6B). GFP-Opi1 was found to exhibit the typical double-ring ER distribution pattern in cells grown in SC medium containing glucose. In contrast, in a medium lacking glucose, a strong fluorescent signal indicating nuclear localization was also detected, as reported previously (Young *et al*., 2010). When GSH was added (250 μM) to the medium, translocation of GFP-Opi1 into the nucleus was observed. The ratio of cells with GFP-Opi1 in the ER or nucleus were counted and graphed. Nuclear localization of GFP-Opi1 was significantly increased not only by glucose starvation, but also upon GSH treatment (Figure 6B; Supplemental Figure S6C), which suggests that Opi1 may be involved in the GSH stress response and represses the transcription of phospholipid biosynthetic genes.

Next, we examined the role of Rim11 protein in the transcription of phospholipid biosynthetic genes. We first tested the sensitivities of the deletion mutants of *UME6* (which encodes a promoter DNA-binding transcriptional repressor) and *IME1* (which encodes a dominant transcriptional activator of meiosis initiation), both of which are known substrates of Rim11 kinase, to GSH stress (Malathi *et al*., 1997; Xiao and Mitchell, 2000; Figure 6C; Supplemental Figure S6D). Compared to the growth of the HGT1 strain, the growth of Δ*ime1* HGT1 strain was largely unaffected, whereas that of Δ*ume6* HGT1 strain showed high sensitivity to GSH stress, suggesting that Ume6 functions in GSH stress response (Figure 6C; Supplemental Figure S6D). Furthermore, the degree GSH stress tolerance of the Δ*ime1* HGT1 *RIM11* OE strain was very similar to that of the HGT1 *RIM11* OE strain, further supporting that Ime1 is unlikely to play a major role in GSH stress tolerance (Figure 6C). In contrast, *RIM11* overexpression in the Δ*ume6* HGT1 strain only partially rescued the growth defect of the Δ*ume6* HGT1 strain under GSH stress (Figure 6C). Furthermore, the Δ*rim11*Δ*ume6* HGT1 strain showed synthetic GSH sensitivity (Figure 6D), suggesting that Rim11 and Ume6 function in different pathways to cope with GSH stress.

Finally, we investigated the behavior of the Ume6 protein in response to GSH stress. Some of the mechanisms by which diploid yeast cells transit from mitosis to early meiosis upon nutrient starvation are as follows: 1) Rim11, which is activated by a glucose starvation signal, promotes the formation of the Ime1-Ume6 complex by phosphorylating both Ime1 and Ume6 proteins. 2) The Ime1-Ume6 complex is completely degraded by the APC/C^Cdc20^ ubiquitin ligase (Mallory *et al*., 2007; Cooper and Strich, 2011). 3) The recruitment of the Rpd3-Sin3 histone deacetylase (HDAC) complex, which is bound by Ume6 during mitosis, to the URS1 sequences in the promoters of early meiosis-specific genes is repressed, leading to their transcriptional activation (Rubin-Bejerano *et al*., 1996; Malathi *et al*., 1997; Malathi *et al*., 1999). Based on these and our findings (Figure 6C; Supplemental Figure S6D), we focused on Ume6, a known substrate of Rim11 kinase. To detect Ume6 protein by immunoblotting, a 3 × Flag sequence was added at the N-terminus of *UME6*, and its expression was driven by its own promoter. The resulting low-copy plasmid, pRS316-3FLAG-*UME6* (*CEN URA3*) was introduced into the Δ*ume6* HGT1 3HA-*RIM11* strain. As previously reported (Xiao and Mitchell, 2000), shifting the vegetative cells into SPM led to a smear with decreased electrophoretic mobility in the blot corresponding to Ume6 band, confirming that our experiments were successful (Figure 6E). When the same strains in the early log phase were placed under GSH stress, the band intensity of 3FLAG-Ume6 was markedly increased (Figure 6E). This result indicates that GSH stress may increase Ume6 abundance and suggests a model in which yeast cells cope with GSH stress by regulating the expression of downstream genes via signal transduction through Ume6.

### GSH treatment and overexpression of *RIM11* impact a phospholipid composition

**FIGURE 7:**
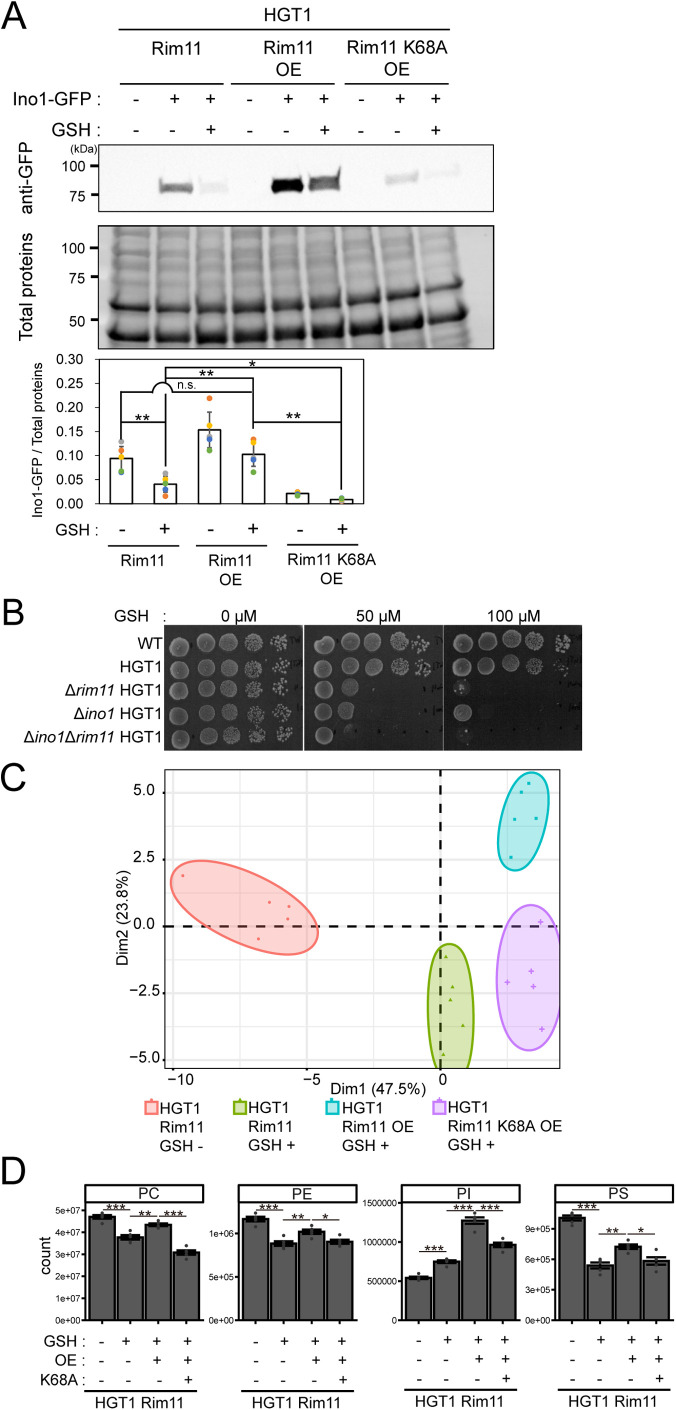
Lipid association analysis under GSH-induced stress. (A) Quantification of Ino1-GFP with or without GSH treatment in *RIM11*, *RIM11* OE, and *RIM11*-K68A OE (all in HGT1 as genetic background). Cells were cultured in SC at 30°C up to an OD_600_ of 1.0. Subsequently, GSH stress was induced by addition of 250 µM GSH, and then the cells were incubated further for 2 h. Anti-GFP rAb, Alexa Fluor800 plus-conjugated anti-rAb goat antibody, and the amount of total protein was used for the quantitative western blotting method. Results of three independent experiments (*n* = 5) are plotted as bar graphs, and a representative blot is shown. Asterisk indicates significant differences analyzed using two-tailed Welch’s *t* test, * *p* < 0.05, ** *p* < 0.01, and n.s., not significant. (B) Spot test of the Δ*ino1* strain. Tenfold dilutions of overnight culture grown in SC were spotted onto SC agar plates in the absence or presence of GSH and incubated at 30°C. Two independent tests were performed, and a representative image is shown. (C) Principal component analysis for lipid extracted from HGT1 Rim11, HGT1 Rim11 OE, and HGT1 Rim11 K68A OE strains (*n* = 5 per sample). Cells precultured in SC were diluted in fresh SC at OD_600_ = 0.25 and cultivated at 30°C. Then, GSH stress was induced by treatment with 250 µM GSH per OD_600_ = 1.0, and the cells were further grown for 2 h. A total of 3.0 × 10^9^ cells (20 OD_600_ units) were harvested. Lipids were extracted from these harvested cells using the BUME method (Löfgren *et al*., 2012). Lipidomic analysis was performed as described previously (Nakao *et al*., 2019; Watanabe *et al*., 2022). (D) Intracellular contents of PC, PE, PI, and PS in HGT1 3HA-Rim11, HGT1 3HA-Rim11 OE, and HGT1 3HA-Rim11 K68A OE strains (*n* = 5 per sample) in the absence or presence of 250 µM GSH treatment.

Among the genes whose expression changed distinctively between conditions (Figure 5D), we chose *INO1* and protein level was analyzed. The Ino1-GFP expression construct was integrated into the chromosome of the HGT1 strain and Ino1-GFP was detected by Western blotting with anti-GFP antibody (Figure 7A). GSH treatment of the HGT1 *RIM11* strain significantly decreased the protein level of Ino1-GFP, which was consistent with the change in transcription of *INO1* revealed by RNA-seq analysis (Supplemental Figure S5B). Also, in the presence of GSH, overexpression of *RIM11* increased the protein level of Ino1-GFP by 2.5-fold, and overexpression of *RIM11 K68A* decreased it by 11-fold. This result is consistent with the RNA-seq data of *INO1* in sample set *iv*) and *iii*), in which log_2_fc of HGT1 *RIM11* OE/HGT1 *RIM11* and of HGT1 *RIM11*-K68A OE/HGT1 *RIM11* OE were 1.84 and –4.37, respectively (Figure 5D; Table 2).

Ume6 functions as a DNA-binding transcriptional repressor that downregulates the transcription of the *INO1* gene by binding to its upstream repression sequence (URS1, 5′-AGCCGCCGA-3′), and on the other hand it upregulates the transcription of *CHO1/2* and *OPI3* genes by indirectly increasing transcription of the *INO2* gene (Jackson and Lopes, 1996; Elkhaimi *et al*., 2000; Kaadige and Lopes, 2003; Henry *et al*., 2014). Although Ume6 is a Rim11 kinase substrate, it is not known whether Rim11 functions in the transcriptional regulation of phospholipid biosynthetic genes including *INO1*. Therefore, we assessed the functional relationship between Rim11 and Ino1 in GSH stress response. As shown in Figure 7B, the Δ*ino1* HGT1 strain showed sensitivity to GSH stress; the Δ*rim11*Δ*ino1* HGT double deletion mutant displayed more severe growth defects compared with each single-deletion mutant grown in medium containing GSH (50 μM). This suggests that *RIM11* and *INO1* co­operatively mediate GSH stress response in a nonepistatic manner. Considering that *INO1* transcription was greatly altered in response to the loss of Rim11 kinase activity (Figure 5D), transcriptional regulators that control *INO1* transcription in response to the signals from Rim11 likely exist.

Subsequently, lipidomic analysis was then performed to determine whether the observed transcriptional changes in the phospholipid biosynthetic genes lead to the changes in the phospholipid composition in the cell. A principal component analysis was performed to understand the overall pattern of phospholipid variability in terms of each biological condition (Figure 7C). The results clustered into four groups, suggesting that lipid compositions differed among four sample sets. When we looked at alterations in the phospholipid composition more closely (Figure 7D), we found that PC, PE, and PS contents in HGT1 Rim11 strain were significantly reduced by GSH treatment, which was consistent with decreases in the mRNA levels of *OPI3*, *PSD1*, and *CHO1* as revealed by the RNA-seq analysis (Supplemental Figure S5B). In contrast, PI content was increased upon GSH treatment. Moreover, *RIM11* overexpression suppressed the reduction of PC, PE, and PS caused by GSH treatment and greatly increased the amount of PI. In contrast, overexpression of *RIM11-*K68A decreased suppressing effects on the reduction of these lipids. These results show that changes in the Rim11 kinase activity leads to changes in lipid compositions.

## DISCUSSION

### GSH stress as mixed stress

We speculate that GSH stress is sensed by *S*. *cerevisiae* as mixed stress, including oxidative, glucose starvation, reductive (Figure 3, A and C; Supplemental Figure S1B; Kumar *et al*., 2011; Ponsero *et al*., 2017), and iron-deficiency stresses (Kumar *et al*., 2011). First, we discuss the relationship between redox and iron stresses with GSH stress.

In mammalian cells, reductive stress induced by a redox imbalance can cause oxidative stress (Zhang *et al*., 2012; Korge *et al*., 2015). This paradoxical phenomenon was also observed in our experiment with *S*. *cerevisiae* under various stress conditions (Figure 5A). DEGs detected in GSH stress condition positively correlated with those identified in reductive (240 mM DTT) and oxidative stress (1.5 mM diamide, a thiol oxidizing agent for 90 min) conditions with R^2^ values of 0.54 and 0.48, respectively. This result was consistent with the observed increase in intracellular GSSG levels following induction of GSH stress (500–1000 µM GSH) in the HGT1 strain (Supplemental Figure S1E). However, this correlation was not observed with DEGs identified in H_2_O_2_ or menadione treatment groups, which led us to speculate that the relationship between GSH and oxidative stress may be affected by alterations in the redox balance of thiols. To our knowledge, this is the first study to indicate that reductive stress may trigger oxidative stress in yeast cells.

Next, we discuss the relationship between GSH stress and Fe levels. The importance of GSH in iron metabolism and homeostasis has been reported previously (Kumar *et al*., 2011; Berndt and Lillig, 2017). In our study, RNA-seq analysis revealed that the transcription of genes involved in the maintenance of iron ion homeostasis, especially that of *ARN2* and *FET4* (iron ion transporter genes), were downregulated on Rim11 kinase activity-dependent manner (Figure 5D; Table 2). This suggests that Rim11, activated by high levels of GSH, participates in the maintenance of iron homeostasis via transcriptional regulation of iron-transporter genes.

### Relationship between GSH and glucose starvation-induced stresses

We assume that *RIM11*, *BMH1/2*, and *WHI2*, the multicopy suppressors identified in this study, increase the adaptive tolerance to GSH stress by activating the glucose starvation-stress response pathway.

**FIGURE 8:**
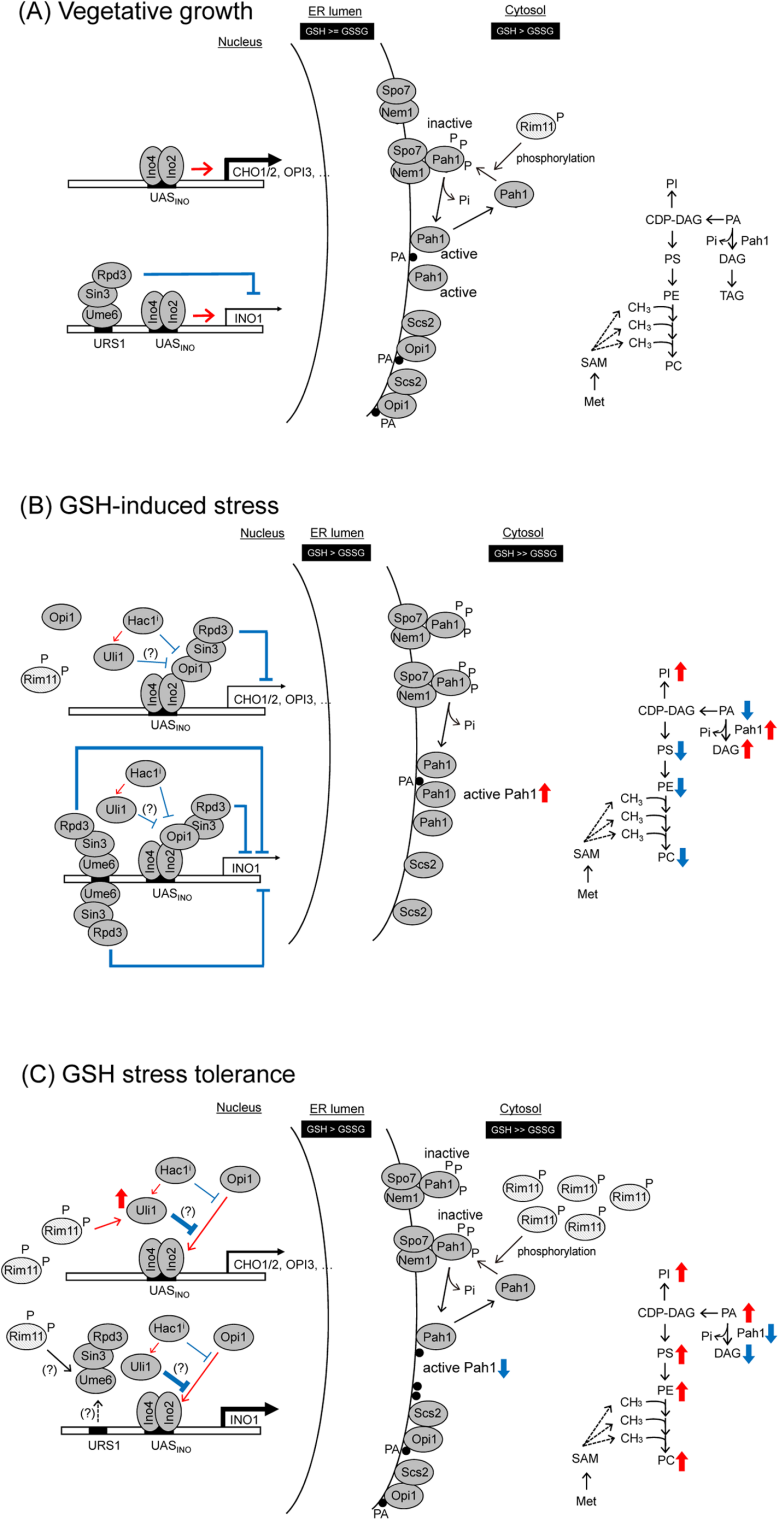
Hypothetical models for the transcriptional regulation of phospholipid biosynthesis genes by Rim11 and other factors in *S. cerevisiae.* (A) During vegetative growth, the Opi1 repressor is tethered to the ER membrane via interactions with phosphatidic acid and Scs2. The transcriptional activator Ino2-Ino4 complex binds to the UAS_INO_ sequence of the phospholipid biosynthetic genes such as *CHO1/2*, *OPI3*, and *INO1* and upregulates their transcription. Ume6 decreases transcription of the phospholipid biosynthetic genes by recruiting Rpd3 and Sin3. A relevant phospholipid biosynthetic pathway is depicted (right). (B) GSH stress is an unfavorable environmental condition for yeast cells. Under GSH stress conditions, Rim11 translocates into the nucleus and phosphorylation of Pah1 by Rim11 is decreased. Then reactivated Pah1 converts PA to DAG, which leads to the reduction of the PA level, and translocation of Opi1 into the nucleus. The Opi1 repressor in the nucleus binds to the Ino2 subunit of the Ino2-Ino4 complex and causes overall downregulation of transcription of the UAS_INO_-containing phospholipid biosynthetic genes. We speculate that an increase in Ume6 abundance may in some ways contribute to transcriptional repression of the phospholipid biosynthetic genes (Figure 6E). Changes in the phospholipid levels are shown in the pathway diagram (Figure 7D). PA and DAG were not measured in our analysis and their changes expected from the Pah1 activity were shown. (C) Overexpressed Rim11 phosphorylates Pah1, thereby dephosphorylating activity of Pah1 is reduced. The proportion of Opi1 anchored on the ER membrane then increases, which probably leads to transcriptional derepression of the phospholipid biosynthetic genes. We showed that maintaining the expression level of *INO1* was required for GSH stress response (Figure 7B). *ULI1*, a gene encoding a protein of unknown function, is transcriptionally upregulated not only by Hac1, but also by Rim11 (Figure 5D; Supplemental Table 2). Hac1 is known to act antagonistically on the Ino2-Ino4 heterodimer (Cox *et al*., 1997), which leads us to believe that Hac1 weakens the transcriptional repression by Opi1. Uli1 may also negatively affect Opi1 function. Changes in the phospholipid levels are shown as in (B). Collectively, our data suggest a possible role for Rim11 in optimally controlling the amount of different phospholipid species cooperatively both in the presence or absence of GSH stress. Scs2, an integral ER membrane protein that regulates phospholipid metabolism; Pah1, Mg^2+^-dependent phosphatidate (PA) phosphatase; Ino2-Ino4, a basic helix-loop-helix transcriptional activator complex; Rpd3-Sin3, HDAC complex which is bound by Ume6 during mitosis; Opi1, a transcriptional repressor; Ume6, encodes a promoter DNA-binding transcriptional repressor; Nem1-Spo7, phosphatase holoenzyme; Hac1, transcriptional regulator that is activated upon intron removal; Uli1, the gene encoding a protein of unknown function. Black circle (filled), phosphatidic acid (PA). UAS_INO_ and URS1, upstream activation and repression sequences, respectively. Dashed lines represent potential interaction. Arrows indicate positive roles and lines ending in bars indicate negative roles.

First, we demonstrated that Rim11 was mainly localized in the nucleus and the GSH stress signal increased the autophosphorylation of Rim11 (Figures 4C; Supplemental Figure S4C). Rim11 function in this signal transduction pathway appeared to be similar to that used in response to glucose starvation, including meiosis and spore formation, which suggests that some parts of the glucose starvation-response pathway may be used to cope with GSH stress (Figure 8). GSH caused a marked decrease in *MTH1* transcription (log_2_fc of –2.14), likely independent of glucose concentration (Supplemental Table 2, see “HGT1 strain GSH +/GSH –”). A mutation in *MTH1* (*HTR1-23*) has been reported to induce abnormal expression of genes encoding glucose transporters. This led to a reduced rate of glucose consumption and roughly 50% decrease in the growth rate of the mutant strain (Özcan *et al.*, 1993). Mth1 also represses the expression of *HXT1* in the presence of high glucose (Roy *et al*., 2013). We speculate that GSH reduces the expression of *MTH1,* which causes yeast cells to preferentially express low-affinity glucose transporters, independent of glucose concentration. Actually, fluorescent intensities of Hxt1-GFP on the plasma membrane was increased under GSH-induced stress environment in HGT1 Rim11 strain (Supplemental Figure S6, A and B). As a result, when the cells consume glucose from the medium containing GSH during proliferation, they are unable to cope with low-glucose conditions, which affect their growth negatively. Glucose starvation by GSH likely triggers the translocation of Opi1 into the nucleus (Figure 6B), where it represses the expression of phospholipid biosynthetic genes that contain UAS_INO_ elements in their promoters. The combined effect of GSH on these pathways may impair yeast cell growth. Therefore, we believe that these transcriptional changes alleviate the stress caused by low-glucose levels and the transcriptional repression by Opi1. Ume6 likely also receives signals from overexpressed Rim11 and relieves the stress caused by GSH via the derepression of UAS-containing genes. The Δ*ume6* HGT1 strain showed high GSH sensitivity (Figure 6C), indicating that the simple loss of depression by Ume6 augments sensitivity. This suggests that transcriptional regulation via a balance between repression and derepression of relevant genes is important for coping with GSH stress.

Whi2 may contribute to GSH stress response via the Ras/cAMP/PKA pathway. Whi2 has been reported to halt the activity of the Ras/cAMP/PKA pathway by transporting the Ras protein to the vacuole, thereby facilitating its degradation. In addition, shutdown of this pathway is essential for cells to display their full stress response capabilities (Reinders *et al*., 1998; Leadsham *et al*., 2009). It is also known that in the presence of glucose, phosphorylation of Rgt1 via the Ras/cAMP/PKA pathway facilitates the release of Ssn6-Tup1, which results in derepression of *HXT* gene expression. Therefore, overproduction of Whi2 may suppress the phosphorylation of Rgt1 and downregulate the expression of *HXT* genes by promoting inactivation of the Ras/cAMP/PKA pathway.

Finally, we assumed that the growth promoting effects of Bmh1 or Bmh2 during catabolite repression may be responsible for coping with GSH stress. When the concentration of extracellular glucose is high, the transcription of genes involved in the metabolism of carbon sources other than glucose is repressed. This is known as glucose repression, and in Δ*bmh1* or Δ*bmh2* cells, glucose repression is partially derepressed (Dombek *et al*., 2004). Although the detailed mechanism remains unknown, Bmh1 OE may increase GSH tolerance by modulating the response to glucose.

### The mechanism of transcriptional regulation of the phospholipid biosynthetic genes by Rim11 during GSH stress response

In this section, we discuss the mechanisms by which yeast cells respond to or show increased tolerance to GSH stress, focusing on the Rim11-dependent transcriptional control of phospholipid synthetic genes. Figure 8A shows the function of Rim11 in the GSH stress response. Khondker *et al*. (2022) reported that Rim11 phosphorylates phosphatidic acid phosphatase (Pah1), and thereby inhibits its phosphatase activity. Pah1 is dephosphorylated by the Nem1-Spo7 complex and is subsequently recruited to the nuclear/ER membranes where it performs its functions (Karanasios *et al*., 2010; Choi *et al.*, 2011). At the membrane, Pah1 dephosphorylates phosphatidic acid and converts it into DAG. This regulates the localization and function of Opi1, which is tethered to the ER membrane via interaction with PA and the ER membrane protein, Scs2 (Loewen *et al*., 2004). Therefore, it is likely that the kinase activity of Rim11 and the function of Pah1 play an important role in the recovery of phospholipid biosynthetic gene expression levels in the GSH stress response. First, we examined yeast cells under vegetative growth and GSH stress conditions (Figure 8, A and B). The HGT1 strain, in which Rim11 is not overproduced, grew at a reduced rate in the presence of GSH (>50 μM concentration; Figure 1A), indicating that this environmental condition is not favorable for yeast growth. Under this stress condition, Opi1 translocated from the ER to the nucleus (Figure 6B). Opi1 is known to suppress the expression of phospholipid synthesis genes, including *INO1*, *CDS1*, *PSD1*, *CHO1*, *CHO2*, *OPI3*, and *CKI1* (Hickman *et al*., 2011; Ye *et al*., 2013). Similarly, in this study, under GSH stress conditions, the expression of *INO1*, *CDS1*, *PSD1*, and *CHO1* was downregulated (log_2_fc < –1), and that of *CHO2*, *OPI3*, and *CKI1* was also weakly repressed (Supplemental Figure S5B). At the protein level, Ino1-GFP was significantly decreased upon GSH treatment (Figure 7A). Moreover, PC, PE, and PS contents were decreased upon GSH treatment (Figure 7D). We hypothesize that Rim11 translocates into the nucleus following GSH treatment (Figure 4C; Supplemental Figure S4C), and phosphorylation of Pah1 by Rim11 is decreased. We predict that reactivated Pah1 converts PA to DAG, which leads to the reduction of PA levels and translocation of Opi1 into the nucleus, ultimately leading to repression of the transcription of the phospholipid biosynthetic gene (Figure 8B). Furthermore, we found that GSH stress may increase the abundance of the Ume6 protein (Figure 6E). Although the Ume6 protein downregulates the expression of *INO1* and upregulates the expression of *CHO1*, *CHO2*, and *OPI3* (Elkhaimi *et al*., 2000), the addition of GSH lowers the overall expression of phospholipid biosynthetic genes.

Next, we discuss the mechanism by which overexpression of *RIM11* confers resistance to GSH stress (Figure 8C). Overexpression of *RIM11* restored the growth of yeast cells on GSH media in a manner mainly dependent on its kinase activity (Figures 1C, 2C, and 4A). The RNA-seq data demonstrated that overexpression of *RIM11* induced the overall expression of phospholipid biosynthetic genes, especially that *INO1* gene (Figure 5, D and E). The amount of Ino1-GFP was consistent with the changes in the *INO1* mRNA level (Figure 7A). Moreover, lipidomic analyses revealed that overexpression of *RIM11* increased the overall lipid contents in the cell (Figure 7D). Therefore, we hypothesized that derepression of the decreased biosynthetic activity of phospholipids by Rim11, and the increased expression of the *INO1* gene, may contribute to enhanced tolerance of the cells to GSH. Specifically, overexpressed Rim11 phosphorylates Pah1, thereby inhibiting the dephosphorylating activity of Pah1. We hypothesize that the proportion of Opi1, which is anchored on the ER membrane, then increases, causing transcriptional derepression of the phospholipid biosynthetic genes.

Based on these findings and assumptions, we hypothesized that deletion of the *UME6* gene may decrease the sensitivity to GSH stress caused by the transcriptional derepression of the *INO1* gene. In contrast, the Δ*ume6* HGT1 strain showed increased sensitivity to GSH stress (Figure 6C; Supplemental Figure S6D). In addition, unlike *RIM11*, multicopy introduction of the *INO1* gene did not confer GSH stress resistance (Supplemental Figure S7). Furthermore, Δ*ino1* HGT1 strain showed increased sensitivity to GSH stress, which suggests that maintenance of the transcriptional level of *INO1* gene is necessary to cope with GSH stress. Based on these results, we propose that overexpression of *RIM11* not only increases the transcription of *INO1*, but also induces or activates other factors necessary for GSH stress response. This was further supported by the results showing that Δ*rim11*Δ*ino1* HGT1 strain showed higher GSH sensitivity than Δ*rim11* HGT1 strain, and Δ*ino1* HGT1 strain showed lower GSH sensitivity than Δ*rim11* HGT1 strain (Figure 7B).

In summary, Opi1 is translocated into the nucleus upon GSH addition, where it suppresses the transcription of phospholipid biosynthetic genes. However, as GSH activates the function of the Ume6 protein, the expression of *CHO1*, *CHO2*, and *OPI3* should have increased, but they were also suppressed. GSH likely affects several transcriptional regulatory mechanisms that collectively suppress the overall expression of the phospholipid biosynthetic genes. We speculate that Rim11 partially alleviates GSH stress by regulating the expression of at least some genes involved in the derepression.

We then compared our RNA-seq results (250 µM GSH for 120 min) with the DNA microarray results (50 µM GSH for 30 min) reported by Kumar *et al*., (2011). In contrast to the current results showing the decrease of *INO1* mRNA by 3.9-fold following GSH addition, previous reports showed that transcription of the *INO1* gene was increased by 4.2-fold after GSH addition (Kumar *et al*., 2011). Considering that *INO1* and *HNM1* were the only genes containing the UAS_INO_ sequence in Kumar *et al*. (2011) and expression of no other genes containing the UAS_INO_ sequence were analyzed, involvement of Opi1 and the reason for the increase in *INO1* mRNA by GSH is unclear. We hypothesize that it may either be due to the different strains used in the two studies (YPH499 in Kumar *et al*., 2011 vs. BY4741 in the current study), different conditions of GSH treatment (50 µM GSH for 30 min in Kumar *et al.*, 2011 vs. 250 µM GSH for 120 min in the current results), or the different expression levels of *HGT1*, which leads to different intracellular concentrations of GSH. We found it difficult to draw general conclusions from the two results. However, analyzing transcriptomic changes under different concentrations of GSH or treatment time may be important to further understand the GSH stress response.

Interestingly, DEG analysis using RNA-seq experiments (Figure 5D) revealed a marked increase in the expression of *ULI1* following overexpression of Rim11 (Figure 5D, *iv*); Supplemental Table 2), and a marked decrease in its expression when a kinase-dead form of Rim11 was expressed (Figure 5D, *i*), *ii*), *iii*)). Although the precise function of *ULI1* remains unknown, it is the transcriptional target of Hac1 that is most highly induced (Van Dalfsen *et al*., 2018). GSH stress also induces an ER stress response in *S*. *cerevisiae,* and activated Hac1 upregulates the expression of the ER chaperone gene, *KAR2* (Kumar *et al*., 2011). In addition to inducing ER chaperones, Hac1 upregulates phospholipid biosynthetic genes by antagonistically binding to the Ino2-Ino4 heterodimer with Opi1 (Cox *et al*., 1997; Brickner and Walter, 2004; Schuck *et al*., 2009). Furthermore, we demonstrated that Rim11, by acting downstream of Ire1, helps cope with GSH stress via a mechanism different from the ER stress response (Figures 1C and 3). These findings suggest that Rim11 may cooperate with Hac1 and Uli1 to optimally control the levels of various phospholipid species, which may lead to increased GSH stress tolerance (Figure 8C). However, further studies are needed to address whether myo-inositol 3-phosphate plays an important role in GSH stress alleviation and/or whether changes in phospholipid composition are important for coping with GSH stress.

### Future directions

In addition to its role as a regulator of meiosis, Rim11 is involved in DNA replication stress response (Demin *et al*., 2017). In this study, we report a novel function of Rim11 as a transcriptional regulator of phospholipid biosynthetic genes in a manner dependent on its kinase activity under GSH stress conditions. These findings suggest that Rim11 may have a broader role in flexibly adapting to environmental changes than previously imagined. In addition, when *MRK1*, a paralog of *RIM11*, was deleted or overexpressed, the cells displayed a phenotype similar to that of Δ*rim11* or *RIM11* OE strains (Figure 2). As *RIM11* and *MRK1* belong to the yeast GSK-3β family and constitute mammalian GSK-3β orthologues, our findings may help to further elucidate the mechanisms by which GSH homeostasis is maintained in eukaryotes, including humans. Our study identified potential targets in the GSH stress tolerance mechanism that may contribute to efficient breeding of yeast strains with enhanced production of GSH for various industrial applications.

## MATERIALS AND METHODS

Request a protocol through *Bio-protocol*.

### Chemicals and reagents

All the compounds and reagents used in the study were of analytical or biological grade, and were obtained commercially. The general chemicals were purchased from FUJIFILM Wako Pure Chemical Corporation (Wako, Osaka, Japan) or Sigma-Aldrich Company Limited (Sigma, Cambridge, UK), unless otherwise indicated.

### Yeast strains and cultures

All *S. cerevisiae* strains used in this study are listed in Supplemental Table 3. The host strain, BY4741 (WT), was used from the laboratory stock. Single gene deletion strains with a genetic background of BY4741 were purchased from Yeast MATa Knock Out Strain Collection (Horizon Discovery, Cambridge, UK), and deletions were verified by yeast colony PCR using a primer set, partial sequence of the kanMX4 gene (5′-TTAGAAAAACTCATCGAGCA-3′), and homologous oligo-sequence for the upstream region of the target genes. SD medium containing 20 g/l glucose, 1.7 g/l Difco Yeast Nitrogen Base (YNB) without (NH_4_)_2_SO_4_ or amino acids (Becton Dickinson and Company, BD, Maryland, USA), 5 g/l (NH_4_)_2_SO_4_ with 3 mg/l lysine, 2 mg/l tryptophan, 10 mg/l leucine, 2 mg/l histidine, 2 mg/l adenine, and 2 mg/l uracil was used, and the transformants were screened or selected on the appropriate SD drop out medium.

For biological analyses, yeast cells were cultured in SC medium (containing 20 g/l glucose, 1.7 g/l YNB without [NH_4_]_2_SO_4_ or amino acids, 5 g/l [NH_4_]_2_SO_4_, 2 g/l casamino acids [BD] with 2 mg/l adenine, 2 mg/l uracil, 3 mg/l histidine, and 2 mg/l tryptophan) or SPM, 10 g/l potassium acetate (Kassir and Simchen, 1991; Inai *et al*., 2007). SC medium without uracil (SC-ura) was used to culture mutants harboring the *URA3* marker plasmid. Cell growth was monitored by measuring the optical density at 600 nm with a Spectrophotometer U-5100 (Hitachi, Tokyo, Japan). An OD_600_ × unit was estimated to correspond to ∼1.5 × 10^8^ cells. Precultures that were cultivated overnight in SC or SC-ura were diluted in 10 ml fresh SC or SC-ura medium at OD_600_ = 0.25 and shaken under aerobic conditions at 30°C using a BioShaker BR-43FL (Taitec Corp., Saitama, Japan). GSH stress or nutritional starvation was induced by the addition of GSH (50–1,000 µM) or by the substitution of SC or SC-ura with SPM medium once the cells grew exponentially (OD_600_ = 1.0), respectively, and the cells were further grown for 2 h under the same conditions.

### Construction of plasmids and yeast strains

All the plasmids and oligonucleotides used in this study are summarized in Supplemental Tables 4 and 5. Plasmids were generated using standard restriction enzymes (New England Biolabs, Ipswich, MA, USA) and the InFusion system (TaKaRa Bio, Kusatsu, Japan). All the PCR-amplified sequences were verified by DNA sequencing analysis. For all transformants generated by homologous recombination, yeast colony PCR was used to confirm that the replacement or insertion occurred at the expected locus. To generate multiple knockout mutants, PCR-based homologous recombination was used to replace the entire ORF with the *HIS3* or *URA3* cassette fused with 50 bp identical to the up- and downstream regions of the ORF (Longtine *et al*., 1998).

To produce strains overexpressing *HGT1* or GFP-*HGT1*, p*HGT1*, and pGFP-*HGT1* plasmids were constructed as follows: The ORF sequence for yeast-codon-optimized enhanced GFP (hereafter referred to as GFP) was synthesized and purchased from Fasmac Co., Ltd. (Kanagawa, Japan). The *LEU2* marker sequence was subcloned into pBluescript II SK (+), and then the 5′ UTR of *HGT1* (the DNA segment from –722 bp to –177 bp, the region upstream of the ATG initiation codon) and *TDH3* promoter (*TDH3pr*, regions from –680 bp to –1 bp) were linked adjacent to both sides of *LEU2*. Each ORF of *HGT1* and GFP-*HGT1* (GFP fused to the N-terminus of *HGT1*) was placed directly downstream of *TDH3pr* in p*HGT1* and pGFP-*HGT1*, respectively. A linker sequence, 5′-GGTGGT-3′ (translates to Gly-Gly), was inserted between the GFP C-terminus and *HGT1* N-terminus in pGFP-*HGT1*. p*HGT1* or pGFP-*HGT1* was linearized using the restriction enzyme *Xho*I and introduced into the WT or gene deletion mutant via double-crossover recombination using lithium acetate method (Gietz and Woods, 2002; Gietz and Schiestl, 2007), and then transformants, HGT1 strain or their derivatives, were selected on the SD-leu agar plates.

The expression plasmid harboring 3 × hemagglutinin (3HA)-tagged *RIM11*, p*RIM11pr*-3HA-*RIM11*, was constructed by subcloning the *RIM11* promoter sequence (*RIM11pr*, –1 bp to –475 bp), 3HA fragment, *RIM11* ORF, and 5′ UTR of *RIM11* (–1,305 bp to –851 bp) into pRS303 (*ARS HIS3*). *RIM11pr* in this plasmid was replaced with *TDH3pr*, to generate p*TDH3pr*-3HA-*RIM11*. p*RIM11pr* (or *TDH3pr*)-3HA-*RIM11*^K68A^ was generated using a PCR-based site-directed mutagenesis tool (Fisher and Pei, 1997). To generate the yeast mutants, HGT1 3HA-*RIM11* or HGT1 3HA-*RIM11* OE (abbreviation of “overexpressed”) strain, and HGT1 3HA-*RIM11*-K68A or HGT1 3HA-*RIM11*-K68A OE strain, p*RIM11pr* (or *TDH3pr*)-3HA-*RIM11* and p*RIM11pr* (or *TDH3pr*)-3HA-*RIM11*^K68A^ were digested with *Eco*RI/*Spe*I and HGT1 strains were transformed with the linearized expression cassette by double-crossover homologous recombination, followed by selection on SD-leu-his agar plates.

p*IRE1*-GFP was constructed as previously described (Aragón *et al*., 2009). Briefly, a part of the *IRE1* ORF (from the ATG initiation codon to 2,616 bp in the full length of 3,348 bp) and 5′ UTR (from –1,000 bp to –685 bp) of the *IRE1* ORF were cloned into pRS306 (*ARS URA3*), and the GFP sequence was subcloned between 1,713 bp and 1,714 bp in the *IRE1* ORF, which is the boundary between the ER luminal stress-sensing and kinase domains. p*IRE1*-GFP was linearized by digestion with *Hind*III/*Sac*I and then transformed into the HGT1 strain or Δ*rim11* HGT1 strain by double-crossover homologous recombination at the *IRE1* genomic locus. HGT1 *IRE1*-GFP or Δ*rim11* HGT1 *IRE1*-GFP strains were obtained by screening transformants on SD-leu-ura agar plates.

p*HXT1*-GFP, p*HXT2*-GFP, and p*INO1*-GFP were generated as follows: First, pGFP-*THD3ter-URA3* was constructed. Terminator region (from 1,000 bp to 1,580 bp) of *TDH3* was cloned into pBluescript II SK(+), and GFP and *URA3* that was subcloned from pGFP-HGT1 plasmid above mentioned and pRS306, respectively, were linked adjacent to both sides of *TDH3* terminator region. Next, a part of the *HXT1*, *HXT2*, or *INO1* ORF (from 967 bp to 1,710 bp, from 561 bp to 1,623 bp, or from 441 bp to 2,598 bp, respectively), a PCR-generated GFP-*TDH3ter*-*URA3* sequence using the pGFP-*THD3ter-URA3* as a template, and 3′-UTR of *HXT1*, *HXT2*, or *INO1* (from 2,001 bp to 2,624 bp, from 2,428 bp to 2,882 bp, or from 1,760 bp to 2,333 bp, respectively) were joined in this order into pBluescript II SK(+) by using the InFusion system.

Primers were designed as linker sequence, 5′-GGTGGT-3′ (translates to Gly-Gly), was inserted between C-terminus of the *HXT1*, *HXT2*, or *INO1* ORF and upstream of GFP sequence. Each of p*HXT1*-GFP, p*HXT2*-GFP, and p*INO1*-GFP was linearized by digestion with *Kpn*I/*Bgl*II, *Kpn*I/*Sal*I, and *Bgl*II/*Kpn*I, and then all of them were transformed into the HGT1 3HA-*RIM11*, HGT1 3HA-*RIM11* OE, and HGT1 *RIM11*-K68A OE strains by double-crossover homologous recombination at *HXT1*, *HXT2*, or *INO1* genomic locus, respectively. Transformants were obtained by screening them on SD-leu-his-ura agar plates.

pGFP-*OPI1* (Young *et al*., 2010) was generated as follows. The *PHO5* promoter region (from –623 bp to –1 bp), GFP sequence, spacer segment (5′-GGTGCT-3′, translated to Gly-Ala), *OPI1* ORF, and 5′ UTR of *OPI1* (from –1,000 bp to –359 bp) were subcloned in this order into pRS306 (*ARS URA3*). pGFP-*OPI1* linearized by digestion with *Sal*I/*Eco*RI was introduced into the HGT1 strain, and the transformants were selected on SD-leu-ura agar plates. The resulting colonies (HGT1 GFP-Opi1 strain) were checked for correct integration of the construct by colony PCR

To construct a 3 × FLAG (DYKDDDDK)-tagged *UME6* expression plasmid, pRS316-3FLAG-*UME6* (Xiao and Mitchell, 2000), the promoter region (from –1,000 bp to –1 bp), full- length ORF (up to 2,511 bp), and terminator region (from 2,512 bp to 3,039 bp) of *UME6* were amplified by PCR and the DNA products were inserted into *Bam*HI/*Xho*I-digested pRS316 (*CEN URA3*). A 3 × FLAG-tag sequence was synthesized and fused to the N-terminus of the *UME6* ORF. Δ*ume6* HGT1 3HA-*RIM11* strain was transformed with this plasmid, and the Δ*ume6* HGT1 3HA-*RIM11* 3FLAG-*UME6* strains were selected on SD-leu-his-ura agar plates.

### Genetic suppressor screen

A *S. cerevisiae* chromosomal multicopy library from a laboratory stock (vector; pRS426 [*2µ URA3*], average length of inserts: 6.6 kbp; Kosodo *et al*., 2001) was used to screen for multicopy suppressors of growth defects caused by GSH stress. HGT1 strain was transformed with the library or pRS426 vector without an insert (the latter referred to as “control”), and grown at 30°C for 3–4 d on SD-leu-ura agar plates containing 100 µM GSH. Yeast colonies larger than the control colonies were picked and then stamped onto fresh SD-leu-ura agar plates without GSH (replicates). These strains were then streaked onto two types of selective media, SD-leu-ura agar plates with 250 µM GSH or with 1.0 g/l 5-fluoroorotic acid monohydrate (5-FOA). Strains that were viable in the former, but not viable in the latter, were selected, and the suppressor plasmids were extracted and purified from these cells. DNA sequencing of the insert (chromosomal segment) was performed using the M13 forward/reverse (5′-GTAAAACGACGGCCAGT-3′/5′-CAGGAAACAGCTATGAC-3′, respectively) primer set and identified by BLAST searches of the *Saccharomyces* Genome Database (SGD, www.yeastgenome.org/; Supplemental Table 1). All the potential GSH stress suppressor genes (listed in Supplemental Table 1) were cloned into pRS426 (*2µ URA3*), introduced into the HGT1 strain, and multicopy suppressors were finally identified by confirming reproducible recovery of growth phenotypes under GSH stress conditions.

### Growth measurements and spot assay

The growth phenotypes of the yeast cells were tested using growth curve analysis and/or spot test. To plot the time course of growth, overnight cultures growing in the appropriate medium were added to 4 ml SC or SC-ura at an OD_600_ of 0.1, and then cultivated at 30°C with shaking at 40 rpm using a compact rocking incubator TVS062CA (Advantec Co., Osaka, Japan). The OD_660_ of the growing cells was recorded every 30 min. Spot tests were performed as described below. Cells were grown in SC or SC-ura overnight, diluted to OD_600_ of 2.5 in distilled water, and 10-fold serial dilutions were spotted onto the indicated agar plates (see Figure legends). The plates were incubated at 30°C.

### Microscopy

All microscopic images were acquired using the BZ-X700 system (Keyence Corp., Osaka, Japan). The excitation/emission wavelengths of the laser and dichroic mirror were 470 ± 40 nm/525 ± 50 nm and 495 nm for DAPI (Dojindo Laboratories, Kumamoto, Japan) and Hoechst 33342 solutions (Dojindo), respectively. Those for GFP and Alexa Fluor488-conjugated anti-mouse IgG goat antibody (#4408, Cell Signaling Technology, Danvers, MA, USA) were 525 ± 25 nm/605 ± 70 nm and 565 nm, respectively. A total of 3–5 fields were examined for each biological sample. Line-profile data were acquired by using BZ-X Analyzer (version 1.3.1.1; Keyence).

### Preparation of total cell lysate for sodium dodecyl sulfate polyacrylamide gel electrophoresis (SDS–PAGE)

For analyses of 3HA-Rim11, 3FLAG-Ume6, and Hxt1/2-GFP, a total of 1.5 × 10^9^ cells (10 OD_600_ units) were harvested from the indicated cultures, and total cell lysates were prepared using trichloroacetic acid (TCA) as previously described (Noda *et al*., 2019; Laussel *et al*., 2022). Briefly, the harvested cells were mixed with 6% TCA and incubated on ice for 15 min. Cells were collected by centrifugation at 10,800 × g for 10 min at 4°C, and the pellets were suspended in 1 ml of ice-cold 70% (vol/vol) ethanol and stored at –80°C until use. For preparing lysates containing 3HA-Rim11or 3FLAG-Ume6, the pelleted cells were collected again by centrifugation and resuspended in 200 µl urea buffer (50 mM Tris-HCl, pH 7.5, 5 mM EDTA, 6 M urea, 1% sodium dodecyl sulfate [SDS], 50 mM NaF) supplemented with 1 × protease inhibitor cocktail (cOmplete EDTA-free, Catalog no. 11873580001, Roche Diagnostics GmbH, Mannheim, Germany), and then disrupted with 200 mg acid-washed glass beads (particle size 0.5 mm, Roche) using a Multi-beads shocker (Yasui Kikai Corp., Osaka, Japan) for five cycles of 1 min each (1-min intervals). After centrifugation at 10,800 × g for 10 min at 4°C, 90 µl of the supernatant was added to 30 µl of 4 × Laemmli buffer with or without 2-mercaptoethanol and heated at 65°C for 5 min.

To prepare Hxt1/2-GFP lysates, frozen cells stored at –80°C were thawed and resuspended in 200 µl of 10% TCA solution and disrupted with 200 mg acid-washed glass beads as mentioned above. Samples were transferred to another tube and centrifuged at 16,000 × g for 5 min at 4°C. After the supernatants were removed, protein pellets were washed once with distilled water, spun down, and the wash water was discarded. The pellets were resuspended in 300 µl of sample buffer (50 mM Tris-HCl [pH 8.8], 100 mM DTT, 2% SDS, 0.1% bromophenol blue, and 10% glycerol] and heated at 37°C for 5 min.

For analyses of Ino1-GFP, 3 × 10^8^ cells (2 OD_600_ units) were used from the indicated cultures, and total cell lysates were prepared according to the former method (Calzada *et al*., 2019) as follows. The cell pellet was suspended in 150 µl of freshly prepared NaOH/2-mercaptoethanol solution consisting of 1 ml of 2 M NaOH and 80 µl of 2-mercaptoethanol, and incubated for 10 min on ice. The suspension was then mixed with 75 µl of 100% (wt/vol) TCA to precipitate target proteins, incubated for 10 min on ice, and then centrifuged at 21,000 × g for 2 min at 4°C. After the supernatant was removed, cold acetone was added to rinse the pellet, spun down, and decanted. After air-drying, the pellet was resuspended in 45 µl of 0.1 M NaOH, mixed with 4 × Laemmli buffer with 2-mercaptoethanol, and heated at 95°C for 5 min.

### Western blotting and quantification

Stain-free imaging technology (Bio-Rad Laboratories, Hercules, CA, USA) was used to visualize and quantify the total protein in each lane (served as a loading control). Samples were separated on a polyacrylamide gel TGX Stain-Free FastCast 7.5% (#1610181, Bio-Rad) or 10% (#1610183, Bio-Rad) precast gels. Short UV irradiation stimulated the formation of covalent bonds between the trihalo-compounds and tryptophan residues in protein samples. Following transfer of the proteins to a polyvinylidene difluoride (PVDF) membrane (0.45 µm), the membrane was blocked with EveryBlot Blocking Buffer (#12010020, Bio-Rad). The membrane was then incubated with diluted primary antibody overnight at 4°C, rinsed, and incubated with diluted secondary antibodies for 1 h at room temperature (RT). All primary antibodies were diluted 1:1000 in Tris Buffered Saline with Tween 20 (TBST) (50 mM Tris-HCl, pH 7.4, 150 mM NaCl, and 0.05% Tween 20) supplemented with 5% (vol/vol) EveryBlot Blocking Buffer and 0.02% NaN_3_. All secondary antibodies were prepared at a 1:5000 dilution in TBST with 0.02% SDS and 5% (vol/vol) EveryBlot Blocking Buffer. The primary antibodies used were anti-phosphotyrosine mAb (Sigma, 4G10 Platinum, #05-1050), anti-HA rAb (Medical & Biological Laboratories Co., Tokyo, Japan, Catalog no. 561), anti-GFP rAb (Medical & Biological Laboratories Co., Tokyo, Japan, Code No 598), and anti-Flag mAb (Sigma, #F1804). The secondary antibodies used were Alexa Fluor488-conjugated anti-mAb goat antibody (Cell Signaling Technology; #4408) and Alexa Fluor plus800-conjugated anti-rAb goat antibody (Invitrogen Corporation, CA, USA, #A32735). UV irradiation of the gel and membrane scanning were performed using the ChemiDoc MP Imaging System (Bio-Rad), and images were acquired and analyzed using the Image Lab software (version 6.1, Bio-Rad). Uncropped images of the blots cited in the main text are provided in the Supplemental Materials. The signal intensities of the target proteins were normalized to the amount of total protein in each lane, and the relative quantities were calculated. Statistical analysis was performed using more than three biological replicates and reproducibility was confirmed in at least two independent experiments.

### Indirect immunofluorescence

Intracellular distribution of 3HA-Rim11 or 3HA-Rim11 K68A in yeast cells under GSH stress conditions were observed using an indirect immunofluorescence method as described previously (Sato *et al.*, 2007). Cells growing exponentially at 30°C in SC in the presence of 250 µM GSH for 2 h were fixed by adding 10% paraformaldehyde (2.5 ml to 7.5 ml of yeast culture) and pelleted by centrifugation. The pelleted cells were resuspended in 3.2 ml potassium phosphate (PP, 0.1 M PP, pH 7.5), mixed with 1.8 ml of the paraformaldehyde solution, and fixed for 15 min at RT with gentle shaking. Fixed cells were washed four times in PP, resuspended in 1 ml of PP containing 1.2 M sorbitol (potassium phosphate-buffered sorbitol solution [SPP]) and 100 mM DTT, and then treated with 25 µg of Zymolyase-100T (Seikagaku Biobusiness Corp., Tokyo, Japan) for 30 min at 30°C. The resulting spheroplasts were harvested by centrifugation, resuspended in 50 mM NH_4_Cl in SPP, and then resuspended in SPP without NH_4_Cl. The cell suspensions were transferred to slides coated with polylysine (poly-L-lysine hydrochloride, Peptide Institute, Osaka, Japan, #3075) and incubated for 30 min at RT, followed by removal of the supernatants. The slides were then immersed in methanol (6 min) followed by acetone (30 s) at –20°C, and then air-dried. Anti-HA mAb (Medical & Biological Laboratories Co, Tokyo, Japan; #M132-3) was diluted 1:100 in TBST-B (TBST containing 1% skim milk and 0.1% bovine serum albumin), added to the slides, and incubated overnight at 4°C in a tight box with layered wet paper towels inside. Slides were washed in TBST-B and incubated for 2 h at RT in the secondary antibody solution (Alexa Fluor488-conjugated anti-mAb goat antibody; Cell Signaling Technology, #4408) diluted 1:250 in TBST-B. Finally, the slides were washed with phosphate-buffered saline, mounted with the mounting solution (Kilmartin and Adams, 1984) containing DAPI solution, and a coverslip was applied. Images were obtained using the BZ-X700 system (Keyence Corp.).

### Measurement of total and oxidized GSH

Total GSH was determined according to a previously described method (Rahman *et al*., 2006) with minor modifications. Cells (2 OD_600_ units) grown in the early exponential phase in the presence or absence of GSH were collected by centrifugation at RT. Cells were washed twice with distilled water, suspended in 100 µl of 0.1% 5-sulfosalicylic acid solution (5-SSA), and then heat-treated at 100°C for 5 min. After centrifugation at 10,800 × g for 5 min at 4°C, the supernatant (80 µl) was collected as an analytical sample for the estimation of total GSH (T-GSH = GSH + GSSG). Samples for GSSG quantification were prepared by adding 2 µl of 2-vinylpyridine to 80 µl of the cell supernatant and incubated at RT for 60 min. The colorimetric assay was performed in 96-well plates using a microplate reader ARVO X3 (PerkinElmer, Waltham, MA, USA) by reading the absorbance at 405 nm. For both T-GSH and GSSG quantitations, 120 µl of freshly prepared reaction solution (100 mM PP, pH 7.5, containing 5 mM EDTA) supplemented with 0.67 mg/ml 5,5′-Dithiobis (2-nitrobenzonic acid; DTNB), 0.67 mg/ml β-NADPH (Oriental Yeast Co. Tokyo, Japan), and 0.1 IU/ml glutathione reductase (GR, Oriental Yeast, 200 international units [IU], #46540005) was mixed with 80 µl of cell supernatant in each well. The OD_405_ at this point was set as the 0-min time point measurement, and OD_405_ was measured every 5 min for 20 min. The rate of 2-nitro-5-thiobenzoic acid formation was then calculated from the changes in the absorbance as a function of time (in min). To generate standard curves, pure GSH and GSSG (Sigma-Aldrich) were dissolved in 0.1% 5-SSA, and 2 µl of 2-VP was added to 80 µl of the GSSG solution. The concentrations in the sample extracts were determined using linear regression analysis from each standard curve and expressed as T-GSH or GSSG content in the cells [%] using the equation: 2 OD_600_ units = ∼ 300 µg dry cell. GSH (reduced form) concentration was calculated by subtracting the values of GSSG from those of T-GSH. This assay was performed in triplicate for each sample, and the mean of the three samples is presented as the value of one biological sample (*n* = 1).

### Total RNA isolation, RNA-seq, and gene expression analysis

For the RNA-seq, experiments were performed using three biological replicates (*n* = 3) for each biological condition. HGT1 3HA-*RIM11*, HGT1 3HA-*RIM11*-K68A, HGT1 3HA-*RIM11* OE, and HGT1 3HA-*RIM11*-K68A OE strains grown under GSH stress condition (250 µM GSH for 2 h) were harvested by centrifugation at 5,000 × g for 3 min at RT. After removing the supernatant, the pellets were immediately frozen (without washing) in liquid nitrogen and stored at –80°C until use. The hot acid-phenol method (Collart and Oliviero, 1993) was used, and total RNA was isolated using a RNeasy Mini Kit (Qiagen, Hilden, Germany) according to the manufacturer’s protocols. The total RNA was dissolved in RNase-free distilled water.

Quality check of the total RNA, library construction, and sequencing were performed by Annoroad Gene Technology Co. (Beijing, China). RNA quality and concentration were measured using an Agilent 2100 Bioanalyzer (Agilent Technologies, Santa Clara, CA, USA) and NanoDrop 2000 (Thermo Fisher Scientific, Waltham, MA, USA), respectively. RNA-seq libraries were constructed using the NEBNext Ultra RNA Library Prep Kit for Illumina and sequenced on an Illumina HiseqX platform (2 × 150 bp; Illumina, San Diego, CA, USA). From each sample, a total of 2.20–2.38 Gb of clean bases data and 14.7–15.8 Mb clean reads data were obtained (Supplemental Table 6). Using the Trimmomatic-0.36 tool, raw data were processed to remove adapter sequences, unpaired reads, and low-quality reads containing more than 50% bases with a Phred score of Q < 20 or >5% unknown bases (N). Trimmed reads were aligned to the *S. cerevisiae* reference genome (Engel *et al*., 2014) using STAR-2.7.9a tool with a mean mapping rate of 89.19%. The featureCounts program (subread v2.0.1) was applied to count mapped reads per gene with the following options: paired-end; yes, multi-mapping reads; not counted, multi-overlapping reads; not counted, min overlapping bases; 1, chimeric reads; counted, both ends mapped; not required. DEGs were detected using the following cutoff conditions; average log_10_CPM ≥ 1.0, false discovery rate (FDR) < 0.05, and the value of log_2_fc (see main text or Figure legends for details) when necessary. The raw sequence and gene expression data were deposited in the DDBJ/EMBL/GenBank database under accession number DRA013373 and the DDBJ Genomic Expression Archive (GEA) under accession number E-GEAD-475.

### Lipid extraction

Lipid analysis was performed using five biological replicates (*n* = 5) for each biological condition, and was applied to HGT1 3HA-*RIM11*, HGT1 3HA-*RIM11* OE, and HGT1 3HA-*RIM11*-K68A OE strains. Cells precultured in the SC medium for 16 h were transferred to 15 ml of fresh SC medium at OD_600_ = 0.25 and cultivated at 30°C with shaking. Subsequently, GSH stress was induced by treatment with 250 µM GSH per OD_600_ = 1.0, and the cells were further grown for 2 h. A total of 3.0 × 10^9^ cells (20 OD_600_ units) were harvested and crushed with an appropriate amount of 0.5-mm zirconia balls using a Micro Smash MS-100R (Tomy Seiko, Tokyo, Japan) at 5,500 rpm, 30 s, four cycles, 60-s intervals. Lipids were extracted using the BUME method (Löfgren *et al*., 2012) as described below. BUME solution (300 μl, butanol: methanol = 3:1 (*vol/vol*)), 300 µl of a heptane-ethyl acetate solution (heptane: ethyl acetate = 3:1 (*vol/vol*), and 300 µl of 1% acetic acid was added to the broken cell lysates in this order. Every time the solution was added, the mixture was stirred vigorously for 2 min and kept for 5 min at RT. Subsequently, the mixture was centrifuged at 10,000 rpm for 5 min, and the upper layer was collected. Heptane-ethyl acetate solution (300 μl) was added to the remaining lower layer, and the mixture was stirred vigorously for 2 min. After standing for 5 min at RT, the mixture was centrifuged under the same conditions, and the upper layer was mixed with the first upper layer solution. The obtained total upper layers were evaporated under nitrogen gas and used as extracted lipids.

### Targeted lipidomics

Lipidomic analysis of these extracted lipids was performed as previously described (Nakao *et al*., 2019; Watanabe *et al*., 2022). The extracted lipids were dissolved in equal volumes of methanol and acetonitrile, and subjected to the liquid chromatography-mass spectrometry system consisted of a Prominence UFLC system (Shi-madzu, Kyoto, Japan) equipped with a SeQuant ZIC-HILIC column (5 μm, 2.1 mm × 150 mm, Merck Millipore) coupled to a 3200 QTRAP System (Sciex, Redwood, CA, USA). The optimal conditions for the ionization and fragmentation of each lipid were determined as previously described (Watanabe *et al*., 2022).

### Statistical analysis

Statistical significance was assessed using a two-tailed Welch’s *t* test with at least three biological replicates (*n* ≥ 3). Reproducibility was confirmed by performing at least two independent experiments. The sample size used for each experiment is described in detail in the corresponding Figure legend. Statistical significance was set at *p* = 0.05. In RNA-seq analysis, the DEGs cutoff values were as follows: FDR < 0.05, log_10_CPM ≥ 1 or log_2_fc ≥ 1 and log_2_fc ≤ –1.

## Supplementary Material





## Abbreviations used:

CPM: counts per million
DEGs: differentially expressed genes
DTT: dithiothreitol
ER: endoplasmic reticulum
FDR: false discovery rate
GO: gene ontology
GSH: reduced glutathione
GSH stress: GSH-induced stress
GSK-3: glycogen synthase kinase-3
GSSG: oxidized glutathione
HA: hemagglutinin
Hgt1: plasma membrane-localized GSH transporter
NAD: nicotinamide adenine dinucleotide
NADP: nicotinamide adenine dinucleotide-phosphate
OD: optical density
OE: overexpressed
ORF: open reading frame
PC: phosphatidylcholine
PE: phosphatidylethanolamine
PI: phosphatidylinositol
PS: phosphatidylserine
PiTyr: phosphotyrosine
PKA: cAMP-dependent protein kinase
RNA-seq: RNA sequencing
SC: synthetic complete
SD: synthetic dextrose
SGD: *Saccharomyces* Genome Database
SPM: sporulation medium
TCA: trichloroacetic acid
Tm: tunicamycin
UAS _INO_: upstream activation sequence of INO
UPR: unfolded protein response
URS1: upstream repression sequence
UTR: untranslated region
WT: wild-type
