# Supplementary material for: Yeast Rim11 kinase responds to glutathione-induced stress by regulating the transcription of phospholipid biosynthetic genes
Source: Mol Biol Cell. 2023 Dec 14;35(1):ar8. doi: 10.1091/mbc.E23-03-0116 (PMC10881166; doi:10.1091/mbc.E23-03-0116)
Supplement: Supplementary file 1 [file mbc-35-ar8-s001.pdf]

Supplemental Materials

Molecular Biology of the Cell

Yasukawa *et al*.

Supplemental Materials

Molecular Biology of the Cell

Taishi Yasukawa *et al*.

Supplemental Figure 1

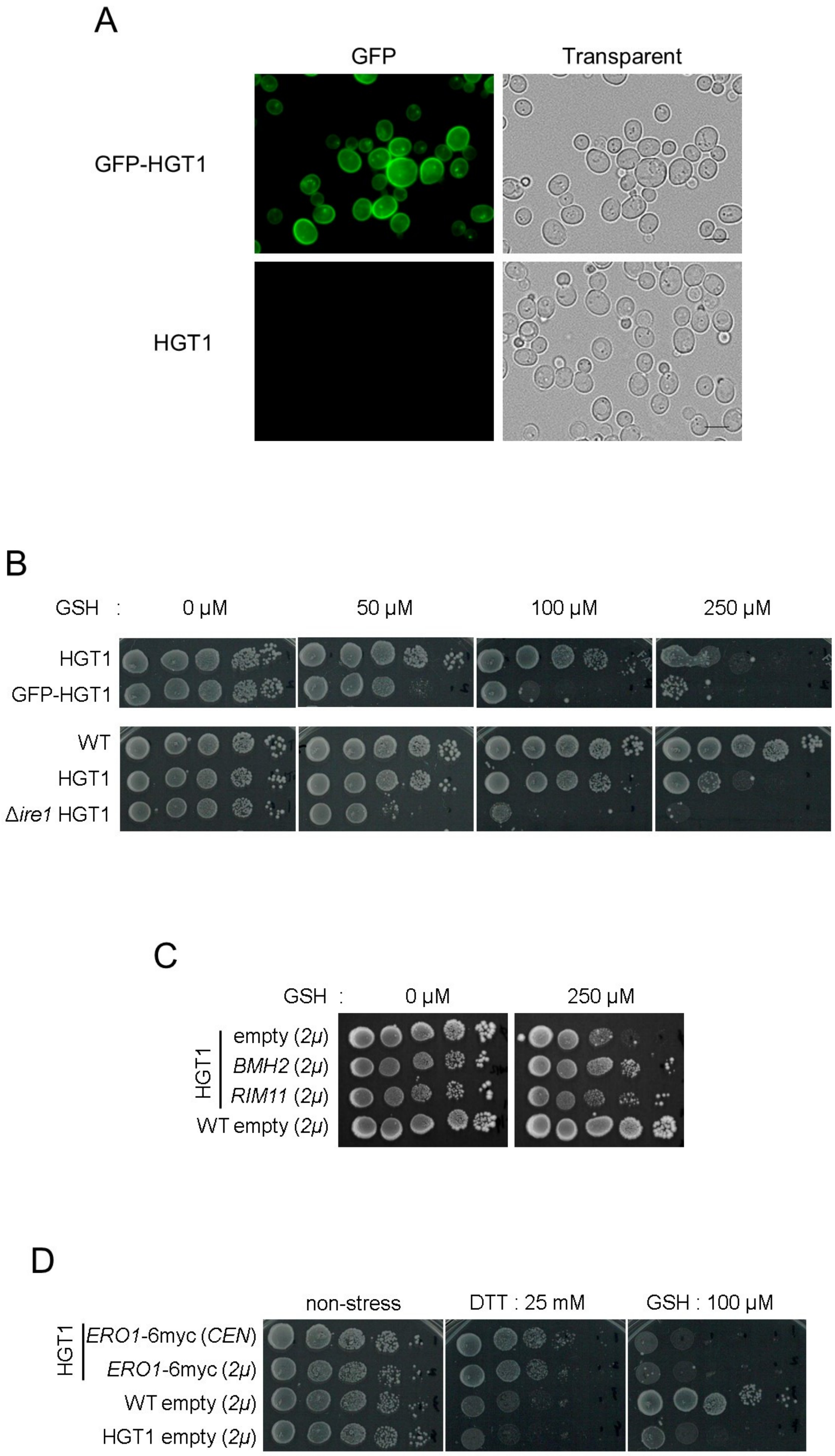

E

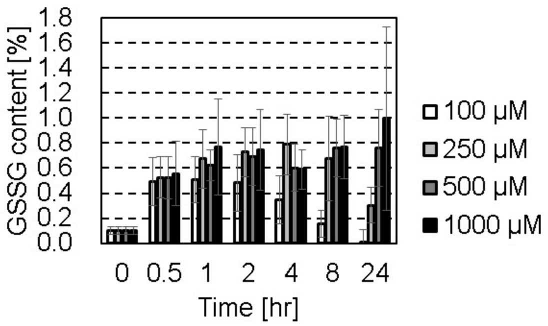

Figure S1. Biochemical and other analyses of various HGT1 derivatives. (A) Intracellular localization of GFP-Hgt1. HGT1 and GFP-HGT1 strains were aerobically cultured in SC medium (SD medium supplemented with adenine, uracil, histidine, and tryptophan) at 30ºC, harvested at log-phase, and observed using a fluorescence microscope without fixation. A total of 3–5 fields were examined for each biological sample. Scale bar, 5 µm. (B, C, D) Growth phenotypes of the indicated strains. Overnight cultures of the strains in (B) SC or (C, D) SC-ura were adjusted to OD_{600} of 2.5 and serially diluted 10-fold with distilled water followed by spotting on (B) SC or (C, D) SC-ura agar plates with or without the indicated concentration of GSH or DTT, and then incubated at 30ºC. (C) *BMH2* and *RIM11* or (D) *ERO1*-6myc were expressed in the HGT1 strain from a high-copy plasmid, pRS426 (*2µ URA3*) (C) and/or from a low-copy plasmid, pRS316 (*CEN URA3*). (D) WT empty and HGT1 empty, carrying a pRS426 vector, were included as negative controls. (E) Time course of the intracellular GSSG content in HGT1 strain grown in the presence of exogenously added GSH at various concentrations. Cells (2 OD_{600} units) growing aerobically in exponential phase (OD_{600} of approximately 1) in SC at 30ºC were collected at seven timepoints (0, 0.5, 1, 2, 4, 8, and 24 h) following the addition of GSH (100, 250, 500, and 1,000 µM) to the cultures. Harvested cells were washed, suspended in 5-sulfosalicylic acid (5-SSA) solution, heat-treated at 100ºC for 5 min, and supernatants were collected after centrifugation. 2-vinylpyridine was added to the supernatants (extracts) at 1/100 of the extract volume, and the concentration of GSSG was measured using the GR-DTNB assay (see *Materials and Methods*). Bar graphs and error bars represent mean and standard deviation from three independent experiments (n = 6), respectively. GFP: green fluorescent protein; HGT1, plasma membrane-localized GSH transporter; SC, synthetic complete; GSH, reduced glutathione; DTT, dithiothreitol; GSSG, oxidized glutathione; GR-DTNB, glutathione reductase 5,5'-Dithiobis(2-nitrobenzonic acid)

Supplemental Figure 2

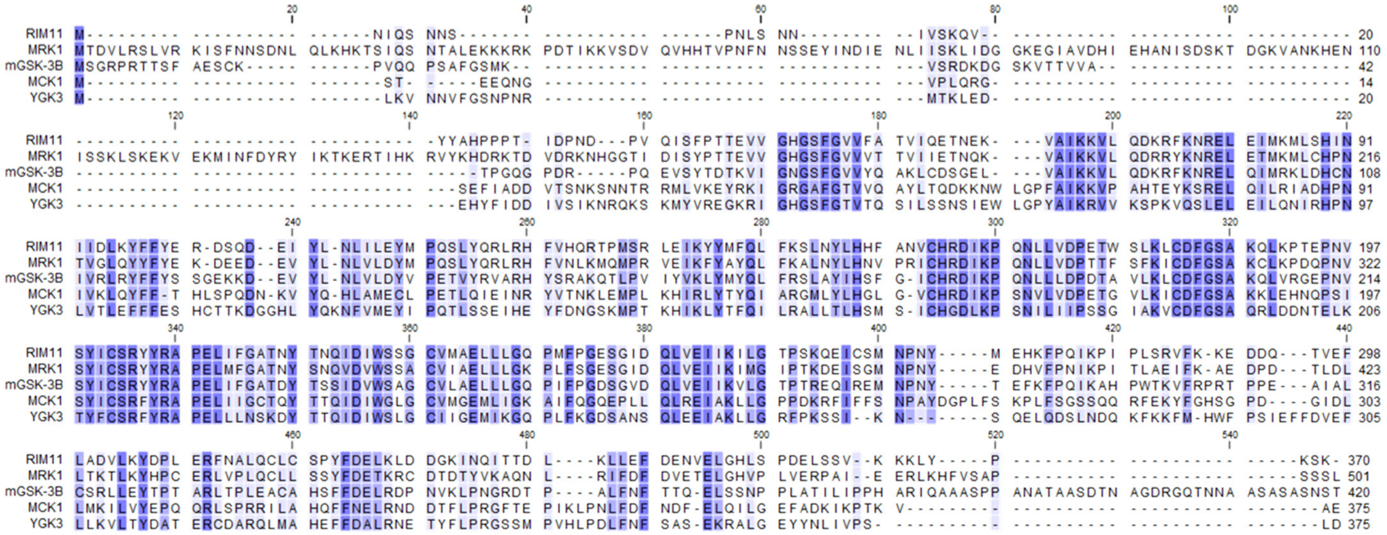

Figure S2. Multiple alignment of the amino acid sequences of mammalian and yeast glycogen synthase kinase-3β family (mGSK-3β and yGSK-3β). CLC Sequence Viewer 8.0 (https://clc-sequence-viewer.software.informer.com/8.0/) was used to generate the alignment. Murine GSK-3β, deposited in GenBank under accession number AAD39258, was included as the representative sequence of mGSK-3β. The percent identities of each amino acid sequence to that of yeast Rim11 are as follows; Mrk1 (62.3%), mGSK-3β (53.3%), Mck1 (41.1%), and Ygk3 (36.9%). These were calculated using NCBI BLASTp (https://blast.ncbi.nlm.nih.gov/Blast.cgi?PAGE=Proteins) using the following parameters: matrix, BLOSUM62; expected threshold, 0.05. Identical and similar amino acids among amino acid sequences are shown in shades of blue.

Supplemental Figure 3

A

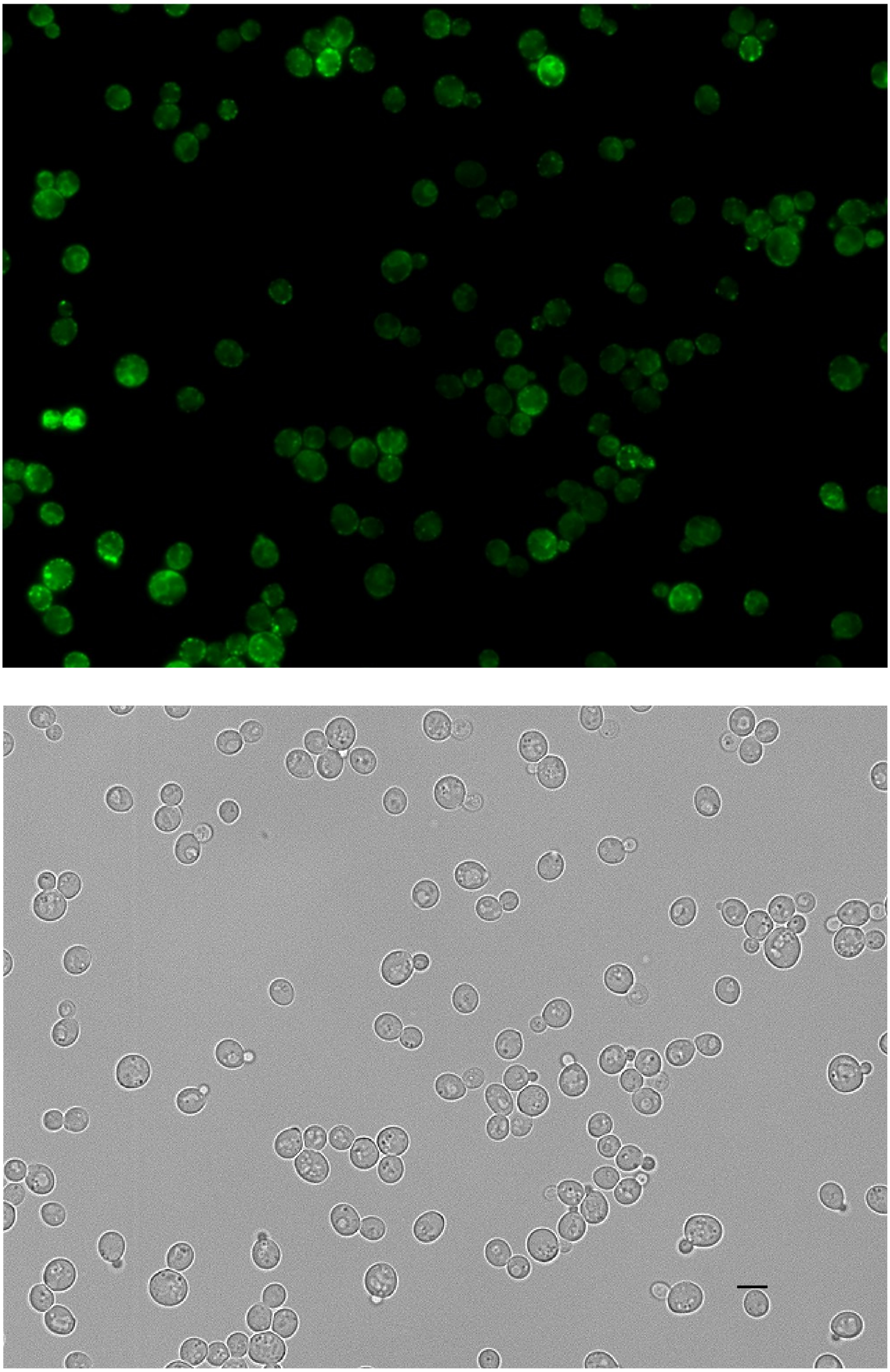

B

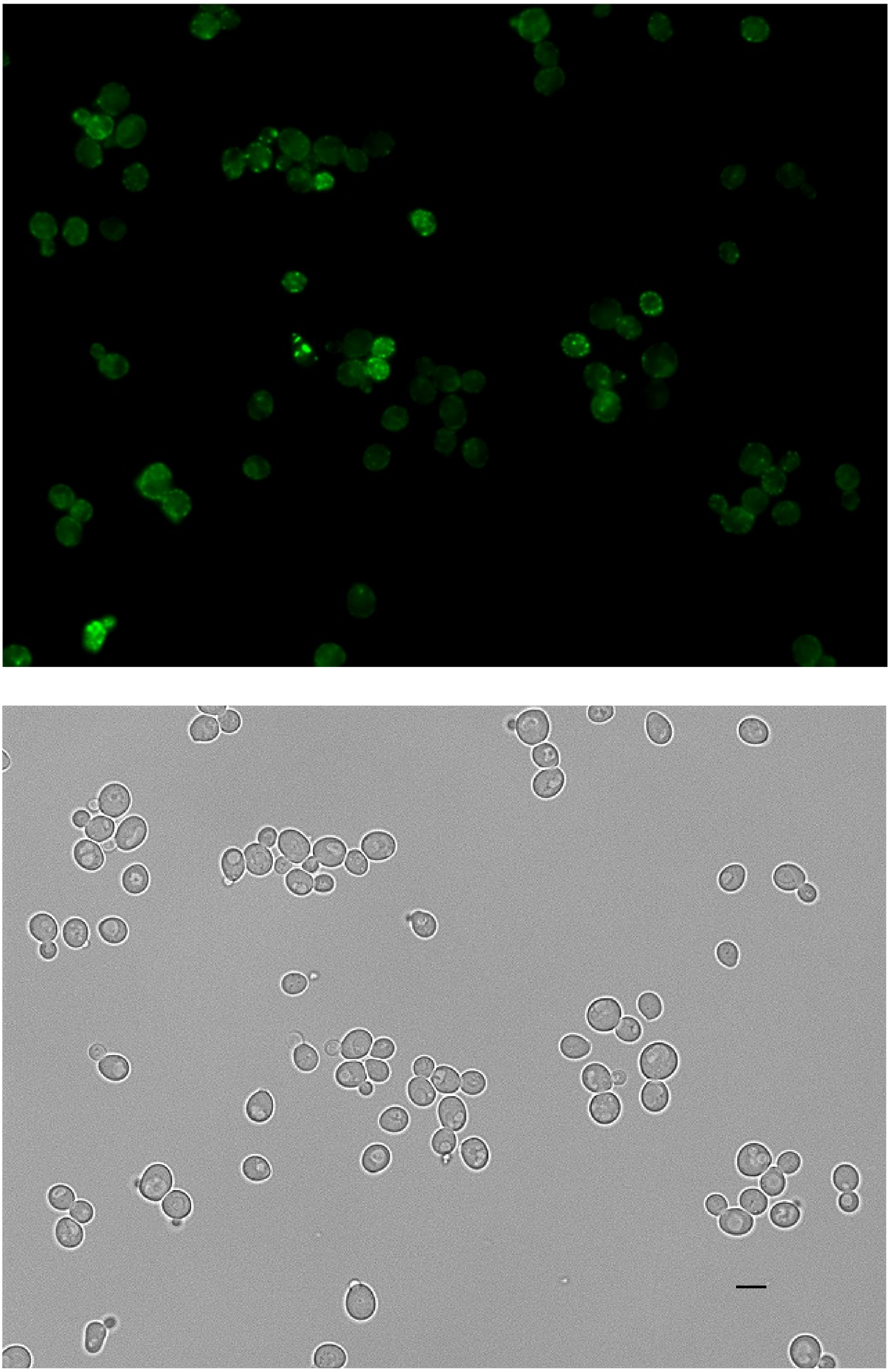

Figure S3. Alternate images of cells under GSH stress (50 µM) shown in main Figure 3C. Scale bar, 5 µm. (A) HGT1 *IRE1*-GFP strain, (B) Δ*rim11* HGT1 *IRE1*-GFP strain. GSH, reduced glutathione; HGT1, plasma membrane-localized GSH transporter; GFP: green fluorescent protein.

Supplemental Figure 4

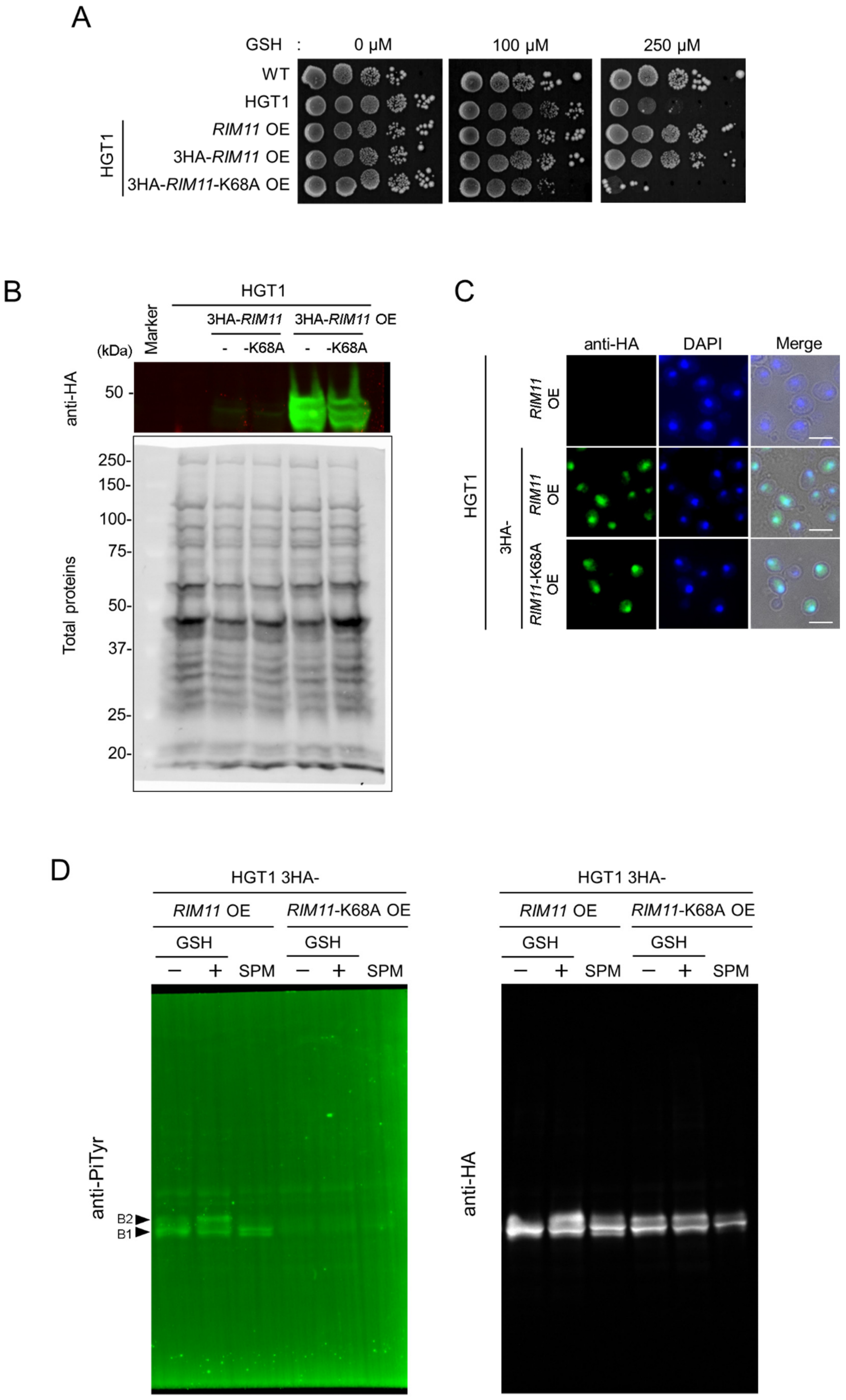

Figure S4. Biochemical and microscopic evaluation of kinase-active or -inactive *RIM11*-overexpressing mutants. (A)

Evaluation of the growth phenotypes of WT and various HGT1 mutants overexpressing *RIM11* or $RIM11^{K68A}$ fused with or without 3× hemagglutinin (3HA) tag (*RIM11*OE, 3HA-*RIM11*OE, or 3HA-*RIM11*-K68A OE strains) under GSH stress-induced or non-induced conditions. Overnight cultures of cells growing in SC were diluted and adjusted to an OD_{600} of 2.5, diluted 10-fold serially, and the suspensions were plated onto SC agar plates with or without GSH, followed by incubation at 30ºC. (B) Images of fluorescent western blotting and total protein profile of HGT1, HGT1 3HA-*RIM11*, HGT1 3HA-*RIM11*-K68A, HGT1 3HA-*RIM11* OE, and HGT1 3HA-*RIM11*-K68A OE strains. Cells cultivated in SC were harvested at the log-phase, fixed in trichloroacetic acid (TCA), and subsequently suspended in ice-cold 70% (v/v) ethanol. Cell lysates were prepared by disrupting the cell pellets with a bead-beater in a buffer containing 6 M urea (see *Materials and Methods*). The following antibodies were used for western blotting: pseudo color green image (primary/secondary), anti-HA rAb/Alexa Fluor488 conjugated anti-rAb goat antibody. Total protein profiles (stain-free technology offered from Bio-Rad, see *Materials and Methods*) were used as loading controls. (C) Indirect immunofluorescence images of HGT1 *RIM11* OE, HGT1 3HA-*RIM11* OE, and HGT1 3HA-*RIM11*-K68A OE strains. The mutants were cultured in SC at 30ºC until the exponential phase and GSH stress was induced by the addition of GSH (250 μM). Cells were grown at 30ºC for another 2 h, harvested by centrifugation, immediately fixed using the formaldehyde method, and observed with a microscope (see *Materials and Methods*). Anti-HA mAb and Alexa Fluor488-conjugated anti-mAb goat antibody were used as primary and secondary antibodies, respectively. Scale bar, 5 μm. (D) Uncropped images of Figure 4C shown in the main figures. The anti-phosphotyrosine mAb image (Left, green image), and the anti-HA rAb image (right, gray-scale image) are shown. Arrows B1 and B2 represent phosphorylated 3HA-Rim11 and Rim11 with enhanced phosphorylation, respectively. WT, wild-type; HGT1, plasma membrane-localized GSH transporter; GSH, reduced glutathione; HA, hemagglutinin; OE, overexpressed; SC, synthetic complete; PiTyr, phosphotyrosine; SPM, sporulation medium; DAPI, 4′ ,6-diamidino-2-phenylindol.

Supplemental Figure 5

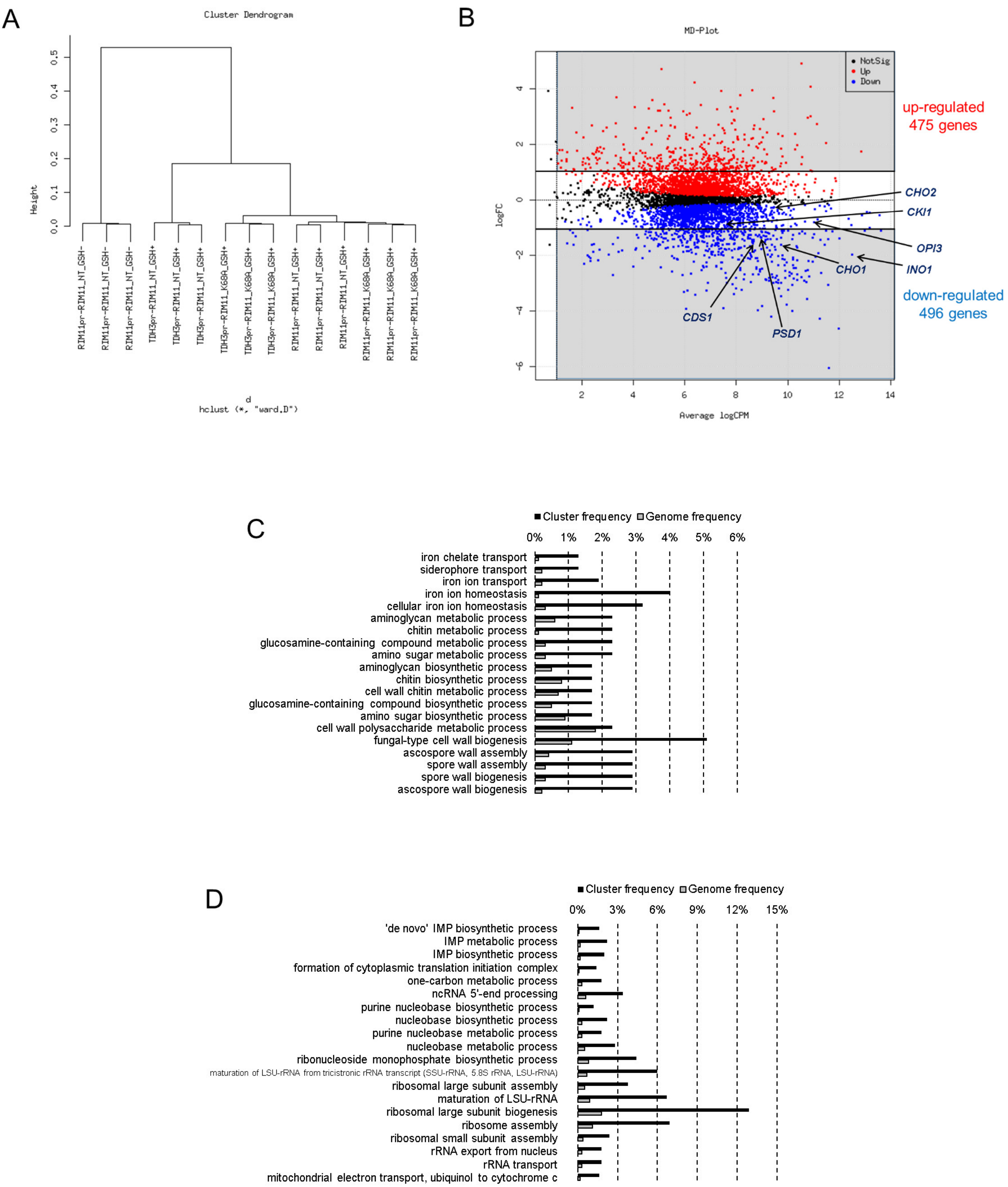

E

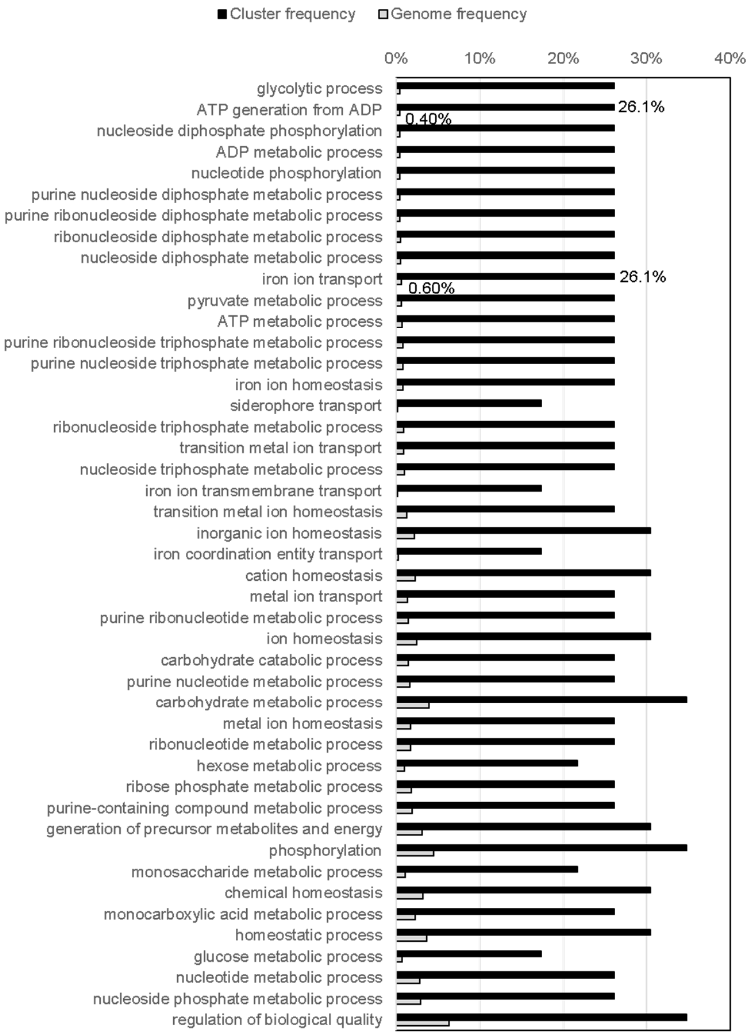

Cluster frequency
Genome frequency
0%
10%
20%
30%
40%
26.1%
0.40%
26.1%
0.60%
glycolytic process
ATP generation from ADP
nucleoside diphosphate phosphorylation
ADP metabolic process
nucleotide phosphorylation
purine nucleoside diphosphate metabolic process
purine ribonucleoside diphosphate metabolic process
ribonucleoside diphosphate metabolic process
nucleoside diphosphate metabolic process
iron ion transport
pyruvate metabolic process
ATP metabolic process
purine ribonucleoside triphosphate metabolic process
purine nucleoside triphosphate metabolic process
iron ion homeostasis
siderophore transport
ribonucleoside triphosphate metabolic process
transition metal ion transport
nucleoside triphosphate metabolic process
iron ion transmembrane transport
transition metal ion homeostasis
inorganic ion homeostasis
iron coordination entity transport
cation homeostasis
metal ion transport
purine ribonucleotide metabolic process
ion homeostasis
carbohydrate catabolic process
purine nucleotide metabolic process
carbohydrate metabolic process
metal ion homeostasis
ribonucleotide metabolic process
hexose metabolic process
ribose phosphate metabolic process
purine-containing compound metabolic process
generation of precursor metabolites and energy
phosphorylation
monosaccharide metabolic process
chemical homeostasis
monocarboxylic acid metabolic process
homeostatic process
glucose metabolic process
nucleotide metabolic process
nucleoside phosphate metabolic process
regulation of biological quality

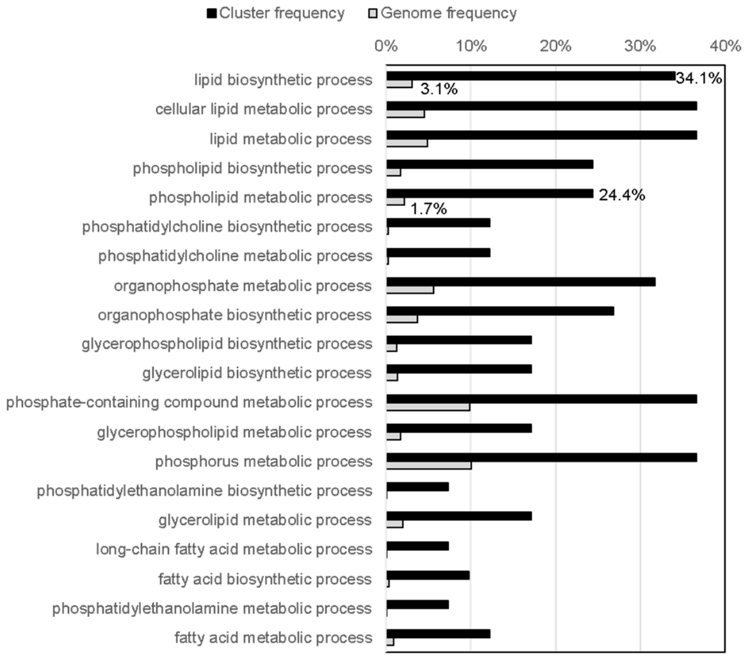

Cluster frequency
Genome frequency
0%
10%
20%
30%
40%
34.1%
3.1%
24.4%
1.7%
lipid biosynthetic process
cellular lipid metabolic process
lipid metabolic process
phospholipid biosynthetic process
phospholipid metabolic process
phosphatidylcholine biosynthetic process
phosphatidylcholine metabolic process
organophosphate metabolic process
organophosphate biosynthetic process
glycerophospholipid biosynthetic process
glycerolipid biosynthetic process
phosphate-containing compound metabolic process
glycerophospholipid metabolic process
phosphorus metabolic process
phosphatidylethanolamine biosynthetic process
glycerolipid metabolic process
long-chain fatty acid metabolic process
fatty acid biosynthetic process
phosphatidylethanolamine metabolic process
fatty acid metabolic process

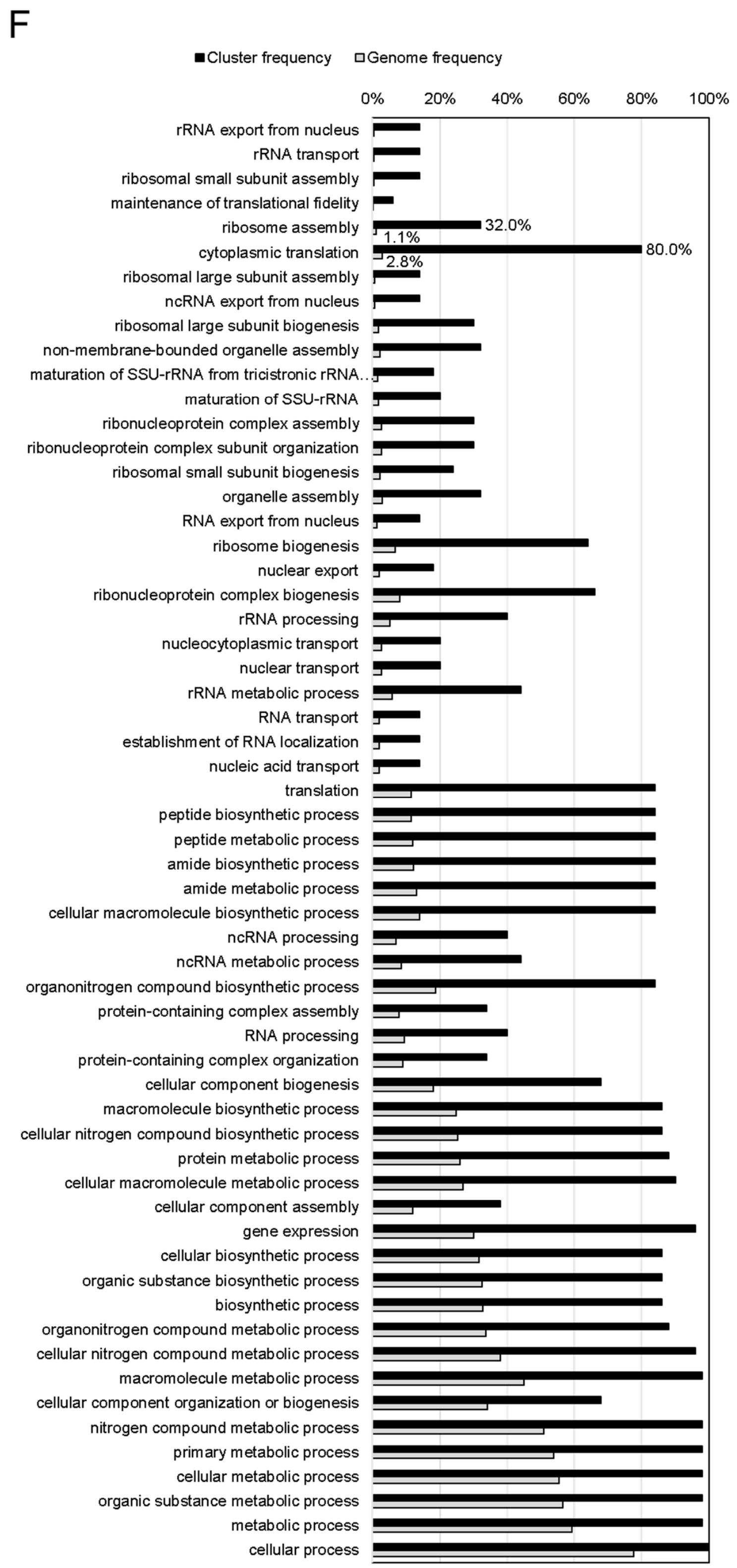

Figure S5. RNA-seq analyses of the HGT1 strain and HGT1 derivatives with mutated *RIM11* with or without GSH stress. (A) Hierarchical clustering analysis for all biological replicates subjected to RNA-seq experiment. Spearman's rank correlation was applied using Ward's minimum variance method. Quality Scores, Q30, of all the trimmed reads were >94% (Supplemental Table 6); hence, the quality of RNA-seq data was considered adequate for the transcriptomic analysis. (B)

Comparison of differentially expressed gene (DEG) levels induced by GSH stress (250 μM GSH) with those under non-stress conditions (0 μM GSH) in the HGT1 strain. In MD-plot, horizontal and vertical axes represent $\log_{10}$CPM (counts per million) and $\log_2$fc (fc; fold change), respectively. Colored dots show FDR < 0.05 and $\log_{10}$CPM ≥ 1.0, and region in gray shows absolute $\log_2$fc ≥ 1. Red and blue dots represent up- and down-regulated genes, respectively. (C, D) Top 20 of the represented gene ontology (GO) terms associated with GSH stress (250 μM GSH) in the HGT1 strain. GO enrichment analyses were performed for (C) 475 upregulated and (D) 496 down-regulated genes using *Saccharomyces* Genome Database (SGD) GO Term Finder (https://www.yeastgenome.org/goTermFinder). Cluster frequency shows the percentage of genes that are annotated for each process per (C) 475 or (D) 496 genes. The p-value cutoff was set as 0.01. (E) GO enrichment analyses using SGD on the DEGs that were selected from the three comparative sample sets (*i*), (*ii*), and (*iii*), under GSH stress-induced conditions (250 μM GSH). The three sample sets include (*i*) HGT1 *RIM11*-K68A/HGT1 *RIM11*, (*ii*) HGT1 *RIM11*-K68A OE/HGT1 *RIM11*, and (*iii*) HGT1 *RIM11*-K68A OE/HGT1 *RIM11* OE ("strain" is omitted) (see Figure 5C). The upper panel shows the 23 upregulated genes and the lower panel shows the 41 down-regulated genes. P-value cutoff of 0.01 was adopted. (F) GO terms associated with 51 upregulated DEGs that were extracted from (*i*) and (*ii*), by using SGD with identical parameters (C, D and E). It should be noted that the four down-regulated genes seen in Figure 5C were not significantly categorized into any biological process at p-value cutoff < 0.01. The selected DEGs were dependent on (E) kinase activity of Rim11 or (F) protein abundance of Rim11. In the former category (E), most of the upregulated DEGs were categorized into ATP generation from ADP (GO:0006757), iron ion homeostasis (GO:0006826), and phospholipid biosynthetic process (GO:0008654) (see main text for details), with cluster frequency 65.3-fold (26.1%/0.40%) larger than that of the genome frequency. In the latter category (F), most of the upregulated genes were concentrated in the following GO terms, cytoplasmic translation (GO:0002181) or ribosome assembly (GO:0042255). Compared with the genome frequency, the ratio of the annotated genes was increased by 28.6-fold (80.0%/2.8%) for cytoplasmic translation and by 29.1-fold (32.0%/1.1%) for ribosome assembly, which implies that protein translation was preferentially enhanced. HGT1, plasma membrane-localized GSH transporter; GSH, reduced glutathione; RNA-seq, RNA sequencing; ATP, adenosine triphosphate; ADP, adenosine diphosphate.

Supplemental Figure 6

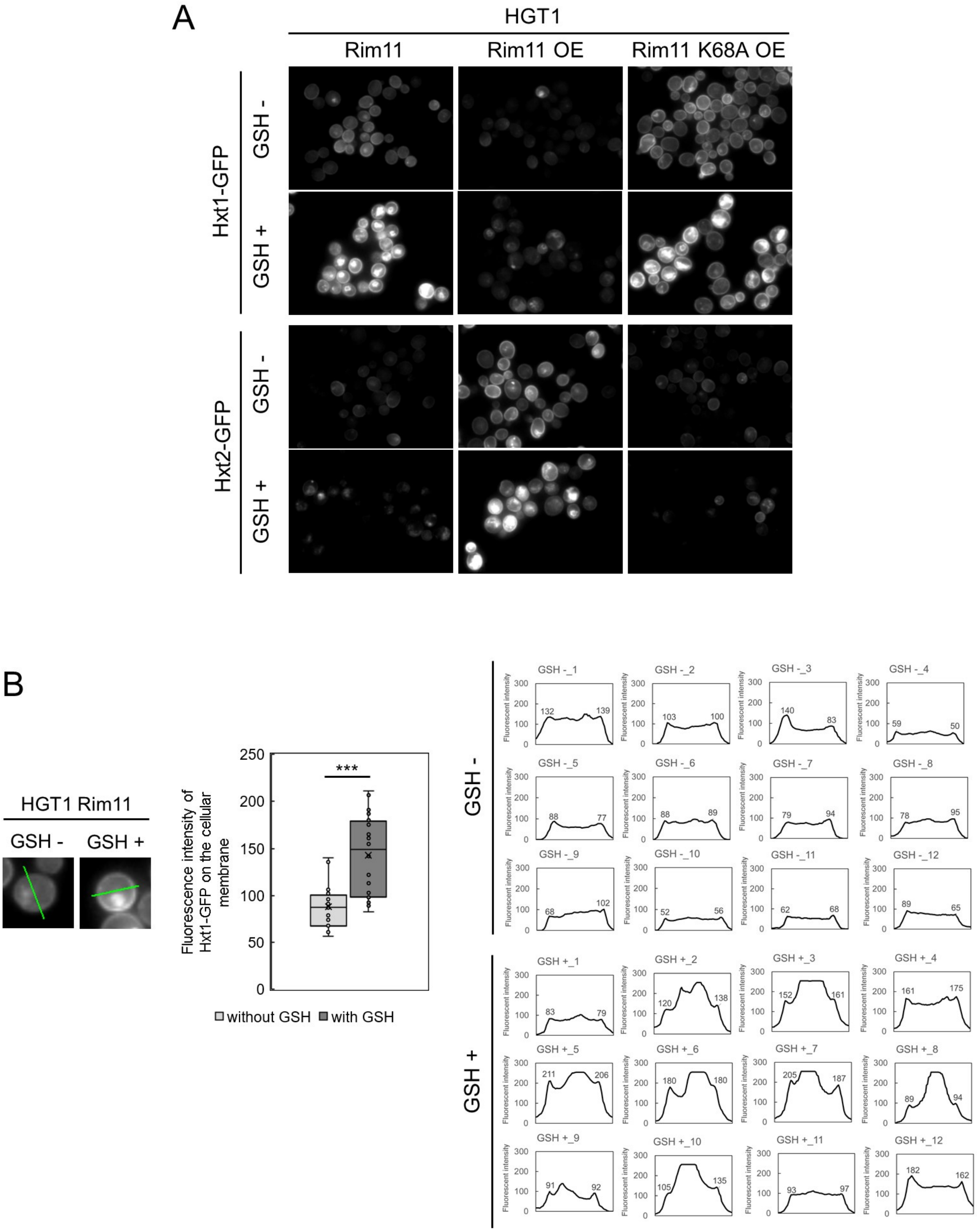

C

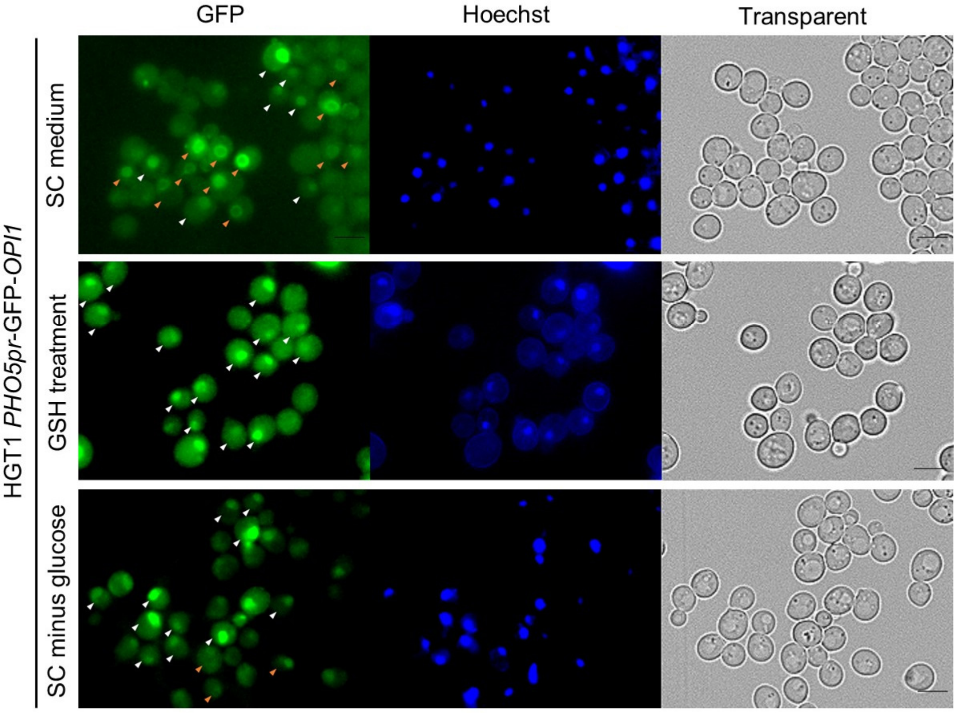
GFP
Hoechst
Transparent
HGT1 PHO5pr-GFP-OPI1
SC medium
GSH treatment
SC minus glucose

D

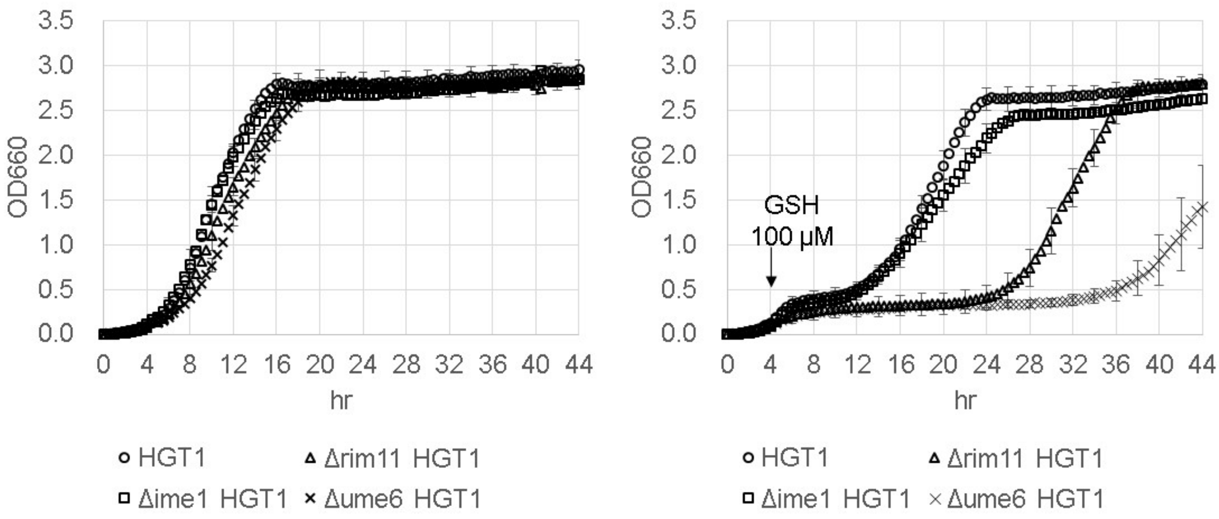
OD660
hr
GSH
100 μM
HGT1
Δrim11 HGT1
Δime1 HGT1
Δume6 HGT1

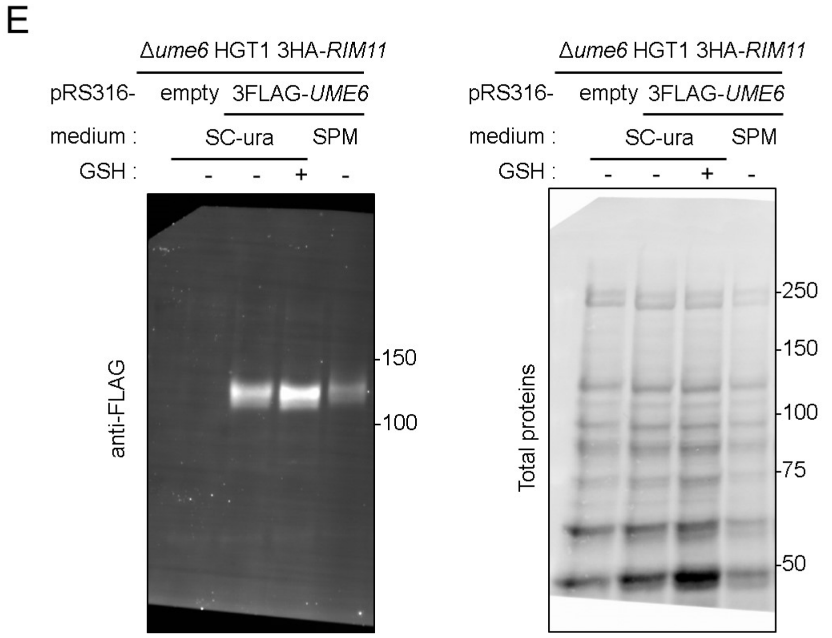

Figure S6. Biochemical analyses of *HXT1, HXT2, OPI1, IME1,* and *UME6* mutants in the HGT1 background. (A) Fluorescent images of Hxt1-GFP or Hxt2-GFP expressed in the HGT1 Rim11, HGT1 Rim11 OE, or HGT1 Rim11 K68A strains with or without GSH treatment. (B) The fluorescence intensity of Hxt1-GFP on the plasma membrane in HGT1 3HA-Rim11 strain, line-profile (right) and box plots (left) (n = 12 from three fields per sample, 2 dots in one sample). Brightness was quantified using BZ-X Analyzer software. The center line is the median, boxes are 25th and 75th percentiles, and whiskers are ±1.5 IQR. Significance test was done using two-tailed Welch's t-test, *** $p < 0.0001$. (C) Alternative images of the HGT1 *PHO5pr*-GFP-*OPI1* strain shown in main Figure 6B. Arrow head colored with orange and white shows GFP-Opi1 localized in ER and nucleus, respectively. The representative image is shown. Scale bar, 5 µm. (D) Growth curve analyses of the selected strains from Figure 6C in the main manuscript. The indicated strains were cultivated in SC at 30°C under non-stress conditions (left panel) or 100 µM GSH stress conditions (right panel). The mean and standard deviation were calculated from two independent experiments (n = 4). (E) Uncropped images of blots shown in Figure 6E in the main figure. GSH, reduced glutathione; HGT1, plasma membrane-localized GSH transporter; GFP: green fluorescent protein; OE, overexpressed; IQR, interquartile range; ER, endoplasmic reticulum.

Supplemental Figure 7

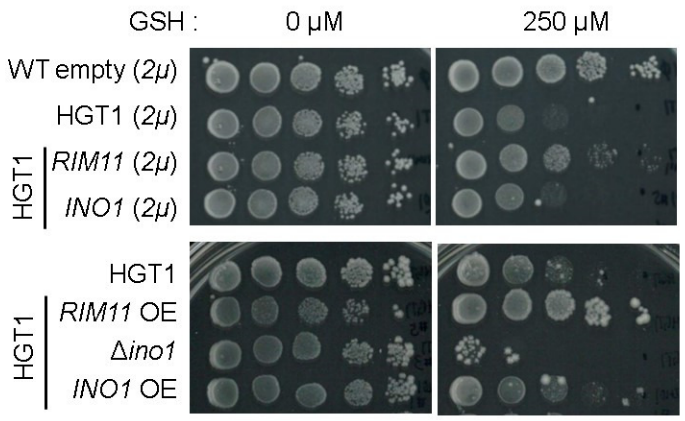

Figure S7. (A) Effects of altered *INO1* expression on the growth phenotype of the HGT1 strain under GSH stress-induced or -uninduced conditions. Upper image; HGT1 *INO1* (*2μ*), HGT1 strain harboring multicopy plasmid of pRS426-*INO1* (*2μ URA3*), and lower image; HGT1 *INO1* OE, HGT1 strain with a chromosomally integrated *INO1* expression cassette under the control of *TDH3* promoter. Each mutant strain grown in SC-ura (upper), or SC (lower) overnight was diluted and adjusted to an OD_{600} of 2.5 with distilled water, serially diluted 10-fold, and spotted onto SC-ura (upper) or SC (lower) agar plates with or without GSH, followed by incubation at 30°C. HGT1, plasma membrane-localized GSH transporter; GSH, reduced glutathione; OE, overexpressed; WT, wild-type.

Supplemental Table 1

Table S1. Chromosomal fragments obtained from plasma membrane-localized GSH transporter (HGT1) transformants with yeast chromosomal multicopy library.

Chromosome	Insert fragment (bp)	Genes
Chr V	543,995–550,410	*ECM32* (partial), *BMH1*, *PDA1*, *DMC1*, *ISC10*
Chr XIII	541,587–549,057	*GAT2* (partial), *POS2*, *CIN4*, *RIM11*, *SIP5* (partial)
Chr XV	402,582–412,365	*HIR2*, *CKB2*, *GLO4*, *CUE5*, *WHI2*

Supplemental Table 2

Table S2. RNA-seq results of *ULI1*, *MTH1,* hexose transporter genes, *HXT1,* and *HXT2* in the HGT1 strain and HGT1 derivatives with or without GSH stress induction.

Gene	(*i*)			(*ii*)			(*iii*)			(*iv*)			HGT1 strain GSH+ / GSH-		
	Log$_2$fc	FDR	Log$_{10}$ CPM	Log$_2$fc	FDR	Log$_{10}$ CPM	Log$_2$fc	FDR	Log$_{10}$ CPM	Log$_2$fc	FDR	Log$_{10}$ CPM	Log$_2$fc	FDR	Log$_{10}$ CPM
ULI1	-0.663	0.000	6.669	-1.29	0.000	6.467	-3.94	0.000	8.705	2.65	0.000	8.834	3.06	0.000	6.071
MTH1	-0.768	0.004	4.105	-0.678	0.003	4.146	-3.09	0.000	6.007	2.42	0.000	6.098	-2.14	0.000	5.716
HXT1	-0.169	0.237	9.853	-0.305	0.013	9.802	2.82	0.000	8.832	-3.12	0.000	9.108	0.56	0.000	9.586
HXT2	0.104	0.647	5.840	0.386	0.012	6.003	-3.41	0.000	8.722	3.80	0.000	8.699	0.26	0.020	5.573

Sample sets are as follows: (*i*) HGT1 *RIM11*-K68A/HGT1 *RIM11*, (*ii*) HGT1 *RIM11*-K68A OE/HGT1 *RIM11*, (*iii*) HGT1 *RIM11*-K68A OE/HGT1 *RIM11* OE, and (*iv*) HGT1 *RIM11* OE/HGT1 *RIM11*, respectively ("strain" is omitted). Biological function of each gene as described in the *Saccharomyces* Genome Database is mentioned.

ULI1: Protein of unknown function involved in and induced by the endoplasmic reticulum unfolded protein response (UPR).

MTH1: Negative regulator of the glucose-sensing signal transduction pathway; required for repression of transcription by Rgt1p; interacts with Rgt1p and the Snf3p and Rgt2p glucose sensors; phosphorylated by Yck1p, triggering Mth1p degradation.

HXT1: Low-affinity glucose transporter of the major facilitator superfamily; expression is induced by Hxk2p in the presence of glucose and repressed by Rgt1p when glucose is limiting.

HXT2: High-affinity glucose transporter of the major facilitator superfamily; expression is induced by low levels of glucose and repressed by high levels of glucose.

FDR: false discovery rate

CPM: counts per million

GSH, reduced glutathione

HGT1, plasma membrane-localized GSH transporter;

Supplemental Table 3

Table S3. Yeast strains used in this study.

Name	Genotype	Origin
BY4741	Mat**a** *his3Δ1 leu2Δ0 met15Δ0 ura3Δ0*	Lab stock
Δire1	Mat**a** *his3Δ1 leu2Δ0 met15Δ0 ura3Δ0 ire1::KanMX4*	Yeast knock-out strain collection (YKO; Horizon Discovery Ltd.)
Δrim11	Mat**a** *his3Δ1 leu2Δ0 met15Δ0 ura3Δ0 rim11::KanMX4*	YKO
Δbmh1	Mat**a** *his3Δ1 leu2Δ0 met15Δ0 ura3Δ0 bmh1::KanMX4*	YKO
Δwhi2	Mat**a** *his3Δ1 leu2Δ0 met15Δ0 ura3Δ0 whi2::KanMX4*	YKO
Δmck1	Mat**a** *his3Δ1 leu2Δ0 met15Δ0 ura3Δ0 mck1::KanMX4*	YKO
Δmrk1	Mat**a** *his3Δ1 leu2Δ0 met15Δ0 ura3Δ0 mrk1::KanMX4*	YKO
Δygk3	Mat**a** *his3Δ1 leu2Δ0 met15Δ0 ura3Δ0 ygk3::KanMX4*	YKO
Δino1	Mat**a** *his3Δ1 leu2Δ0 met15Δ0 ura3Δ0 ino1::KanMX4*	YKO
Δopi1	Mat**a** *his3Δ1 leu2Δ0 met15Δ0 ura3Δ0 opi1::KanMX4*	YKO
Δime1	Mat**a** *his3Δ1 leu2Δ0 met15Δ0 ura3Δ0 ime1::KanMX4*	YKO
Δume6	Mat**a** *his3Δ1 leu2Δ0 met15Δ0 ura3Δ0 ume6::KanMX4*	YKO
HGT1	Mat**a** *his3Δ1 leu2Δ0 met15Δ0 ura3Δ0 hgt1::LEU2-TDH3pr-HGT1*	This study
GFP-HGT1	Mat**a** *his3Δ1 leu2Δ0 met15Δ0 ura3Δ0 hgt1::LEU2-TDH3pr*-enhanced GFP [codon-optimized for *S. cerevisiae*]-*HGT1*	This study
Δrim11 HGT1	Mat**a** *his3Δ1 leu2Δ0 met15Δ0 ura3Δ0 rim11::KanMX4 hgt1::LEU2-TDH3pr-HGT1*	This study
HGT1 *ERO1*-6myc (*CEN*)	Mat**a** *his3Δ1 leu2Δ0 met15Δ0 ura3Δ0 hgt1::LEU2-TDH3pr-HGT1 pRS316-ERO1pr-ERO1-6 x* myc	This study
HGT1 *ERO1*-6myc (*2μ*)	Mat**a** *his3Δ1 leu2Δ0 met15Δ0 ura3Δ0 hgt1::LEU2-TDH3pr-HGT1 pRS426-ERO1pr-ERO1-6 x* myc	This study
HGT1 *RIM11* (*2μ*)	Mat**a** *his3Δ1 leu2Δ0 met15Δ0 ura3Δ0 hgt1::LEU2-TDH3pr-HGT1 pRS426-RIM11*	This study
HGT1 *MRK1* (*2μ*)	Mat**a** *his3Δ1 leu2Δ0 met15Δ0 ura3Δ0 hgt1::LEU2-TDH3pr-HGT1 pRS426-MRK1*	This study
HGT1 *MCK1* (*2μ*)	Mat**a** *his3Δ1 leu2Δ0 met15Δ0 ura3Δ0 hgt1::LEU2-TDH3pr-HGT1 pRS426-MCK1*	This study
HGT1 *YGK3* (*2μ*)	Mat**a** *his3Δ1 leu2Δ0 met15Δ0 ura3Δ0 hgt1::LEU2-TDH3pr-HGT1 pRS426-YGK3*	This study
HGT1 *INO1* (*2μ*)	Mat**a** *his3Δ1 leu2Δ0 met15Δ0 ura3Δ0 hgt1::LEU2-TDH3pr-HGT1 pRS426-INO1*	This study
Δbmh1 HGT1	Mat**a** *his3Δ1 leu2Δ0 met15Δ0 ura3Δ0 bmh1::KanMX4 hgt1::LEU2-TDH3pr-HGT1*	This study
Δwhi2 HGT1	Mat**a** *his3Δ1 leu2Δ0 met15Δ0 ura3Δ0 whi2::KanMX4 hgt1::LEU2-TDH3pr-HGT1*	This study
Δire1 HGT1	Mat**a** *his3Δ1 leu2Δ0 met15Δ0 ura3Δ0 ire1::KanMX4 hgt1::LEU2-TDH3pr-HGT1*	This study
Δire1 HGT1 *RIM11* (*2μ*)	Mat**a** *his3Δ1 leu2Δ0 met15Δ0 ura3Δ0 ire1::KanMX4 hgt1::LEU2-TDH3pr-HGT1 pRS426-RIM11*	This study
Δmck1 HGT1	Mat**a** *his3Δ1 leu2Δ0 met15Δ0 ura3Δ0 mck1::KanMX4 hgt1::LEU2-TDH3pr-HGT1*	This study
Δmrk1 HGT1	Mat**a** *his3Δ1 leu2Δ0 met15Δ0 ura3Δ0 mrk1::KanMX4 hgt1::LEU2-TDH3pr-HGT1*	This study
Δygk3 HGT1	Mat**a** *his3Δ1 leu2Δ0 met15Δ0 ura3Δ0 ygk3::KanMX4 hgt1::LEU2-TDH3pr-HGT1*	This study
Δino1 HGT1	Mat**a** *his3Δ1 leu2Δ0 met15Δ0 ura3Δ0 ino1::KanMX4 hgt1::LEU2-TDH3pr-HGT1*	This study
Δopi1 HGT1	Mat**a** *his3Δ1 leu2Δ0 met15Δ0 ura3Δ0 opi1::KanMX4 hgt1::LEU2-TDH3pr-HGT1*	This study
Δime1 HGT1	Mat**a** *his3Δ1 leu2Δ0 met15Δ0 ura3Δ0 ime1::KanMX4 hgt1::LEU2-TDH3pr-HGT1*	This study
Δume6 HGT1	Mat**a** *his3Δ1 leu2Δ0 met15Δ0 ura3Δ0 ume6::KanMX4 hgt1::LEU2-TDH3pr-HGT1*	This study
Δime1 HGT1 3HA-*RIM11* OE	Mat**a** *his3Δ1 leu2Δ0 met15Δ0 ura3Δ0 ime1::KanMX4 hgt1::LEU2-TDH3pr-HGT1 rim11::HIS3-pRS303-TDH3pr*-3 x HA-*RIM11*	This study
Δume6 HGT1 3HA-*RIM11* OE	Mat**a** *his3Δ1 leu2Δ0 met15Δ0 ura3Δ0 ume6::KanMX4 hgt1::LEU2-TDH3pr-HGT1 rim11::HIS3-pRS303-TDH3pr*-3 x HA-*RIM11*	This study
HGT1 *IRE1*-GFP	Mat**a** *his3Δ1 leu2Δ0 met15Δ0 ura3Δ0 hgt1::LEU2-TDH3pr-HGT1 ire1::URA3-pRS306-IRE1pr-IRE1*-enhanced GFP [codon-optimized for *S. cerevisiae*]	This study
Δrim11 HGT1 *IRE1*-GFP	Mat**a** *his3Δ1 leu2Δ0 met15Δ0 ura3Δ0 rim11::KanMX4 hgt1::LEU2-TDH3pr-HGT1*	This study

	ire1::URA3-pRS306-IRE1pr-IRE1-enhanced GFP [codon-optimized for *S. cerevisiae*]	
HGT1 GFP-*OPI1*	Mat**a** *his3Δ1 leu2Δ0 met15Δ0 ura3Δ0 hgt1::LEU2-TDH3pr-HGT1 opi1::URA3-pRS306-PHO5pr*-enhanced GFP [codon-optimized for *S. cerevisiae*]-*OPI1*	This study
HGT1 3HA-*RIM11*	Mat**a** *his3Δ1 leu2Δ0 met15Δ0 ura3Δ0 hgt1::LEU2-TDH3pr-HGT1 rim11::HIS3-pRS303-RIM11pr*-3 x HA-*RIM11*	This study
HGT1 3HA-*RIM11*-K68A	Mat**a** *his3Δ1 leu2Δ0 met15Δ0 ura3Δ0 hgt1::LEU2-TDH3pr-HGT1 rim11::HIS3-pRS303-RIM11pr*-3 x HA-*RIM11*^{K68A}	This study
HGT1 *RIM11* OE	Mat**a** *his3Δ1 leu2Δ0 met15Δ0 ura3Δ0 hgt1::LEU2-TDH3pr-HGT1 rim11::HIS3-pRS303-TDH3pr-RIM11*	This study
HGT1 3HA-*RIM11* OE	Mat**a** *his3Δ1 leu2Δ0 met15Δ0 ura3Δ0 hgt1::LEU2-TDH3pr-HGT1 rim11::HIS3-pRS303-TDH3pr*-3 x HA-*RIM11*	This study
HGT1 3HA-*RIM11*-K68A OE	Mat**a** *his3Δ1 leu2Δ0 met15Δ0 ura3Δ0 hgt1::LEU2-TDH3pr-HGT1 rim11::HIS3-pRS303-TDH3pr*-3 x HA-*RIM11*^{K68A}	This study
Δume6 HGT1 3HA-*RIM11* pRS316 [empty]	Mat**a** *his3Δ1 leu2Δ0 met15Δ0 ura3Δ0 ume6::KanMX4 hgt1::LEU2-TDH3pr-HGT1 rim11::HIS3-pRS303-RIM11pr*-3 x HA-*RIM11* pRS316 (*CEN URA3*) [empty]	This study
Δume6 HGT1 3HA-*RIM11* pRS316-3FLAG-*UME6*	Mat**a** *his3Δ1 leu2Δ0 met15Δ0 ura3Δ0 ume6::KanMX4 hgt1::LEU2-TDH3pr-HGT1 rim11::HIS3-pRS303-RIM11pr*-3 x HA-*RIM11* pRS316 (*CEN URA3*)-*UME6pr*-3 x FLAG-*UME6*	This study
Δrim11Δbmh1 HGT1	Mat**a** *his3Δ1 leu2Δ0 met15Δ0 ura3Δ0 rim11::KanMX4 bmh1::HIS3 hgt1::LEU2-TDH3pr-HGT1*	This study
Δrim11Δwhi2 HGT1	Mat**a** *his3Δ1 leu2Δ0 met15Δ0 ura3Δ0 rim11::KanMX4 whi2::URA3 hgt1::LEU2-TDH3pr-HGT1*	This study
Δbmh1Δwhi2 HGT1	Mat**a** *his3Δ1 leu2Δ0 met15Δ0 ura3Δ0 bmh1::KanMX4 whi2::URA3 hgt1::LEU2-TDH3pr-HGT1*	This study
Δrim11Δbmh1Δwhi2 HGT1 (*Δ3* HGT1)	Mat**a** *his3Δ1 leu2Δ0 met15Δ0 ura3Δ0 rim11::KanMX4 bmh1::HIS3 whi2::URA3 hgt1::LEU2-TDH3pr-HGT1*	This study
Δrim11Δmrk1 HGT1	Mat**a** *his3Δ1 leu2Δ0 met15Δ0 ura3Δ0 rim11::KanMX4 mrk1::HIS3 hgt1::LEU2-TDH3pr-HGT1*	This study
Δire1Δrim11 HGT1	Mat**a** *his3Δ1 leu2Δ0 met15Δ0 ura3Δ0 rim11::KanMX4 ire1::HIS3 hgt1::LEU2-TDH3pr-HGT1*	This study
Δino1Δrim11 HGT1	Mat**a** *his3Δ1 leu2Δ0 met15Δ0 ura3Δ0 rim11::KanMX4 ino1::HIS3 hgt1::LEU2-TDH3pr-HGT1*	This study
HGT1 *INO1* OE	Mat**a** *his3Δ1 leu2Δ0 met15Δ0 ura3Δ0 hgt1::LEU2-TDH3pr-HGT1 rim11::HIS3-pRS303-TDH3pr-INO1*	This study
Δrim11Δume6 HGT1	Mat**a** *his3Δ1 leu2Δ0 met15Δ0 ura3Δ0 rim11::KanMX4 ume6::URA3 hgt1::LEU2-TDH3pr-HGT1*	This study

Supplemental Table 4

Table S4. Plasmids used in this study.

Plasmid	Genotype	Origin
pBluescript II SK (+)	Standard cloning vector (pUC-type)	Lab stock
pRS303	Yeast integrating plasmid containing *HIS3* marker	Lab stock
pRS306	Yeast integrating plasmid containing *URA3* marker	Lab stock
pRS316	Low-copy yeast centromeric plasmid containing *URA3* marker	Lab stock
pRS426	Yeast multicopy plasmid containing *URA3* marker	Lab stock
p*HGT1*	pBluescript II SK (+) expressing *HGT1* from *TDH3* promoter and containing *LEU2* marker	This study
pGFP-*HGT1*	pBluescript II SK (+) expressing enhanced GFP [yeast-optimized]-*HGT1* from *TDH3* promoter and containing *LEU2* marker	This study
pRS316-*ERO1*-6myc	pRS316 expressing *ERO1*, whose C-terminus is fused with 6x myc	This study
pRS426-*ERO1*-6myc	pRS426 expressing *ERO1*, whose C-terminus is fused with 6x myc	This study
pRS426-*RIM11*	pRS426 expressing *RIM11* from native promoter	This study
pRS426-*BMH1*	pRS426 expressing *BMH1* from native promoter	This study
pRS426-*BMH2*	pRS426 expressing *BMH2* from native promoter	This study
pRS426-*WHI2*	pRS426 expressing *WHI2* from native promoter	This study
pRS316-*RIM11*	pRS316 expressing *RIM11* from native promoter	This study
pRS316-*CLB2pr-RIM11*	pRS316 expressing *RIM11* from *CLB2* promoter	This study
pRS316-*BMH1*	pRS316 expressing *BMH1* from native promoter	This study
pRS316-*WHI2*	pRS316 expressing *WHI2* from native promoter	This study
pRS426-*MCK1*	pRS426 expressing *MCK1* from native promoter	This study
pRS426-*YGK3*	pRS426 expressing *YGK3* from native promoter	This study
pRA426-*MRK1*	pRS426 expressing *MRK1* from native promoter	This study
p*RIM11pr*-3HA-*RIM11*	pRS303 expressing *RIM11* whose N-terminus is tagged with 3-repeat hemagglutinin (3 x HA), under the control of Rim11p's self-promoter	This study
p*TDH3pr*-3HA-*RIM11*	pRS303 expressing *RIM11* whose N-terminus is tagged with 3-repeat hemagglutinin (3 x HA), under the control of *TDH3* promoter	This study
p*RIM11pr*-3HA-*RIM11*^{K68A}	pRS303 expressing Rim11 (K68A) whose N-terminus is tagged with 3-repeat hemagglutinin (3 x HA), under the control of Rim11p's self-promoter	This study
p*TDH3pr*-3HA-*RIM11*^{K68A}	pRS303 expressing Rim11 (K68A) whose N-terminus is tagged with 3-repeat hemagglutinin (3 x HA), under the control of *TDH3* promoter	This study
p*IRE1*-GFP	pRS306 expressing *IRE1* (1 to 1,713 bp in ORF, domain I)-enhanced GFP [codon-optimized for *S. cerevisiae*]-*IRE1* (1,714 to 2,617 bp domain II) from Ire1's self-promoter	This study
pGFP-*OPI1*	pRS306 expressing *OPI1* whose N-terminus is tagged with enhanced GFP [codon-optimized for *S. cerevisiae*], under the control of *PHO5* promoter	This study
pRS316-3FLAG-*UME6*	pRS316 expressing *UME6*, N-terminus of which is fused with 3 repeat DYKDDDDK (FLAG) epitope tag, under control of the Ume6's self-promoter	This study
p*TDH3pr-INO1*	pRS303 expressing *INO1* under the control of *TDH3* promoter	This study
pGFP-*TDH3ter*-*URA3*	pBluescript II SK (+) carrying enhanced GFP [yeast-optimized], *TDH3* terminator region, and *URA3* marker	This study
p*HXT1*-GFP-*URA3-HXT1* (3-UTR)	pGFP-*TDH3ter-URA3* carrying ORF and 3'-UTR of *HXT1*	This study
p*HXT2*-GFP-*URA3-HXT2* (3-	pGFP-*TDH3ter-URA3* carrying ORF and 3'-UTR of *HXT2*	This study

UTR)		
p*INO1*-GFP-*URA3*-*INO1* (3-UTR)	pGFP-*TDH3ter*-*URA3* carrying ORF and 3'-UTR of *INO1*	This study

Supplemental Table 5

Table S5. Primer sets used in this study.

Name	Note	
p*HGT1*	AAGAGCTCCCATACAATTCTTTATCAAGCG	
	GGACTAGTTCTTTCTTCAACAACGATTGCT	
	CGGAATTCATGAGTACCATTTATAGGGAGA	
	CGGTCGACTGATTACCACCATTTATCATA	
pGFP-*HGT1*	GGGAACAAAAGCTGGAGCTCCCATACAATTCTTTATCAAGCG	
	CTTTAGACATGAATTCTTTGTTTGTTTATGTGTGTTTATTC	
	CGGGCTGCAGGAATTCATGTCTAAAGGTGAAGAATTATTCACTGG	
	TTTGTACAATTCATCCATACCATGGGTAA	
	GATGAATTGTACAAA*GGTGGT*AGTACCATTTATAGGGAGAGCGAC	Italicized sequence (*GGTGGT*) encodes Gly-Gly residues for linker sequence between GFP and Hgt1.
	CCCCCTCGAGGTCGACAACCATTTGCGACTGTCTTTTCAA	
p*RIM11pr (TDH3pr)*-3HA-*RIM11*	TATAGGGCGAATTGGAGCTCCAGTTCGAGTTTATCATTATC	
	TTTGTTTGTTTATGTGTGTTTATTC	
	ACATAAACAAACAAAATGAATATTCAAAGCAATAATTCTC	
	GCAGCCCGGGGGATCCTGGAGTACCTAAGATTTTAATG	
	GGCCGAATTCACTAAGTATTATCAGGAAAC	
	CCAAGTCGACTAATGCTATGTCAAGATCTT	
p*RIM11pr (TDH3pr)*-3HA-*RIM11*^{K68A}	GCTATT**GCC**AAAGTCCTGCAAGATAAA	GCC codes Ala residue.
	GACTTT**GGC**AATAGCAACTTTTTCATT	
p*IRE1*-GFP	TATAGGGCGAATTGGAGCTAAAACTCTGCTGCGCGCTG	
	CTCGCCCTTGCTCACAATTTTGGATAATAATACATATAGTGGCGGC	
	GTGAGCAAGGGCGAGGAGCTGTTCAC	
	CTTGTACAGCTCGTCCATGCCGAGAGTG	
	GACGAGCTGTACAAGGGATTTATGCCTGAAAAGGAAATCC	
	GCAGCCCGGGGGATCCTTCGACTTGGCACTGCAAA	
	ATTCGATATCAAGCTTGTCTTATCCTTGCCATAAACAG	
	CGGGCCCCCCCTCGAGAACCGGCATCTCTCAATTC	
pRS316-, pRS426-*RIM11*	TAGAACTAGTGGATCCGAGACGAAAATCGAATAGGA	
	CGGGCCCCCCCTCGAGTAATTCGAAGTGAAATGGCAT	
pRS316-*CLB2pr-RIM11*	TAGAACTAGTGGATCCTGTTCTTGACCGTCTACTAAG	
	CTATAAGATCAATGAAGAGAGAGAG	
	TCATTGATCTTATAGATGAATATTCAAAGCAATAATTCTCC	
	CGGGCCCCCCCTCGAGCCCTTTATACTCTAAAAACCTTG	
pRS316-, pRS426-*BMH1*	TAGAACTAGTGGATCCAGAAAAGGAGACCCCCTATG	
	CGGGCCCCCCCTCGAGGGGTGGAGGAATCAGAAG	
pRS426-*BMH2*	CGGGCCCCCCCTCGAGGTCACCCGGATCAGCAAA	
	TAGAACTAGTGGATCCGAAGAAAGAGGTGCGTATACAATG	
pRS316-, pRS426-*WHI2*	TAGAACTAGTGGATCCTTTACAGCATAGGCATAGTG	
	CGGGCCCCCCCTCGAGTTATACCGGATCAATGCTGC	
pRS426-*MCK1*	CGGGCCCCCCCTCGAGTCATTTTGTCTTCCCTCTTTC	
	TAGAACTAGTGGATCCATAACATGCGAGGCAATTTA	
pRS426-*YGK3*	CGGGCCCCCCCTCGAGGACAAAAAAGAAAGCAACTC	

	TAGAACTAGTGGATCCTGTTTTATGGATAGCCCAAAA	
pRS426-*MRK1*	CGGGCCCCCCCTCGAGTGAAGGAAAGGAAACCATATC	
	TAGAACTAGTGGATCCACCAAAAGGTTCAAAGATTAC	
pGFP-*OPI1*	TATAGGGCGAATTGGAGCTCTGTTGACCTGATGTCAGTC	
	TTCACCTTTAGACATTGGTAATCTCGAATTTGCTTG	
	ATGTCTAAAGGTGAAGAATTATTC	
	*AGCACC*TTTGTACAATTCATCCATACCA	Italics code Gly-Ala residues for linker sequence between GFP and Opi1.
	TTGTACAAAGGTGCTTCTGAAAATCAACGTTTAGGA	
	ATCGATAAGCTTTTCTTTTACCTCCTGTTTCTG	
	AAGCTTATCGATAGAGAAATCGAATTTATACCGC	
	CGGGCCCCCCCTCGAGTTGTTTGTGCTATGTTCTCG	
pRS316-3FLAG-*UME6*	TAGAACTAGTGGATCCATTGTTGGGTTTAACTCTTCA	
	GATGTCATGATCTTTATAATCACCGTCATGGTCTTTGTAGTCCATCGTGAGTTAAGGTCC	
	TAAAGATCATGACATCGATTACAAGGATGACGATGACAAGCTCCTAGACAAGGCGCGCTCTC	
	CGGGCCCCCCCTCGAGGGCAGAGCGAAGCTTACTCA	
p*TDH3pr-INO1*	CGGGCTGCAGGAATTCAACGGGGGAAAAGGTCTC	
	CCCCCTCGAGGTCGACTTTTCACATGCCGCATTTAGC	
	ACATAAACAAACAAAAATGACAGAAGATAATATTGCTC	
	GCAGCCCGGGGGATCCATTAACACCAGGAGATACTTC	
pRS426-*INO1*	CGGGCCCCCCCTCGAGTGATCGGAACGAGCTCTTTA	
	TAGAACTAGTGGATCCTTGAGTGGTCGCACATACAC	
pGFP-*TDH3ter-URA3*	CCCCCTCGAGGTCGACTCTAAAGGTGAAGAATTATTCACTGG	
	TTATTTGTACAATTCATCCATACCATG	
	GAATTGTACAAATAAGTGAATTTACTTTAAATCTTGCATTTAAA	
	AGGGAAAGATATGAGCTATACAG	
	CTCATATCTTTCCCTCTTAACTATGCGGCATCAGAG	
	CGGGCTGCAGGAATTCCCTGATGCGGTATTTTCTCC	
p*HXT1*-GFP-*URA3-HXT1* (3-UTR)	CGGGCCCCCCCTCGAGCAACGTACTATGATGGGTATCATG	
	ACCTTTAGA*ACCACC*TTTCCTGCTAAACAAACTCTTGTAAA	Italics code Gly-Gly residues for linker sequence between Hxt1 and GFP.
	*GGTGGT*TCTAAAGGTGAAGAATTATTCACTGG	
	CCTGATGCGGTATTTTCTCC	
	AAATACCGCATCAGGGAAATTGACAACGCTGCTG	
	ACCGCGGTGGCGGCCGCGCTAAACCTCAAAGTTGAAACTAAAG	
p*HXT2*-GFP-*URA3-HXT2* (3-UTR)	CGGGCCCCCCCTCGAGACCAAAACACATTAGAGGTACCTG	
	ACCTTTAGA*ACCACC*TTCCTCGGAAACTCTTTTTTCTTTTG	Italics code Gly-Gly residues for linker sequence between Hxt2 and GFP.
	*GGTGGT*TCTAAAGGTGAAGAATTATTCACTGG	
	CCTGATGCGGTATTTTCTCC	
	AAATACCGCATCAGGCAGCGAAATGATTGCATTTACTG	
	ACCGCGGTGGCGGCCGCTGGTATCCGTTTTATTGGCCA	
p*INO1*-GFP-*URA3-INO1* (3-UTR)	CGGGCCCCCCCTCGAGGGACATCAATAACGCAGATCTATAC	
	ACCTTTAGA*ACCACC*CAACAATCTCTCTTCGAATCTTAGTTC	Italics code Gly-Gly residues for linker sequence between Ino1 and GFP.
	*GGTGGT*TCTAAAGGTGAAGAATTATTCACTGG	
	CCTGATGCGGTATTTTCTCC	

	AAATACCGCATCAGGGAACTATTGCCTCTTTGTCACTTC	
	ACCGCGGTGGCGGCCGCAAAGTGAGATAGCTGGTCCC	

Bold, underline, and italics show specific point for introduction of site-directed mutagenesis, overlap regions, and linker sequences, respectively.

Supplemental Table 6

Table S6. Raw data of RNA-seq performed in this study.

Sample name	Raw Reads Number	Raw Bases Number	Clean Reads Number	Clean Reads Rate (%)	Clean Bases Number	Low-quality Reads Number	Low-quality Reads Rate (%)	Ns Reads Number	Ns Reads Rate (%)	Adapter Polluted Reads Number	Adapter Polluted Reads Rate (%)	Raw Q30 Bases Rate (%)	Clean Q30 Bases Rate (%)
RIM11pr-RIM11_NT_GSH-	16,461,018	2,469,152,700	15,013,848	91.21	2,252,077,200	58,238	0.35	650	0.00	1,388,282	8.43	94.51	94.78
RIM11pr-RIM11_NT_GSH-	16,598,566	2,489,784,900	14,750,268	88.86	2,212,540,200	76,050	0.46	842	0.01	1,771,406	10.67	93.96	94.42
RIM11pr-RIM11_NT_GSH-	16,685,206	2,502,780,900	15,329,244	91.87	2,299,386,600	76,682	0.46	554	0.00	1,278,726	7.66	93.90	94.28
RIM11pr-RIM11_NT_GSH+	15,814,160	2,372,124,000	14,678,928	92.82	2,201,839,200	65,634	0.41	626	0.00	1,068,972	6.76	93.92	94.40
RIM11pr-RIM11_NT_GSH+	16,650,906	2,497,635,900	15,780,942	94.78	2,367,141,300	86,232	0.52	724	0.00	783,008	4.70	93.80	94.15
RIM11pr-RIM11_NT_GSH+	15,355,830	2,303,374,500	14,705,036	95.76	2,205,755,400	75,718	0.49	608	0.00	574,468	3.74	93.81	94.12
RIM11pr-RIM11_K	16,587,318	2,488,097,700	15,836,868	95.48	2,375,530,200	78,024	0.47	668	0.00	671,758	4.05	93.98	94.32

68A_GSH-													
RIM11pr-RIM11_K68A_GSH-	15,942,988	2,391,448,200	14,727,918	92.38	2,209,187,700	66,592	0.42	584	0.00	1,147,894	7.20	94.03	94.45
RIM11pr-RIM11_K68A_GSH-	16,156,480	2,423,472,000	15,026,958	93.01	2,254,043,700	84,232	0.52	610	0.00	1,044,680	6.47	93.48	94.23
RIM11pr-RIM11_K68A_GSH+	16,438,216	2,465,732,400	15,165,082	92.25	2,274,762,300	65,450	0.4	694	0.00	1,206,990	7.34	93.60	94.34
RIM11pr-RIM11_K68A_GSH+	17,098,244	2,564,736,600	15,600,030	91.24	2,340,004,500	71,090	0.42	716	0.00	1,426,408	8.34	94.00	94.34
RIM11pr-RIM11_K68A_GSH+	15,828,646	2,374,296,900	14,795,632	93.47	2,219,344,800	70,608	0.45	628	0.00	961,778	6.08	94.10	94.39
TDH3pr-RIM11_NT_GSH-	15,765,276	2,364,791,400	14,934,910	94.73	2,240,236,500	87,654	0.56	674	0.00	742,038	4.71	93.76	94.08

TDH3pr-RIM11_NT_GSH-	16,527,148	2,479,072,200	15,541,986	94.04	2,331,297,900	73,268	0.44	778	0.01	911,116	5.51	93.76	94.43
TDH3pr-RIM11_NT_GSH-	16,731,420	2,509,713,000	15,843,870	94.69	2,376,580,500	85,732	0.51	632	0.00	801,186	4.79	93.67	94.33
TDH3pr-RIM11_NT_GSH+	16,000,794	2,400,119,100	14,740,892	92.13	2,211,133,800	66,020	0.41	592	0.00	1,193,290	7.46	93.93	94.44
TDH3pr-RIM11_NT_GSH+	16,613,756	2,492,063,400	15,571,050	93.72	2,335,657,500	67,834	0.41	734	0.00	974,138	5.86	94.27	94.64
TDH3pr-RIM11_NT_GSH+	17,433,336	2,615,000,400	15,746,052	90.32	2,361,907,800	86,012	0.49	894	0.01	1,600,378	9.18	93.84	94.23
TDH3pr-RIM11_K68A_GSH-	15,711,786	2,356,767,900	14,711,482	93.63	2,206,722,300	70,142	0.45	566	0.00	929,596	5.92	93.95	94.35
TDH3pr-RIM11_K68A_GSH-	16,609,896	2,491,484,400	15,428,106	92.89	2,314,215,900	81,160	0.49	734	0.00	1,099,896	6.62	93.80	94.38
TDH3pr-RIM11_K68A_GSH-	15,526,860	2,329,029,000	14,714,260	94.77	2,207,139,000	70,680	0.46	646	0.00	741,274	4.77	94.03	94.44

TDH3pr-RIM11_K68A_GSH+	16,239,544	2,435,931,600	15,347,244	94.5	2,302,086,600	75,130	0.46	896	0.01	816,274	5.03	94.10	94.47
TDH3pr-RIM11_K68A_GSH+	15,541,060	2,331,159,000	14,824,364	95.39	2,223,654,600	67,484	0.43	800	0.01	648,412	4.17	94.08	94.42
TDH3pr-RIM11_K68A_GSH+	16,503,642	2,475,546,300	15,537,648	94.15	2,330,647,200	69,536	0.42	664	0.00	895,794	5.43	94.10	94.39
