# Supplementary material for: Yeast Rim11 kinase responds to glutathione-induced stress by regulating the transcription of phospholipid biosynthetic genes
Source: Mol Biol Cell. 2023 Dec 14;35(1):ar8. doi: 10.1091/mbc.E23-03-0116 (PMC10881166; doi:10.1091/mbc.E23-03-0116)
Supplement: Supplementary file 2 [file mbc-35-ar8-s002.pdf]

Yeast Rim11 kinase responds to glutathione-induced stress by regulating the transcription of phospholipid biosynthetic genes

Taishi Yasukawa, Ryo Iwama, Yuriko Yamasaki, Naohisa Masuo, and Yoichi Noda

Corresponding author(s): Yoichi Noda, The University of Tokyo

Review Timeline:

Submission Date:	2023-03-30
Editorial Decision:	2023-04-26
Revision Received:	2023-09-29
Editorial Decision:	2023-10-17
Revision Received:	2023-10-22
Accepted:	2023-10-27

Editor-in-Chief: Matthew Welch

Transaction Report:

(Note: With the exception of the correction of typographical or spelling errors that could be a source of ambiguity, letters and reports are not edited. The original formatting of letters and referee reports may not be reflected in this compilation.)

RE: Manuscript #E23-03-0116
TITLE: Yeast Rim11 kinase responds to glutathione-induced stress by regulating the transcription of phospholipid biosynthetic genes

Dear Dr. Noda:

Thank you for submitting your work to MBoC. Both reviewers were supportive of publishing your study, with a number of revisions required. Reviewer 1 has extensive suggestions as to how your findings fit well with previous work that is relevant and might be incorporated into your analysis, and especially into your model. Reviewer 2 has a number of experimental suggestions that I agree would strengthen the study, but also some concerns that can be addressed textually. I personally disagree with this reviewer that RNA-seq data needs to be confirmed by RT-PCR, so I would not expect you to follow up with this unless you happen to have data in hand. If you'd like to submit a revision plan, I'd be happy to evaluate that to help advise you.

Sincerely,

Elizabeth Miller
Monitoring Editor
Molecular Biology of the Cell

Dear Dr. Noda,

The review of your manuscript, referenced above, is now complete. The Monitoring Editor has decided that your manuscript is not acceptable for publication at this time, but may be deemed acceptable after specific revisions are made, as described in the Monitoring Editor's decision letter above and the reviewer comments below.

A reminder: Please do not contact the Monitoring Editor directly regarding your manuscript. If you have any questions regarding the review process or the decision, please contact the MBoC Editorial Office (mboc@ascb.org).

When submitting your revision include a rebuttal letter that details, point-by-point, how the Monitoring Editor's and reviewers' comments have been addressed. (The file type for this letter must be "rebuttal letter"; do not include your response to the Monitoring Editor and reviewers in a "cover letter.") Please bear in mind that your rebuttal letter will be published with your paper if it is accepted, unless you have opted out of publishing the review history.

Authors are allowed 90 days to submit a revision. If this time period is inadequate, please contact us at mboc@ascb.org.

Revised manuscripts are assigned to the original Monitoring Editor whenever possible. However, special circumstances may preclude this. Also, revised manuscripts are often sent out for re-review, usually to the original reviewers when possible. The Monitoring Editor may solicit additional reviews if it is deemed necessary to render a completely informed decision.

In preparing your revised manuscript, please follow the instruction in the Information for Authors (www.molbiolcell.org/info-for-authors). In particular, to prepare for the possible acceptance of your revised manuscript, submit final, publication-quality figures with your revision as described.

To submit the rebuttal letter, revised manuscript, and figures, use this link: Link Not Available

MBoC offers the option to publish your work with immediate open access. Open access can increase the discoverability and usability of your research. If you would like to publish your paper with immediate open access but did not select this option during initial submission, please contact the editorial office at mbc@ascb.org.

Thank you for submitting your manuscript to MBoC. We look forward to receiving your revised paper.

Sincerely,

Eric Baker
Journal Production Manager
MBoC Editorial Office
mbc@ascb.org

Reviewer #1 (Remarks to the Author):

See attached.

Reviewer #2 (Remarks to the Author):

Yasukawa et al performed a screen designed to isolate genes that modulate the growth defect in S. cerevisiae associated with the uptake of high level of glutathione from the medium (facilitated by high-level expression of the plasma membrane glutathione transporter). They describe the isolation of three multicopy suppressors (RIM11, BHM1, and WHI2). Initially the authors focus on how loss or overexpression of these three genes impacts the ability of cells to survive under conditions of increased glutathione uptake. Later, the authors focus on Rim11 and explore how Rim11 kinase activity alters transcriptional profiling connected with the exposure to GSH. The authors propose a model for the upregulation of a subset of genes involved in lipid biogenesis that is dependent on Rim11 kinase activity and occurs in response to the cellular uptake of exogenous glutathione. The authors go on to make a series of observations about various players connected to Rim11 signaling and/or the transcription of lipid biogenesis genes, including OPI1, UME6, IME1, and INO1. They report transcriptional changes observed in the presence of GSH, as well as growth differences in the presence, absence, or overexpression of these genes.

Overall, the manuscript contains several interesting observations that suggest unexpected signaling activated in the presence of exogenous GSH, a less explored "stressor". Yet, in its current form, the manuscript feels incomplete in its goal to outline a clear Rim11-dependent signaling output that is associated with GSH uptake. While the authors outline a model in Figure 7, this model relies heavily on relationships anticipated based on prior studies focused on the signaling outlined in a variety of conditions, not necessarily connections established by the authors to occur as part of GSH-induced signaling. In broad terms, it feels like a significant jump from the reported data to the model of Figure 7, including proteins in the model that are not discussed or explored in the manuscript. The extent of the experiments do not seem to match with the overall conclusions and model.

Some suggestions as to further studies that may help to focus or complete the study:

1. Arguably, the manuscript could benefit from some reorganization, with a clear focus from the initial results on Rim11. For example, reporting the initial isolation of RIM11 as a high-copy suppressor, and a straightforward analysis of whether overexpression of RIM11 requires Ire1 to confer resistance to GSH in the HGT1 strain, would streamline the results. These straightforward experiments would establish a role for Rim11, and a role that is outside the prior focus on the UPR that the Toledano work reported in Kumar et al 2011.

2. As the manuscript is currently setup, the authors make epistasis arguments about the three initially isolated suppressors from the spotting assay in Figure 1E. These conclusions appear to be a potential overinterpretation of the data. It is also unclear what the pathway organization (if true) means in the context of the later analysis of, and focus on, Rim11.

The growth defects observed between the three individual mutants on plates are modest and seem variable (Fig 1D the bhm1Δ and whi2Δ growth are called similar, versus in Fig 1E the bmh1Δ shows better growth than the whi2Δ). This growth variability makes the argument as to whether there is a true additive impact, or if the difference in growth is just assay-to-assay variation, difficult to discern. Similarly, the growth suppression of the null strains with the various 2µ plasmids is suggestive, but not particularly robust or fully consistent with the outlined model. It is unclear in the outlined epistasis diagram why a bhm1Δ would not be bypassed by additional RIM11 (?) If the authors do want to make arguments about epistasis, some evidence of reproducibility is also warranted, including how many replicates were performed and/or showing replicates of the strains.

3. The authors do not validate the transcriptional upregulation they outline for their RNA-seq dataset. Some validation by RT-PCR would strengthen the conclusions. It would also allow one to establish if Rim11 is required for the upregulation (e.g., analysis in a rim11Δ). Such data would complement the focus on growth as the major determinant for a signaling role.

4. The experiments of Figure 6 exploring Opi1, Ime1, Ume6 and Ino1 expression or localization during conditions of increased GSH are suggestive of unique roles. However, these data could be more robust. The variable expression of Opi1 in the "GSH stress" cells makes the co-localization data difficult to parse. It is not clear why a more intense western band for Ume6-FLAG implies an increased in "Ume6 phosphorylation".

5. The authors observe and say that the degree and/or location of phosphorylation on Rim11 is likely distinct for GSH and glucose starvation, but yet then the authors argue that GSH induces a glucose starvation response (in the discussion). If the authors wish to imply that glucose transporters, etc. are important players in the response to GSH (as discussed), then some experiments to this point should be performed. Or at a minimum, the transcriptional signature that the authors use in the discussion to support a glucose starvation response should be more clearly outlined in the results.

6. The RNA-seq were completed after 2 h exposure to 250 µM GSH. The authors state that these data are "consistent with the

results reported previously" of Kumar et al 2011. It seems a missed opportunity for a more descriptive comparison. Given the prior work followed the transcriptional response at an earlier and lower dose (50 µM GSH for 30 min), one would expect some differences.

7. Minor point. The authors convincingly establish that HGT1 overexpression confers sensitivity to exogenous GSH as reported previously. Yet, it is unclear what the % quantification of GSH (8-9% intracellular GSH concentration) refers to in Figure 1B. One presumes this is 8-9% of the total GSH added to the culture?

Reviewer 1

In this work, the authors examined the effects of high levels of glutathione (GSH) on the stress response in yeast. Through a genomic screen, the RIM11, BMH1, and WHI2 genes were identified as high copy number suppressors of the growth inhibition caused by GSH stress. Strains carrying mutations in each of these genes were sensitive to GSH. The study then focused on Rim11, the yeast homolog of glycogen synthase kinase 3beta. This protein kinase, which is subject to autophosphorylation, has been associated with the phosphorylation of transcription factors involved with meiosis, and a recent paper revealed that Rim11 regulates the partitioning of the lipid intermediate phosphatidate (PA) between the synthesis of membrane phospholipids and the neutral lipid triacylglycerol via the phosphorylation and inhibition of the PA phosphatase activity encoded by PAH1 (Khondker et al. (2022). Here, the authors showed through an RNA-seq analysis that RNA levels of UAS_{INO}*-containing phospholipid biosynthetic genes (e.g., INO1, CHO1, CHO2, OPI3, etc.) were repressed under GSH stress, whereas multiple copies of RIM11 caused the derepression of these genes. The Rim11-mediated effects of the gene expression were dependent on its protein kinase activity. The work also demonstrated that GSH invokes multiple stress-responsive pathways, including a part of the adaptive pathway to glucose limitation.*

Overall, major conclusions are supported by a sound experimental approach. The data indicate that Rim11 protein kinase activity regulates transcription of UAS_{INO}*-containing phospholipid synthesis genes via the Henry (Opi1/Ino2-Ino4) regulatory circuit in response to GSH stress. Major shortcomings of the work, however, are that (1) expression of the encoded protein/phospholipid synthesizing activity of at least one gene identified by the RNA-seq analysis be confirmed and that (2) the cellular phospholipid content be correlated with the Rim11-mediated expression of the* UAS_{INO}*-containing genes.*

Response: Thank you for these helpful suggestions. We have included several new experiments to address the points raised, including points (1) and (2) that are mentioned above. We have addressed each of the specific comments below.

Although the title "Yeast Rim11 kinase responds to glutathione-induced stress by regulating the transcription of phospholipid biosynthetic genes," the Introduction and Discussion pay little attention to the wealth of information about lipid synthesis, especially with regard to how the findings of this work relates to what is known about the Rim11-mediated regulation of lipid synthesis via the phosphorylation of Pah1. Pah1 is the PA phosphatase enzyme that regulates the levels of its substrate PA, which in turn governs the location and repressor function of Opi1. The authors show that Opi1 location is regulated by GSH stress and Rim11 protein kinase activity, but don't draw the

connection between the Rim11- mediated phosphorylation of Pah1, the inhibition of its PA phosphatase activity, and regulation of lipid synthesis. Instead they focus on hypothetical scenarios for which clear cut evidence is missing (see specific comments below).

Khondker et al. (2022) (briefly mentioned in passing at the end of the Discussion) showed that Rim11, expressed and purified from yeast where it is subject to its autophosphorylation, phosphorylates Pah1 on multiple residues (majorly Thr-163, Thr-164, and Ser-602). Pah1 is the PA phosphatase that dephosphorylates PA to produce diacylglycerol, which is then acylated to form triacylglycerol. Mutants lacking PA phosphatase activity accumulate PA, which cause the retention of Opi1 outside the nucleus resulting in the derepression of the UAS_{INO}*-containing genes (especially CHO1 (ref 15 in Khondker et al 2022) and an increase in the synthesis of phospholipids (Also see ref. 8 in Khondker et al. for a recent review). The Rim11-mediated phosphorylation of Pah1 inhibits PA phosphatase activity. The inhibition of PA phosphatase activity by Rim11 would cause the accumulation of the substrate PA, the retention of Opi1 outside the nucleus, and the derepression of* UAS_{INO}*-containing genes. Khondker et al (2022) also showed that cells expressing phosphorylation-deficient forms of Pah1 (T163A/T164A and S602A) exhibit an increase in the synthesis of triacylglycerol when Pah1 cannot be dephosphorylated by the Nem1-Spo7 complex. This is consistent with Pah1-mediated reduction in PA, translocation of Opi1 into the nucleus for repression of* UAS_{INO}*-containing genes and increase in the synthesis of triacylglycerol at the expense of membrane phospholipids. Khondker et al. (2022) did not perform experiments that make the direct connection between Rim11 protein kinase activity and the transcription of* UAS_{INO}*-containing genes, which is demonstrated in this study! Fig. 7 can be revised to show this connection.*

Response: Thank you for these helpful comments. We have addressed the points raised by clarifying the connections between GSH stress, functions of Rim11 and Pah1, and the change in Opi1 localization in a new model figure (Figure 8) in the revised manuscript. We have also included descriptions in the main text to explain the model in detail (see below, this letter Page 3, line 1 – 29).

Overall, the impact of this work with respect to lipid synthesis is high, but the authors are not seeing it because they are generally unaware of the crucial role Pah1 PA phosphatase plays in the regulation of PA levels and lipid synthesis, and that PA phosphatase is regulated by phosphorylation via Rim11.

Response: Regarding the relationship between the GSH stress response and phospholipid biosynthesis, we have added the following sentences in the Discussion. We have comprehensively discussed the relationships between GSH treatment, Rim11, and Pah1.

(Page 14; line 20–27, in the revised manuscript)

"Figure 8A shows the function of Rim11 in the GSH stress response. Khondker et al. (2022) reported that Rim11 phosphorylates phosphatidic acid phosphatase (Pah1), and thereby inhibits its phosphatase activity. Pah1 is dephosphorylated by the Nem1-Spo7 complex and is subsequently recruited to the nuclear/ER membranes where it performs its functions (Karanasios et al., 2010; Choi, et al., 2011). At the membrane, Pah1 dephosphorylates phosphatidic acid and converts it into DAG. This regulates the localization and function of Opi1, which is tethered to the ER membrane via interaction with PA and the ER membrane protein, Scs2 (Loewen et al., 2004). Therefore, it is likely that the kinase activity of Rim11 and the function of Pah1 play an important role in the recovery of phospholipid biosynthetic gene expression levels in the GSH stress response."

(Page 14 line 34–39, in the revised manuscript)

"At the protein level, Ino1-GFP was significantly decreased upon GSH treatment (Figure 7A). Moreover, PC, PE, and PS contents were decreased upon GSH treatment (Figure 7D). We hypothesize that Rim11 translocates into the nucleus following GSH treatment (Figure 4C, Supplemental Fig. 4C), and phosphorylation of Pah1 by Rim11 is decreased. We predict that reactivated Pah1 converts PA to DAG, which leads to the reduction of PA levels and translocation of Opi1 into the nucleus, ultimately leading to repression of the transcription of the phospholipid biosynthetic gene (Figure 8B)."

(Page 15 line 5–11, in the revised manuscript)

"The amount of Ino1-GFP was consistent with the changes in the *INO1* mRNA level (Figure 7A). Moreover, lipidomic analyses revealed that overexpression of *RIM11* increased the overall lipid contents in the cell (Figure 7D). Therefore, we hypothesized that de-repression of the decreased biosynthetic activity of phospholipids by Rim11, and the increased expression of the *INO1* gene, may contribute to enhanced tolerance of the cells to GSH. Specifically, overexpressed Rim11 phosphorylates Pah1, thereby inhibiting the dephosphorylating activity of Pah1. We hypothesize that the proportion of Opi1, which is anchored on the ER membrane, then increases, causing transcriptional de-repression of the phospholipid biosynthetic genes."

Specific comments:

Page 5 Line 30- the authors do not propose an explanation for why RIM11 under its native promoter did not rescue the growth phenotype.

Response: We deduced that one of the possibilities was that the original promoter region was not sufficiently long. Therefore, a longer 5’-region was subcloned as a promoter of *RIM11*, and recovery of the GSH sensitivity was assessed. As shown in Figure for the reviewers A, phenotypic recovery

was partial even with the longer 5'-region, suggesting that some other elements on the original plasmid obtained by the high-copy suppressor screening may be needed for sufficient expression of the *RIM11* gene. Alternatively, as *RIM11* expression increases in the stationary phase (Gasch *et al*., 2000), we speculate that it was only insufficiently expressed under the experimental conditions shown in Figure 1D. However, as this result does not seem to affect the main findings described in this manuscript, we only added a brief explanation to the main text.
We have added the following sentences in the main text:

(Page 5; line 34–38, in the revised manuscript) "Although we also tested the long *RIM11* 5'-region as a promoter, no increase in growth phenotype rescue was observed (data not shown). It is possible that other elements present on the original plasmid obtained by the high-copy suppressor screening were needed for sufficient expression of the *RIM11* gene. Alternatively, as expression of *RIM11* increases in the stationary phase (Gasch *et al*., 2000), *RIM11* may be insufficiently expressed by its own promoter under the conditions shown in Figure 1D."

Figure 1E- bmh1Δ and whi2Δ show a synthetic growth defect when combined, which is in conflict with the proposed model in Figure 1F that shows them in the same pathway.
Response: We agree that there are some inconsistencies between the results and the model. We carefully reconsidered the results of the spot assays and concluded that it was difficult to create a diagram that reflected all the data correctly. Therefore, we have removed the diagram from the revised manuscript. Please also see our response to the next point.

Figure 1F- There is no clear explanation in the text as to why there is no genetic interaction between Rim11 and Whi2. The proposed model also doesn't explain why overexpression of Rim11 rescues the growth defect in whi2Δ but not bmh1Δ, nor why Whi2 overexpression rescues rim11Δ.
Response: We believe that genetic interaction occurred between rim11 and whi2. As shown in Figure 1E, the Δ*rim11* Δ*whi2* strain showed higher GSH sensitivity than did the Δ*whi2* strain. Moreover, as shown in Figure 1F, overexpression of *RIM11* partially rescued GSH sensitivity of the Δ*whi2* strain, and vice versa. Overexpression of *WHI2* partially rescued GSH sensitivity of the Δ*rim11* strain. As we mentioned above and as suggested by you, we concluded that it was difficult to create a diagram that reflected all the data correctly. Therefore, we have removed the diagram from the revised manuscript. To explain the results more clearly and accurately than in the original manuscript, we have revised the main text as follows:

(Page 5–6, line 33–1, in the original manuscript)
"We further examined the GSH stress sensitivity of double or triple mutants of these three genes

(Figure 1E). The Δ*bmh1*Δ*whi2* and Δ*rim11*Δ*bmh1* HGT1 strains showed augmented GSH stress sensitivity compared with their single-deletion mutants, and the latter strain showed less severe additive effects, indicating the existence of genetic interactions between *BMH1* and *WHI2*, or *BMH1* and *RIM11*. To investigate whether functional interactions exist among these three genes in the GSH stress response pathway, each single-deletion mutant was transformed with other suppressors (Figure 1F). The results suggested that *BMH1* is located upstream of *WHI2* and showed that loss of one of the two genes markedly decreased GSH stress tolerance. In the schematic diagram in Figure 1F, this relationship is depicted as "/" and the degree of importance of each signal is indicated with the thickness of the arrows."

(Page 5–6, line 39–8, in the revised manuscript)

"We further examined the GSH stress sensitivity of double or triple mutants of these three genes (Figure 1E). The Δ*bmh1*Δ*whi2* and Δ*rim11*Δ*bmh1* HGT1 strains showed augmented GSH stress sensitivity compared with their single-deletion mutants, as shown in Fig. 1E. Furthermore, *RIM11* overexpression did not rescue the GSH sensitivity of the Δ*bmh1* HGT1 strain, and *BMH1* overexpression did not rescue the GSH sensitivity of Δ*rim11* HGT1 (Figure 1F). These results suggest that Bmh1 may function in a different pathway than Rim11. Overexpression of *RIM11* partially rescued the GSH sensitivity of the Δ*whi2* HGT1 strain. Also, overexpression of *WHI2* rescued the GSH sensitivity of the Δ*rim11* HGT1 strain (Fig. 1F). These results suggest that Rim11 and Whi2 may function in the similar pathways. The Δ*whi2*Δ*rim11* HGT1 strain showed similar levels of sensitivity to Δ*rim11* HGT1 strain. In contrast, the Δ*whi2*Δ*rim11* HGT1 strain was more sensitive to GSH than the Δ*whi2* HGT1 strain (Fig. 1E). These results may suggest Rim11 has more important role than Whi2 in response to GSH stress."

Page 7 Line 20 – was there a specific reason the TDH3 promoter was used?

Response: Our intention with the use of the *TDH3* promoter was to express the Rim11 protein at a high level. We integrated the cassette containing the ORF under the control of the *TDH3* promoter and aimed to express it uniformly in all cells. Furthermore, the *TDH3* promoter maintains relatively high activity even after glucose depletion (Partow *et al*., 2010, Yeast, 27: 955 – 964).

Page 7 Line 30-32 – missing citation

Response: We have added the following reference in the main text.

(Page 8, line 1, in the revised manuscript)

Zhan *et al*., 2000.

Page 9 Lines 23-25- it is unclear what conditions this enrichment is occurring in.

Response: We revised the sentence in the main text as follows:

(Page 9, line 32–34, in the revised manuscript)

“In contrast, we performed GO-based analysis for 41 commonly downregulated DEGs in kinase-dead datasets (*i*), (*ii*), and (*iii*) (Figure 5C) under the same conditions mentioned above. Among these, the 14 downregulated genes were categorized as being involved in the lipid biosynthetic process (GO:0008610) (Figure 5D).”

The RNA-seq data would benefit from an examination of protein levels and lipid analysis to confirm the gene expression- enzyme product relationship.

Response: We selected *INO1* among the genes that exhibited distinctive changes in expression between conditions and analyzed its protein levels. The Ino1-GFP expression construct was integrated into the chromosome of the HGT1 strain and Ino1-GFP was detected using Western blotting with an anti-GFP antibody. GSH treatment of the HGT1 Rim11 strain significantly decreased the Ino1-GFP protein levels, which was consistent with the change in *INO1* transcription levels as revealed by RNA-seq analysis. This result is presented in Supplemental Figure 5B in the revised manuscript. Furthermore, in the presence of GSH, overexpression of *RIM11* increased the Ino1-GFP protein levels by 2.5-fold, and overexpression of *RIM1*-K68A decreased it by 11-fold. This result is consistent with the RNA-seq data of *INO1* in sample set (*iv*) and (*iii*), in which $\log_2$fc of HGT1 *RIM11* OE / HGT1 *RIM11* and of HGT1 *RIM11*-K68A OE / HGT1 *RIM11* OE were 1.84 and -4.37, respectively (Figure 5D, Table 2). We included this data as Figure 7A in the revised manuscript.

We performed the lipidomic analyses under different conditions and the results were added as Figures 7C and 7D in the revised manuscript. Principal component analysis revealed significant differences in lipid composition between four samples.

We have added these results in the main text as follows:

(Page 12, line 11–21, in the revised manuscript)

"Subsequently, lipidomic analysis was then performed to determine whether the observed transcriptional changes in the phospholipid biosynthetic genes lead to the changes in the phospholipid composition in the cell. A principal component analysis was performed to understand the overall pattern of phospholipid variability in terms of each biological condition (Figure 7C). The results clustered into four groups, suggesting that lipid compositions differed among four sample sets. When we looked at alterations in the phospholipid composition more closely (Figure. 7D), we found that PC, PE and PS contents in HGT1 Rim11 strain were significantly reduced by GSH treatment, which was consistent with decreases in the mRNA levels of *OPI3*, *PSD1* and *CHO1* as

revealed by the RNA-seq analysis (Supplemental Figure 5B). In contrast, a PI content was increased upon GSH treatment. Moreover, *RIM11* overexpression suppressed the reduction of PC, PE, and PS caused by GSH treatment and greatly increased the amount of PI. In contrast, overexpression of *RIM11*-K68A decreased suppressing effects on the reduction of these lipids. These results show that changes in the Rim11 kinase activity leads to changes in lipid compositions"

As we performed the lipidomic analysis in our revision, we deleted the following sentence in the original manuscript in the Discussion.
Deleted sentence (Page 14, line 26 – 28, in the original manuscript)
“Lipidomic analysis of RIM11-overexpressing cells may help to better understand the mechanisms by which yeast cells respond to GSH stress and a wide range of other environmental stresses.”

Figure 6B- Rim11 over expression only shows a partial rescue of the growth defect in ume6 deletion, not GSH stress resistance as stated in the text. This also doesn’t show conclusively that Rim11 and Ume6 act in the same pathway in GSH stress response. This figure could benefit from a spot test that included strains in a rim11Δ background to determine if there are synthetic growth defects when combined with ume6Δ.

Response: As suggested, our interpretation of the data regarding the test of *RIM11* overexpression in the Δ*ume6* HGT1 strain was incorrect. We corrected the main text as mentioned below. Additionally, as suggested, we created the Δ*rim11*Δ*ume6* HGT1 strain and tested its GSH stress sensitivity using a spot assay. We found that the Δ*rim11*Δ*ume6* HGT1 strain showed synthetic GSH sensitivity. We included this result as Figure 6D in the revised manuscript. This result suggests that Rim11 and Ume6 function in different pathways to cope with GSH stress. Based on these results, we revised the main text as follows.
(Page 10, line 23 – 26, in the original manuscript)
"In contrast, overexpression of *RIM11* in the Δ*ume6* HGT1 strain rendered it GSH stress resistant, although to a level lower than that of the HGT1 *RIM11* OE strain. These results suggest that Rim11 substrates other than Ume6 may be involved in GSH stress tolerance, but that Ime1 may likely be ruled out as a potential candidate."

(Page 11, line 11–13, in the revised manuscript)
"In contrast, *RIM11* overexpression in the Δ*ume6* HGT1 strain only partially rescued the growth defect of the Δ*ume6* HGT1 strain under GSH stress (Figure 6C). Furthermore, the Δ*rim11*Δ*ume6* HGT1 strain showed synthetic GSH sensitivity (Figure 6D), suggesting that Rim11 and Ume6 function in different pathways to cope with GSH stress."

Page 11 Line 8- missing citation

Response: We added the missing reference "Xiao Y & Mitchell AP, 2000" in the main text (Page 11, line 24, in the revised manuscript)

“As previously reported (Xiao and Mitchell, 2000), shifting the vegetative cells into SPM…”

Reviewer #2 (Remarks to the Author):

Yasukawa et al performed a screen designed to isolate genes that modulate the growth defect in S. cerevisiae associated with the uptake of high level of glutathione from the medium (facilitated by high-level expression of the plasma membrane glutathione transporter). They describe the isolation of three multicopy suppressors (RIM11, BHM1, and WHI2). Initially the authors focus on how loss or overexpression of these three genes impacts the ability of cells to survive under conditions of increased glutathione uptake. Later, the authors focus on Rim11 and explore how Rim11 kinase activity alters transcriptional profiling connected with the exposure to GSH. The authors propose a model for the upregulation of a subset of genes involved in lipid biogenesis that is dependent on Rim11 kinase activity and occurs in response to the cellular uptake of exogenous glutathione. The authors go on to make a series of observations about various players connected to Rim11 signaling and/or the transcription of lipid biogenesis genes, including OPI1, UME6, IME1, and INO1. They report transcriptional changes observed in the presence of GSH, as well as growth differences in the presence, absence, or overexpression of these genes.

Overall, the manuscript contains several interesting observations that suggest unexpected signaling activated in the presence of exogenous GSH, a less explored "stressor". Yet, in its current form, the manuscript feels incomplete in its goal to outline a clear Rim11-dependent signaling output that is associated with GSH uptake. While the authors outline a model in Figure 7, this model relies heavily on relationships anticipated based on prior studies focused on the signaling outlined in a variety of conditions, not necessarily connections established by the authors to occur as part of GSH-induced signaling. In broad terms, it feels like a significant jump from the reported data to the model of Figure 7, including proteins in the model that are not discussed or explored in the manuscript. The extent of the experiments do not seem to match with the overall conclusions and model.

Response: We agree with the reviewer, and we have carefully revised the model figure (new Figure 8 in the revised manuscript) to reflect our data as accurately as possible. Moreover, we included Pah1, which plays an important role in the regulation of phospholipid biosynthesis and whose enzymatic activity is downregulated by phosphorylation by Rim11. We have addressed each of the specific comments below.

Some suggestions as to further studies that may help to focus or complete the study:

1. Arguably, the manuscript could benefit from some reorganization, with a clear focus from the initial results on Rim11. For example, reporting the initial isolation of RIM11 as a high-copy

suppressor, and a straightforward analysis of whether overexpression of RIM11 requires Ire1 to confer resistance to GSH in the HGT1 strain, would streamline the results. These straightforward experiments would establish a role for Rim11, and a role that is outside the prior focus on the UPR that the Toledano work reported in Kumar et al 2011.

Response: We agree that reorganization may be meaningful. However, as we believe that discovery of two other suppressors is important for future studies on GSH stress, we hope to show the data we obtained regarding the relationship between the suppressors (Figure 1). We have carefully revised the main text related to Figure 1 to explain the results clearly as follows.

(Page 5–6, line 39–8, in the revised manuscript)

"We further examined the GSH stress sensitivity of double or triple mutants of these three genes (Figure 1E). The Δ*bmh1*Δ*whi2* and Δ*rim11*Δ*bmh1* HGT1 strains showed augmented GSH stress sensitivity compared with their single-deletion mutants, as shown in Fig. 1E. Furthermore, *RIM11* overexpression did not rescue the GSH sensitivity of the Δ*bmh1* HGT1 strain, and *BMH1* overexpression did not rescue the GSH sensitivity of Δ*rim11* HGT1 (Figure 1F). These results suggest that *Bmh1* may function in a different pathway than Rim11. Overexpression of *RIM11* partially rescued the GSH sensitivity of the Δ*whi2* HGT1 strain. Also, overexpression of *WHI2* rescued the GSH sensitivity of the Δ*rim11* HGT1 strain (Fig. 1F). These results suggest that Rim11 and Whi2 may function in the similar pathways. The Δ*whi2*Δ*rim11* HGT1 strain showed similar levels of sensitivity to Δ*rim11* HGT1 strain. In contrast, the Δ*whi2* Δ*rim11* HGT1 strain was more sensitive to GSH than the Δ*whi2* HGT1 strain (Fig. 1E). These results may suggest Rim11 has more important role than Whi2 in response to GSH stress."

As per your suggestion, *RIM11* was overexpressed on a high-copy plasmid in the Δ*ire1* HGT1 strain and sensitivity to GSH stress was tested. We demonstrated that *IRE1* is not necessary for *RIM11* to function as a multicopy suppressor to GSH stress (new Figure 3D). This result strengthens our conclusion, which we have written as follows:

"This result strongly suggests that Rim11 functions independently of the Ire1-dependent UPR". (page 7, line 6–7, in the revised manuscript).

We thank you for these helpful suggestions.

We have included this result as Figure 3D in the revised manuscript. Accordingly, descriptions were added to the main text and the Figure 3D legend as follows.

The main text: (page 7, line 20–22, in the revised manuscript)

"*RIM11* was overexpressed on a high-copy plasmid in the Δ*ire1* HGT1 strain and its sensitivity to

GSH stress was tested. We found that *IRE1* is not necessary for *RIM11* to function as a multicopy suppressor of GSH stress (Figure 3D).”

The figure legend (Figure 3; page 29, line 5–6, in the revised manuscript):
“(D) *RIM11* was overexpressed on a high-copy plasmid in the Δ*ire1* HGT1 strain and sensitivity to GSH stress was tested.”

2. As the manuscript is currently setup, the authors make epistasis arguments about the three initially isolated suppressors from the spotting assay in Figure 1E. These conclusions appear to be a potential overinterpretation of the data. It is also unclear what the pathway organization (if true) means in the context of the later analysis of, and focus on, Rim11.

The growth defects observed between the three individual mutants on plates are modest and seem variable (Fig 1D the bhm1Δ and whi2Δ growth are called similar, versus in Fig 1E the bmh1Δ shows better growth than the whi2Δ). This growth variability makes the argument as to whether there is a true additive impact, or if the difference in growth is just assay-to-assay variation, difficult to discern. Similarly, the growth suppression of the null strains with the various 2μ plasmids is suggestive, but not particularly robust or fully consistent with the outlined model. It is unclear in the outlined epistasis diagram why a bhm1Δ would not be bypassed by additional RIM11 (?) If the authors do want to make arguments about epistasis, some evidence of reproducibility is also warranted, including how many replicates were performed and/or showing replicates of the strains.
Response: To address "Fig 1D the bhm1Δ and whi2Δ growth are called similar, versus in Fig 1E the bmh1Δ shows better growth than the whi2Δ", we further tested the growth in the liquid medium and performed a spot assay. The results are shown in Figure for the reviewers B. These results confirmed that the Δ*whi2* HGT1 strain was more sensitive to GSH than was the Δ*bmh1* HGT1 strain.

Accordingly, the following underlined sentence was added to the main text.
(Page 5, line 28–32 in the revised manuscript)
“Deleting *RIM11*, *BMH1*, or *WHI2* rendered the HGT1 strain sensitive to GSH stress. Introduction of *BMH1* and *WHI2* on a low-copy plasmid rescued the growth retardation phenotypes of the corresponding deletion mutants (Figure 1D). The Δ*rim11* HGT1 strain showed slightly higher sensitivity to GSH than the Δ*whi2* HGT1 strain, whereas the Δ*bmh1* HGT1 strain was less sensitive than the other two strains. Although the introduction of *RIM11* under the control of its own promoter failed to rescue the growth phenotype of the Δ*rim11* HGT strain, “

The lower panel of Figure 1D was also replaced with a figure that showed the results with improved

clarity.

We agree that there was an overinterpretation of the data in the original manuscript. As we had already repeated the same experiments more than twice, the results are shown in the Figures for the reviewers C (replicates of Figure 1E in the main text) and in the Figures for the reviewers D (replicates of Figure 1F in the main text). In the Figures for the reviewers D, spot assays using at least three independent isolates in every combination were performed and the results were reproducible.

Accordingly, we added the following sentence to the Figure 1 legend.
(Page 28; Line 13–14, in the revised manuscript).
"Spot assays in Figure 1 were repeated more than twice and the results were reproducible."

We avoided overinterpretation of the data and revised the manuscript to explain the results more accurately than in the original manuscript. We then carefully considered the spot assay results and concluded that it was difficult to create a diagram that reflected all the data correctly. Therefore, we have deleted the diagram in the revised manuscript.

We changed the main text as follows.
(Page 5–6, line 39–8, in the revised manuscript)
"We further examined the GSH stress sensitivity of double or triple mutants of these three genes (Figure 1E). The Δ*bmh1*Δ*whi2* and Δ*rim11*Δ*bmh1* HGT1 strains showed augmented GSH stress sensitivity compared with their single-deletion mutants, as shown in Fig. 1E. Furthermore, *RIM11* overexpression did not rescue the GSH sensitivity of the Δ*bmh1* HGT1 strain, and *BMH1* overexpression did not rescue the GSH sensitivity of Δ*rim11* HGT1 (Figure 1F). These results suggest that Bmh1 may function in a different pathway than Rim11. Overexpression of *RIM11* partially rescued the GSH sensitivity of the Δ*whi2* HGT1 strain. Also, overexpression of *WHI2* rescued the GSH sensitivity of the Δ*rim11* HGT1 strain (Fig. 1F). These results suggest that Rim11 and Whi2 may function in the similar pathways. The Δ*whi2*Δ*rim11* HGT1 strain showed similar levels of sensitivity to Δ*rim11* HGT1 strain. In contrast, the Δ*whi2* Δ*rim11* HGT1 strain was more sensitive to GSH than the Δ*whi2* HGT1 strain (Fig. 1E). These results may suggest Rim11 has more important role than Whi2 in response to GSH stress."

3. The authors do not validate the transcriptional upregulation they outline for their RNA-seq dataset. Some validation by RT-PCR would strengthen the conclusions. It would also allow one to establish if Rim11 is required for the upregulation (e.g., analysis in a rim11Δ). Such data would

complement the focus on growth as the major determinant for a signaling role.

Response: We agree that confirming the RNA-seq results by other techniques for RNA quantification is helpful. Therefore, we reanalyzed the DNA microarray data we obtained before. In the HGT1 Rim11 OE strain, we found that mRNA levels of *INO1*, *MTH1*, *ULI1* and *CHO1*, which showed a distinctive increase or decrease in the RNA-seq analysis, changed in the same manner as the DNA microarray results. These results are shown in Table A for the reviewers. Moreover, comparison of gene expression changes between HGT1_GSH+ and Δ*rim11* HGT1_GSH+ revealed that disruption of the *RIM11* gene lowered the mRNA levels of *MTH1*, *ULI1* and *CHO1* genes. In contrast, *INO1* mRNA increased by 1.2-fold (log_2fc of 0.27) in the DNA microarray. We also quantitated the abundance of Ino1-GFP using western blotting and found that a protein amount of Ino1-GFP was positively correlated with the kinase activity of Rim11 (Figure 7A). The result demonstrated that changes in Ino1 protein abundance reflected its mRNA levels well. This means that Rim11 has a positive function in the expression of these genes, which is consistent with the suppressor activity of Rim11 that upregulates a set of genes including these four genes (Fig. 5D).

We revised the main text as follows.

(Page 14, Line 34–39, in the revised manuscript)
"At the protein level, Ino1-GFP was significantly decreased upon GSH treatment (Figure 7A. Moreover, PC, PE, and PS contents were decreased upon GSH treatment (Figure 7D). We hypothesize that Rim11 translocates into the nucleus following GSH treatment (Figure 4C, Supplemental Fig. 4C), and phosphorylation of Pah1 by Rim11 is decreased. We predict that reactivated Pah1 converts PA to DAG, which leads to the reduction of PA levels and translocation of Opi1 into the nucleus, ultimately leading to repression of the transcription of the phospholipid biosynthetic gene (Figure 8B)."

(Page 15, Line 5–11 in the revised manuscript)
"The amount of Ino1-GFP was consistent with the changes in the *INO1* mRNA level (Figure 7A). Moreover, lipidomic analyses revealed that overexpression of *RIM11* increased the overall lipid contents in the cell (Figure 7D). Therefore, we hypothesized that de-repression of the decreased biosynthetic activity of phospholipids by Rim11, and the increased expression of the *INO1* gene, may contribute to enhanced tolerance of the cells to GSH. Specifically, overexpressed Rim11 phosphorylates Pah1, thereby inhibiting the dephosphorylating activity of Pah1. We hypothesize that the proportion of Opi1, which is anchored on the ER membrane, then increases, causing transcriptional de-repression of the phospholipid biosynthetic genes."

4. The experiments of Figure 6 exploring Opi1, Ime1, Ume6 and Ino1 expression or localization during conditions of increased GSH are suggestive of unique roles. However, these data could be more robust. The variable expression of Opi1 in the "GSH stress" cells makes the co-localization data difficult to parse.

Response: The ratio of cells with Opi1-GFP in the nucleus were counted and graphed. This graph was added to the revised manuscript as Figure 6B. As Opi1-GFP is expressed from the construct that is integrated in the chromosome, a reason for variable expression, as you suggested, is not clear. However, we feel that the quantification of the nuclear localization upon GSH stress strengthens our original conclusion that GSH treatment induces nuclear localization of Opi1. We added this result in the revised manuscript as follows.

(Page 10–11, Line 39–2, in the revised manuscript):

” The ratio of cells with GFP-Opi1 in the ER or nucleus were counted and graphed. Nuclear localization of GFP-Opi1 was significantly increased not only by glucose starvation, but also upon GSH treatment (Figure 6B and Supplemental Figure 6C), which suggests that Opi1 may be involved in the GSH stress response and represses the transcription of phospholipid biosynthetic genes.”

Moreover, there was a mistake in the original manuscript regarding GFP-*OPI1* expression. In Figure 6B, GFP-*OPI1* was introduced into the cell by homologous recombination, not on the plasmid. Accordingly, we deleted “harboring pRS316 empty vector” in the Figure 6B legend in the original manuscript as indicated below.

(Page 30; line 23–24, in the revised manuscript)

"To determine the level of background fluorescence or autofluorescence originating from the live cells, images of HGT1 strain ~~harboring pRS316 empty vector~~ under GSH stress conditions were also acquired."

It is not clear why a more intense western band for Ume6-FLAG implies an increased in"Ume6 phosphorylation".

Response: To further test the possibility of phosphorylation of Ume6, we conducted experiments using biotinylated-Phos-tag and streptavidin-conjugated Alexa488. We immunoprecipitated 3xFLAG-Ume6, which was then separated by SDS-PAGE. Proteins were transferred to the membrane, which was probed with biotinylated-Phos-tag and streptavidin-conjugated Alexa488. A yeast protein known to be phosphorylated (whose identity we want to avoid revealing here because a manuscript is in preparation) was also included as a positive experimental control. Under various conditions, phosphorylation of positive control protein was always detected. However,

phosphorylation of 3xFLAG-Ume6 was never clearly detected under the same experimental conditions.

Therefore, we concluded that there was a low possibility that Ume6 was sufficiently phosphorylated to be detected by the method we employed. Accordingly, we have deleted "Ume6 phosphorylation" (page 11, line 11 in the original manuscript). We thank you for bringing this to our attention.

5. The authors observe and say that the degree and/or location of phosphorylation on Rim11 is likely distinct for GSH and glucose starvation, but yet then the authors argue that GSH induces a glucose starvation response (in the discussion). If the authors wish to imply that glucose transporters, etc. are important players in the response to GSH (as discussed), then some experiments to this point should be performed.

Response: To analyze the behavior of glucose transporters in response to GSH addition, we created yeast strains in which there were glucose transporters to which GFP is C-terminally fused. Upon overexpression of *RIM11*, the protein abundance of Hxt1, a low-affinity glucose transporter, was decreased, and that of Hxt2, a high-affinity glucose transporter, was increased. It seems likely that this is one of the mechanisms by which *RIM11* alleviates GSH stress as a high-copy suppressor. GSH stress did not significantly change the protein abundance of either Hxt1-GFP or Hxt2-GFP. However, there was a tendency for Hxt1-GFP to increase and a tendency for Hxt2-GFP to decrease ($p = 0.059$, $n = 3$) upon GSH treatment. Furthermore, microscopic measurements revealed a significant increase of Hxt1-GFP signal at the plasma membrane upon GSH treatment. Results of the western blotting were included in Figure 6A and microscopic observations were included in Supplement Figure 6A and 6B in the revised manuscript. We also revised the main text as follows:

(Page 10, line 15–30, in the revised manuscript)

"…we first examined the regulatory factor involved in glucose sensing. During *RIM11* OE-mediated GSH stress tolerance, a signal induced by the overexpressed *RIM11* greatly increased the expression of *MTH1* (log_2fc of 2.42) via unknown mechanisms. In contrast, overexpression of *RIM11 K68A* substantially decreased (log_2fc of –3.09) *MTH1* expression (Figure 5D and Supplemental Table 2, see sample set [*iii*] and [*iv*]). Furthermore, the expression of the low-affinity glucose transporter *HXT1* was repressed (log_2fc of –3.12), whereas that of the high-affinity glucose transporter *HXT2* was markedly upregulated (log_2fc of 3.80) (Supplemental Table 2, see sample set [*iv*]). Considering that Mth1 also represses the expression of *HXT1*, a gene encoding a low-affinity glucose transporter, in the presence of high glucose (Roy *et al.*, 2013), we hypothesized that a relationship may exist between the kinase activity of Rim1 and the dynamics of hexose transporters. To analyze the dynamics of glucose transporters in the GSH stress response, we created yeast strains producing glucose transporters to which GFP was fused c-terminally. RIM11 overexpression decreased the

protein abundance of Hxt1, a low-affinity glucose transporter, and increased that of Hxt2, a high-affinity glucose transporter (Figure 6A). We hypothesized that this may be one of the mechanisms by which *RIM11* alleviates GSH stress as a high-copy suppressor. We observed a tendency for Hxt1-GFP to increase and Hxt2-GFP to decrease ($p = 0.059$, $n = 3$) upon GSH treatment (Figure 6A). Moreover, microscopic measurements revealed a significant increase of Hxt1-GFP signal at the plasma membrane upon GSH treatment (Supplemental Figure 6A and 6B). Therefore, these transcriptional and proteomic changes were presumed to alleviate the stress caused by low glucose."

Or at a minimum, the transcriptional signature that the authors use in the discussion to support a glucose starvation response should be more clearly outlined in the results.

Response: As per your suggestion, we have relocated the sentence regarding Table S2 in the Discussion to the Result section.

The following two sentences have been relocated to the Results section:

(Page 12, Line 33 in the original manuscript):

Mth1 also represses the expression of *HXT1*, a gene encoding a low-affinity glucose transporter, in the presence of high glucose (Roy *et al.*, 2013).

(Page 12, Line 39 in the original manuscript)

" During *RIM11* OE-mediated GSH stress tolerance (Figure 7C), a signal induced by the overexpressed Rim11 greatly increased the expression of *MTH1* (log_2fc of 2.42) by unknown mechanisms (Supplemental Table 2, see sample set [*iv*]). Concomitantly, the expression of the low-affinity glucose transporter *HXT1* was repressed (log_2fc of -3.12), whereas that of the high-affinity glucose transporter *HXT2* was markedly upregulated (log_2fc of 3.80) (Supplemental Table 2, see sample set [*iv*]). Therefore, we believe that these transcriptional changes alleviate the stress caused by low-glucose levels and the transcriptional repression by Opi1."

6. The RNA-seq were completed after 2 h exposure to 250 µM GSH. The authors state that these data are "consistent with the results reported previously" of Kumar et al 2011. It seems a missed opportunity for a more descriptive comparison. Given the prior work followed the transcriptional response at an earlier and lower dose (50 µM GSH for 30 min), one would expect some differences.

Response: As per your suggestion, we once again compared the DNA microarray result of Kumar et al (2011) with those of ours. The most apparent difference was the expression of the *INO1* gene. It was increased by 4.168-fold in Kumar et al. (2011) and was decreased by 3.869-fold in our results.

As the number of genes examined were much smaller in Kumar et al. (2011) than in our experiment (408 genes vs 5,142 genes, respectively), and in the former, *INO1* and *HNM1* were the only genes containing the UAS sequence, we found it difficult to draw general conclusions from the two results. Furthermore, there is a difference in the methods used, DNA microarray (Kumar et al. 2011) vs. RNA-seq analysis (our experiments). This may also make direct comparison difficult. However, analyzing transcriptomic changes under different concentrations of GSH or treatment times may be important to further understand the GSH stress response.

We changed sentences in the revised manuscript as follows:

(Page 15, line 26–36, in the revised manuscript)

"We then compared our RNA-seq results (250 μM GSH for 120 min) with the DNA microarray results (50 μM GSH for 30 min) reported by Kumar *et al*., (2011). In contrast to the current results showing the decrease of *INO1* mRNA by 3.9-fold following GSH addition, previous reports showed that transcription of the *INO1* gene was increased by 4.2-fold after GSH addition (Kumar *et al*., 2011). Considering that *INO1* and *HNM1* were the only genes containing the UAS_{INO} sequence in Kumar *et al*. (2011) and expression of no other genes containing the UAS_{INO} sequence were analyzed, involvement of Opi1 and the reason for the increase in *INO1* mRNA by GSH is unclear. We hypothesize that it may either be due to the different strains used in the two studies (YPH499 in Kumar et al. (2011) vs BY4741 in the current study), different conditions of GSH treatment (50 μM GSH for 30 min in Kumar *et al*. (2011) vs 250 μM GSH for 120 min in the current results), or the different expression levels of *HGT1*, which leads to different intracellular concentrations of GSH. We found it difficult to draw general conclusions from the two results. However, analyzing transcriptomic changes under different concentrations of GSH or treatment time may be important to further understand the GSH stress response."

7. Minor point. The authors convincingly establish that HGT1 overexpression confers sensitivity to exogenous GSH as reported previously. Yet, it is unclear what the % quantification of GSH (8-9% intracellular GSH concentration) refers to in Figure 1B. One presumes this is 8-9% of the total GSH added to the culture?

Response: We apologize for the unclear description. To clarify these points, we changed the sentences as follows:

(Page 5, Line 13–14, in the revised manuscript)

"Intracellular GSH content plateaued (8–9% per dry-cell-weight) at the 4-h time point in the growth medium containing more than 250 μM GSH,…"

Y-axis of Figure 1B in the revised manuscript has been labeled as "GSH content per dry-cell-weight

[%]."

Revision, authors' response

Figures for reviewers:

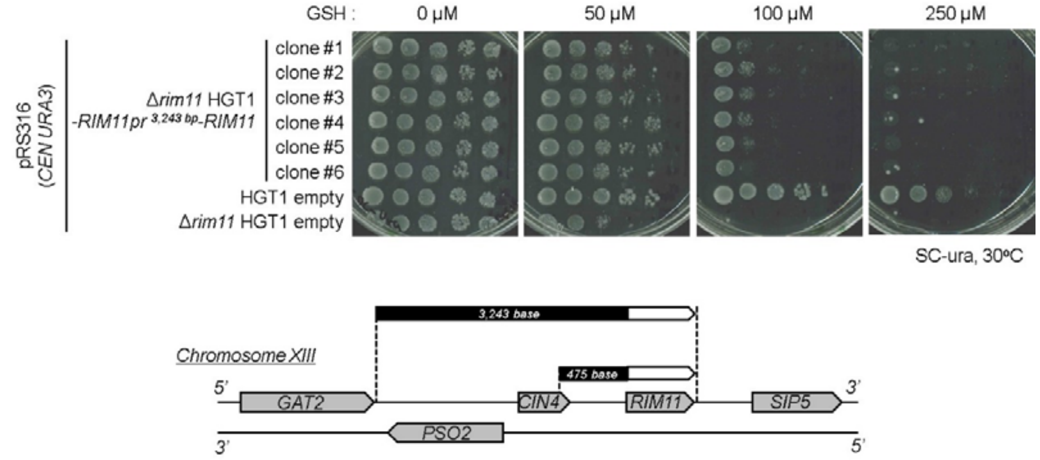

Fig. A: Growth of the Δ*rim11* HGT1 strains carrying *RIM11* with its upstream region (3,243 base) on the plasmid (*CEN URA3*) in the absence or presence of GSH treatment. Six clones were randomly chosen and their sensitivities to GSH-induced stress were evaluated using a spot test on SC-ura plates. Schematic diagram shows the cloned length of the upstream region of *RIM11*. Base 475 is an upstream region from the open reading frame of *RIM11* in the Δ*rim11* HGT1 *RIM11pr-RIM11* (CEN) strain displayed in the main Figure 1D.

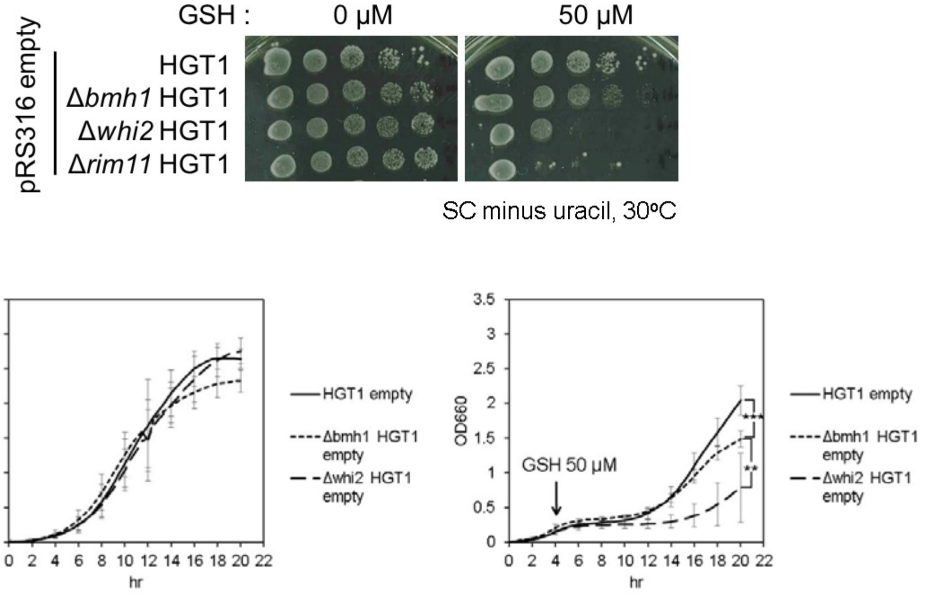

Fig. B: Comparison of the growth of the Δ*bmh1* HGT1 and Δ*whi2* HGT1 strain with or without 50 μM GSH. Strains were cultured in SC without uracil at 30°C. Spot test (upper) was performed with two independent experiments and a representative image is shown. During the growth time course (lower), cells (OD_{600} of 0.1) were cultivated for 4 h and GSH was added. Line plots and error bars represent mean and standard deviation from three independent experiments (n = 9), respectively. Significant differences were analyzed using two-tailed Welch's *t*-test, ** $p < 0.01$, *** $p < 0.001$.

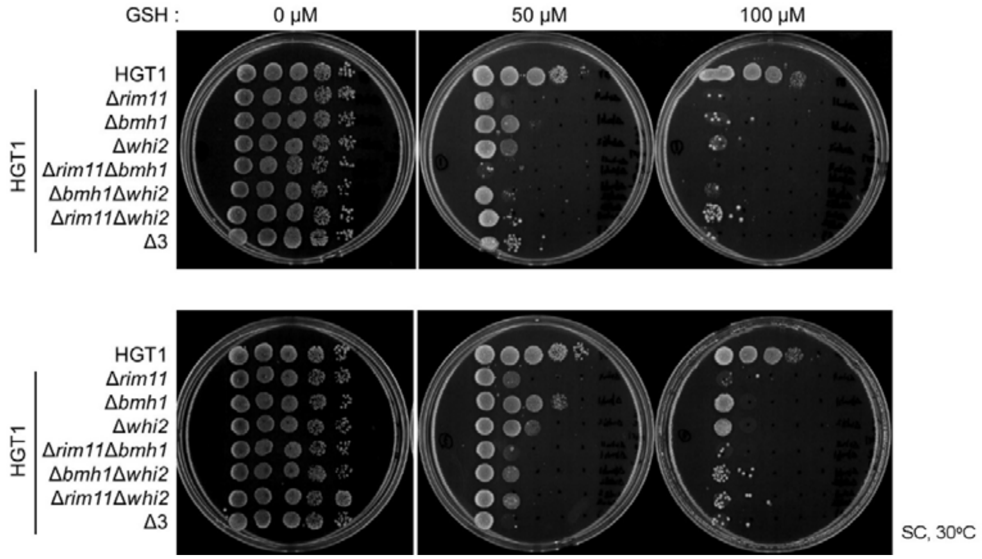

Fig. C: Other independent repeated experiments related to Figure 1E in the main manuscript. Second and third replicates, different from that presented in FIGURE 1E, are shown.

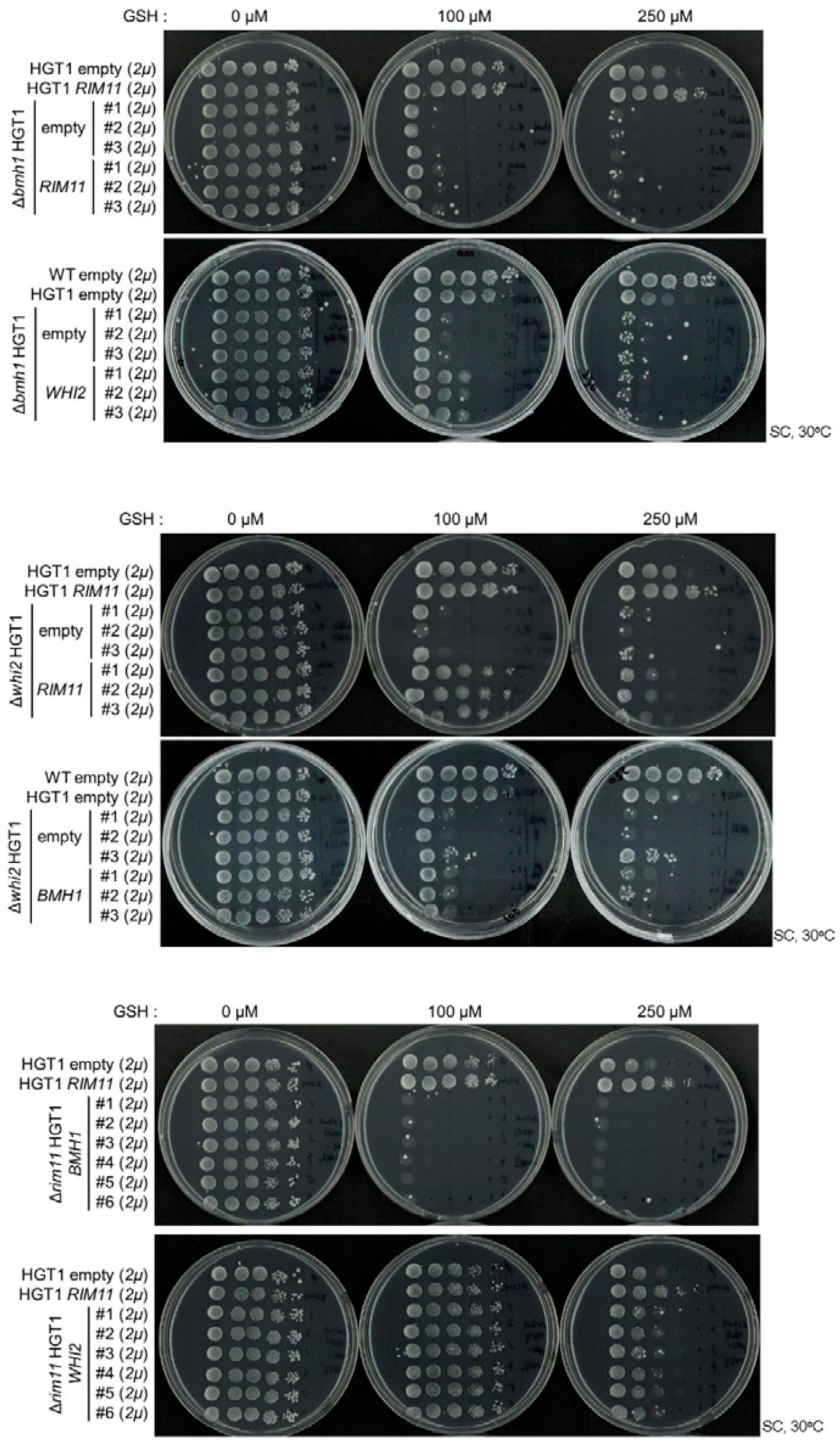

Fig. D: The number of biological replicates per sample with regards to Figure 1F in the main manuscript.

Method	Two-group comparison	*INO1*	*CHO1*	*MTH1*	*ULI1*
DNA microarray	Δ*rim11* HGT1 / HGT1	0.27	-0.39	-1.78	-0.39
DNA microarray	HGT1 Rim11 OE / HGT1	2.26	1.32	1.64	3.10
RNA-seq (Figure 5B, sample set (*iv*))	HGT1 Rim11 OE / HGT1	1.84	0.80	2.42	2.65

Table A: Logarithmic (base 2) values of the differentially expressed genes obtained from DNA microarray and RNA-seq. For the DNA microarray experiment, total RNA samples extracted from HGT1 and the Δ*rim11* HGT1 strains under GSH-induced stress (250 µM) were purified. cDNA from total RNA (200 ng) of each biological samples (n = 2) was labeled with Cys3, and subsequently hybridized to an 8 × 15 K Agilent microarray format (G4813A#16322, Yeast *Saccharomyces cerevisiae*). The transcript levels reflected the average values for each biological sample (n = 2) and are shown as the $\log_2$fc values.

RE: Manuscript #E23-03-0116R
TITLE: "Yeast Rim11 kinase responds to glutathione-induced stress by regulating the transcription of phospholipid biosynthetic genes"

Dear Dr. Noda:

Thank you for submitting this revision, which has now been seen by the two original reviewers whose comments are found below. Both reviewers are supportive of publication, with a couple of minor changes suggested by reviewer 1. I'm happy to provisionally accept the study in advance of these changes.

Sincerely,
Elizabeth Miller
Monitoring Editor
Molecular Biology of the Cell

Dear Dr. Noda,

The review of your manuscript, referenced above, is now complete. The Monitoring Editor has decided that your manuscript requires minor revisions before it can be published in Molecular Biology of the Cell, as described in the Monitoring Editor's decision letter above and the reviewer comments (if any) below.

A reminder: Please do not contact the Monitoring Editor directly regarding your manuscript. If you have any questions regarding the review process or the decision, please contact the MBoC Editorial Office (mboc@ascb.org).

When submitting your revision include a rebuttal letter that details, point-by-point, how the Monitoring Editor's and reviewers' comments have been addressed. (The file type for this letter must be "rebuttal letter"; do not include your response to the Monitoring Editor and reviewers in a "cover letter.") Please bear in mind that your rebuttal letter will be published with your paper if it is accepted, unless you have opted out of publishing the review history.

Authors are allowed 15 days to submit a revision. If this time period is inadequate, please contact us immediately at mboc@ascb.org.

In preparing your revised manuscript, please follow the instruction in the Information for Authors (www.molbiolcell.org/info-for-authors). In particular, to prepare for the possible acceptance of your revised manuscript, submit final, publication-quality figures with your revision as described.

If your manuscript contains a Significance Statement, please make sure that it consists of three separate bullet points totaling a maximum of 100 words and addressing each of the following points (see https://www.molbiolcell.org/curation-tools):
First bullet: What is the background context? What gap in knowledge does this study addres?
Second bullet: What are the key findings? What is unique or new about the approach?
Third bullet: Why is this paper significant? How might it influence future research?

To submit the rebuttal letter, revised version, and figures, please use this link (please enable cookies, or cut and paste URL):
Link Not Available

MBoC offers the option to publish your work with immediate open access. Open access can increase the discoverability and usability of your research. If you would like to publish your paper with immediate open access but did not select this option during initial submission, please contact us at mbc@ascb.org..

Authors of Articles and Brief Communications whose manuscripts have returned for minor revision ("revise only") are encouraged to create a short video abstract to accompany their article when it is published. These video abstracts, known as Science Sketches, are up to 2 minutes long and will be published on YouTube and then embedded in the article abstract. Science Sketch Editors on the MBoC Editorial Board will provide guidance as you prepare your video. Information about how to prepare and submit a video abstract is available at www.molbiolcell.org/science-sketches. Please contact mboc@ascb.org if you are interested in creating a Science Sketch.

Thank you for submitting your manuscript to MBoC. Please do not hesitate to contact this office if you have any questions.

Sincerely,

Eric Baker
Journal Production Manager
MBoC Editorial Office
mbc@ascb.org

Reviewer #1 (Remarks to the Author):

This manuscript from Yusukawa et al., which is a revision of a previous manuscript, advances the understanding of the gluthatione stress response in yeast and gives insight into the significance of phospholipid metabolism in this response. This revision addresses most of the major concerns, including the lack of data showing that differentially expressed genes identified by RNA-seq are confirmed by examining protein levels, as well as the absence of data looking at cellular phospholipid content. Although most of the initial comments were adequately addressed, there remains an issue with Figure 8, which in panel A and C appear to show Rim11-dependent phosphorylation of Pah1 resulting in Pah1 translocation from the nuclear/ER membrane and being subject to proteasomal degradation. This model is inconsistent with Khondker et al. 2022. Although Khondker et al. showed that Rim11-dependent phosphorylation of Pah1 resulted in inhibition of catalytic activity, there was no significant on Pah1 localization or abundance, and thus this figure is misleading. I suggest that Rim11 be placed on the opposite arrow leading to the Nem1-Spo7 complex as shown in the Khondker et al paper.

Minor comments:
Page 11, Line 35: Strain name does not follow proper nomenclature (should be RIM11)
Page 11, Line 38: RIM11 is written as RIM1

Reviewer #2 (Remarks to the Author):

The authors have addressed my primary concerns.

As a point of potential issue moving forward - the conversion of the supplemental data file resulted in some figure rearrangement. The docx file is fine.

Reviewer #1 (Remarks to the Author):

This manuscript from Yusukawa et al., which is a revision of a previous manuscript, advances the understanding of the gluthatione stress response in yeast and gives insight into the significance of phospholipid metabolism in this response. This revision addresses most of the major concerns, including the lack of data showing that differentially expressed genes identified by RNA-seq are confirmed by examining protein levels, as well as the absence of data looking at cellular phospholipid content.

Although most of the initial comments were adequately addressed, there remains an issue with Figure 8, which in panel A and C appear to show Rim11-dependent phosphorylation of Pah1 resulting in Pah1 translocation from the nuclear/ER membrane and being subject to proteasomal degradation. This model is inconsistent with Khondker et al. 2022. Although Khondker et al. showed that Rim11-dependent phosphorylation of Pah1 resulted in inhibition of catalytic activity, there was no significant on Pah1 localization or abundance, and thus this figure is misleading. I suggest that Rim11 be placed on the opposite arrow leading to the Nem1-Spo7 complex as shown in the Khondker et al paper.

Response: Thank you for these helpful discussions. To accurately reflect the known roles of Pah1 phosphorylation, we have moved Rim11 in panel A and C in Figure 8 to the place close to the arrow leading to the Nem1-Spo7 complex as suggested.

Minor comments:

Page 11, Line 35: Strain name does not follow proper nomenclature (should be RIM11)

Page 11, Line 38: RIM11 is written as RIM1

Response: We have collected these two errors pointed out by this reviewer in the revised manuscript.

Reviewer #2 (Remarks to the Author):

The authors have addressed my primary concerns.
Response: Thank you very much!

As a point of potential issue moving forward - the conversion of the supplemental data file resulted in some figure rearrangement. The docx file is fine.
Response: We have collected the docx file to prevent the rearrangement upon conversion to PDF.

RE: Manuscript #E23-03-0116RR
TITLE: "Yeast Rim11 kinase responds to glutathione-induced stress by regulating the transcription of phospholipid biosynthetic genes"

Dear Dr. Noda:

I am pleased to accept your manuscript for publication in Molecular Biology of the Cell.

Sincerely,
Elizabeth Miller
Monitoring Editor
Molecular Biology of the Cell

Dear Dr. Noda:

Congratulations on the acceptance of your manuscript! Thank you for choosing to publish your work in Molecular Biology of the Cell (MBoC).

Within 10 days, an unedited PDF of your manuscript will be published on MBoC in Press, an early release version of the journal. The date your manuscript appears on this site is the official publication date.

Your copyedited and typeset manuscript will be scheduled for publication in the next available issue of MBoC. Our production team will notify you when the page proofs of your paper are ready for your review.

MBoC offers the option to publish your paper with immediate open access. Open access can increase the discoverability and usability of your research. If you would like to publish your paper with immediate open access but did not select this option during initial submission, please contact the MBoC Editorial Office (mbc@ascb.org).

We invite you to submit images related to your accepted manuscript to be considered for the journal cover. Please contact mboc@ascb.org to learn how to submit candidate cover images.

Authors of Articles and Brief Communications are also encouraged to create a short video abstract to accompany their article when it is published. These video abstracts, known as Science Sketches, are up to 2 minutes long and will be published on YouTube and then embedded in the article abstract. Science Sketch Editors on the MBoC Editorial Board will provide guidance as you prepare your video. Information about how to prepare and submit a video abstract is available at www.molbiolcell.org/science-sketches. Please contact mboc@ascb.org if you are interested in creating a Science Sketch.

We look forward to publishing your paper in MBoC.
Sign up for MBoC's new content alerts here https://tinyurl.com/3bcsyk3e

Sincerely,

Eric Baker
Journal Production Manager
MBoC Editorial Office
mbc@ascb.org
